# Molecular recording of cellular protein kinase activity with chemical labeling

**DOI:** 10.1038/s41589-025-01949-6

**Published:** 2025-07-10

**Authors:** De-en Sun, Siu Wang Ng, Yu Zheng, Shu Xie, Niklas Schwan, Paula Breuer, Dirk C. Hoffmann, Julius Michel, Daniel D. Azorin, Kim E. Boonekamp, Frank Winkler, Wolfgang Wick, Michael Boutros, Yulong Li, Kai Johnsson

**Affiliations:** 1 Department of Chemical Biology, Max Planck Institute for Medical Research, Heidelberg, Germany; 2 German Cancer Research Center, Division Signaling and Functional Genomics, Heidelberg, Germany; 3 Institute of Human Genetics, Medical Faculty Heidelberg, Heidelberg University, Heidelberg, Germany; 4 Department of Cell and Molecular Biology, Medical Faculty Mannheim and BioQuant, Heidelberg University, Heidelberg, Germany; 5 State Key Laboratory of Membrane Biology, School of Life Sciences, Peking University, Beijing, China; 6 PKU-IDG/McGovern Institute for Brain Research, Beijing, China; 7 Peking-Tsinghua Center for Life Sciences, New Cornerstone Science Laboratory, Academy for Advanced Interdisciplinary Studies, Peking University, Beijing, China; 8 Clinical Cooperation Unit Neurooncology, German Cancer Consortium, German Cancer Research Center, Heidelberg, Germany; 9 Department of Neurology and Neurooncology Program, National Center for Tumor Diseases, Heidelberg University Hospital, Heidelberg, Germany; 10 Institute of Chemical Sciences and Engineering, École Polytechnique Fédérale de Lausanne, Lausanne, Switzerland; 11 Present Address: Department of Biosystems Science and Engineering, ETH Zurich, Basel, Switzerland

**Keywords:** Chemical tools, Imaging, Kinases

## Abstract

Protein kinases control most cellular processes and aberrant kinase activity is involved in numerous diseases. Here we introduce molecular recorders of kinase activities for later analysis to investigate the link between specific kinase activities and cellular phenotypes in heterogeneous cell populations and in vivo. Based on split-HaloTag and a phosphorylation-dependent molecular switch, our recorders become rapidly labeled in the presence of a specific kinase activity and a fluorescent HaloTag substrate. The kinase activity in a given cell controls the degree of fluorescent labeling, whereas the recording window is set by the presence of the fluorescent substrate. We designed specific recorders for four protein kinases, including protein kinase A. We apply our protein kinase A recorder to sort heterogeneous cell populations for subsequent transcriptome analysis, in genome-wide CRISPR screens to discover regulators of PKA activity and to track neuromodulation in freely moving mice.

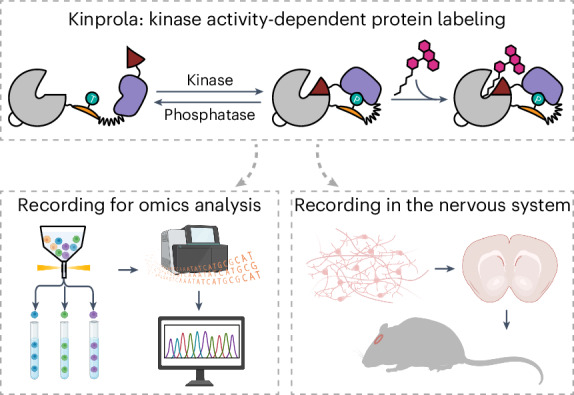

## Abstract (web summary)

Molecular recorders based on kinase activity-dependent protein labeling track specific kinase activities to understand their link to cellular phenotypes in heterogeneous cell populations and in vivo.

## Main

Protein kinase signaling cascades are essential in nearly all cellular processes. Aberrant kinase signaling, which disrupts the balance of protein phosphorylation, is tightly linked to tumorigenesis and neuronal dysfunction^1^. A prototypical kinase is cAMP-dependent protein kinase (that is, protein kinase A (PKA)), a major cAMP effector, which integrates different signaling pathways, including neuromodulation, metabolism and proliferation, to control a vast number of physiological processes^2^. Monitoring the activity of specific kinases thus can provide important insights into cellular physiology, an example being the measurements of PKA activities as a readout for neuromodulation in the brain^3–13^. Currently, phosphorylation state-specific antibodies are widely used to analyze protein phosphorylation levels at a single time point in cell lysates and fixed samples. However, the inaccessibility of the antigen to the antibody can compromise the sensitivity and quantifiability of the approach^14^. Genetically encoded kinase activity reporters with fluorescence or luminescence readouts allow real-time monitoring of kinase activity ex vivo and in vivo^15^. While these sensors offer exquisite spatiotemporal resolution, the restricted field of view of optical approaches and the limited penetration of light into tissue poses some restrictions with respect to number of cells and tissue regions investigated. Furthermore, long-term in vivo imaging remains laborious and requires custom-built imaging equipment. A recent optogenetic approach, KINACT, relies on reporter gene expression that is induced both by kinase activity and illumination with blue light^16^. Converting transient kinase signals into a ‘permanent’ mark separates recording from analysis and thus facilitates the parallel investigation of large number of cells. However, the approach requires reporter gene expression for 1 day after illumination and still suffers from the same limitations as other optical approaches. Directly recording kinase activity with high temporal resolution in a scalable manner or in deep tissues without the need for illumination thus remains challenging.

To address these challenges and complement the aforementioned approaches, we developed split-HaloTag recorders for kinase activity-dependent protein labeling (Kinprola). Kinprola enables the accumulation of an irreversible label in the presence of both a specific kinase activity and a fluorescent HaloTag substrate. Kinprola allows scalable recording of kinase activities for later analysis in different cellular compartments, and its modular design enables the generation of Kinprola variants for different kinases. We demonstrate the versatility of Kinprola for applications in transcriptome analysis, in functional genomic screening and for the tracking of neuromodulation in freely moving mice.

## Results

### Development of Kinprola

**Fig. 1:**
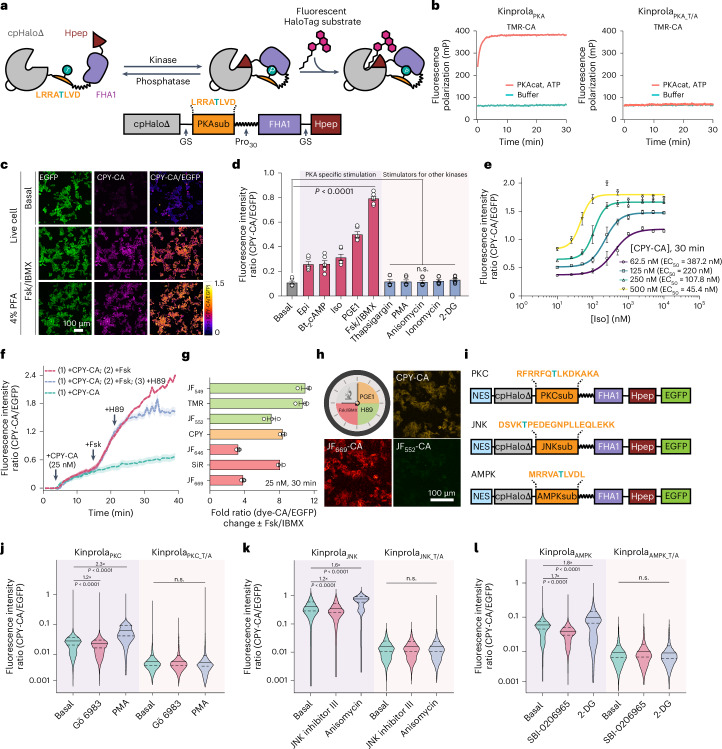
Design and characterization of the Kinprola recorder. **a**, A schematic of the Kinprola_PKA_ design. GS, Gly-Ser; Pro30, 30 Proline. **b**, The labeling kinetics of Kinprola proteins. **c**, The fluorescence images of HEK293 cells expressing Kinprola_PKA_ under different treatments. The representative images from four wells of cell culture show similar results. **d**,**e** A flow cytometry analysis of HEK293 cells expressing Kinprola_PKA_ treated with different stimulators (**d**) or varying Iso doses (**e**), apparent EC_50_ values from sigmoidal curve fitting. EC_50_, half maximal effective concentration of Iso. *P* < 0.0001 between basal and PKA-specific stimulation; *P* = 0.9867, 0.9999, >0.9999 between basal and 2-DG, Ionomycin or others. **f**, Representative time-lapse traces recorded from HEK293 cells expressing Kinprola_PKA_ successively treated with Fsk and H89. **g**, A flow cytometry analysis of HeLa cells expressing Kinprola_PKA_ labeled with different fluorescent substrates in the presence or absence of Fsk/IBMX. **h**, A recording of successive periods of PKA activity in HEK293 cells. **i**, The domain structures of Kinprola variants. **j**–**l**, Flow cytometry analysis of HeLa cells expressing different Kinprolas under various treatments. *P* < 0.0001 between Kinprolas basal and treatments; *P* > 0.9999 or *P* = 0.3779 between Kinprola_PKC___T/A_ basal and Gö 6983 or PMA; *P* > 0.9999 between Kinprola_JNK___T/A_, Kinprola_AMPK___T/A_ basal and treatments. Recording condition: 25 nM CPY-CA, 30 min for **c**, **d** and **j**–**l**. The error bars indicate the mean ± standard error of the mean for **d**–**g** or the median with the interquartile range for **j**–**l**, and data are from three technical replicates for **b**, three independent experiments with duplicates for **d**–**h** or triplicates for **j**–**l**. The statistical significance was calculated using a one-way ANOVA with Dunnett’s post hoc test for **d** and **j**–**l**. Scale bars, 100 μm for **c** and **h**. n.s., not significant. Source data

**Extended Data Fig. 1:**
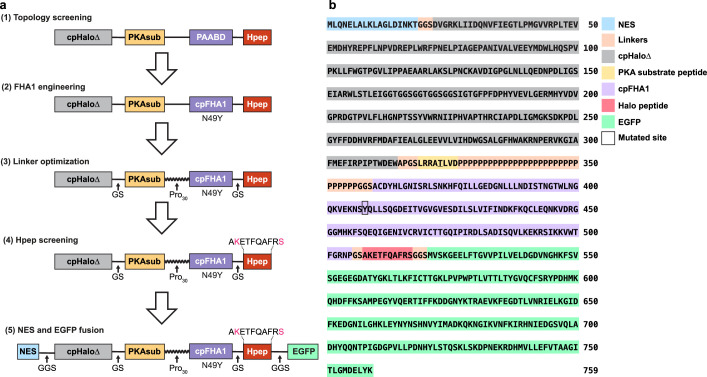
Strategy for designing, optimizing, and screening Kinprola_PKA_. (**a**) Flowchart of the Kinprola_PKA_ development process. PAABD: phosphoamino acid binding domain; PKAsub: PKA substrate peptide; Hpep: Halo peptide. (**b**) Amino acid sequence of Kinprola_PKA_, with the targeting sequence, domains, linkers, phosphorylation site, and mutated site annotated.

**Extended Data Fig. 2:**
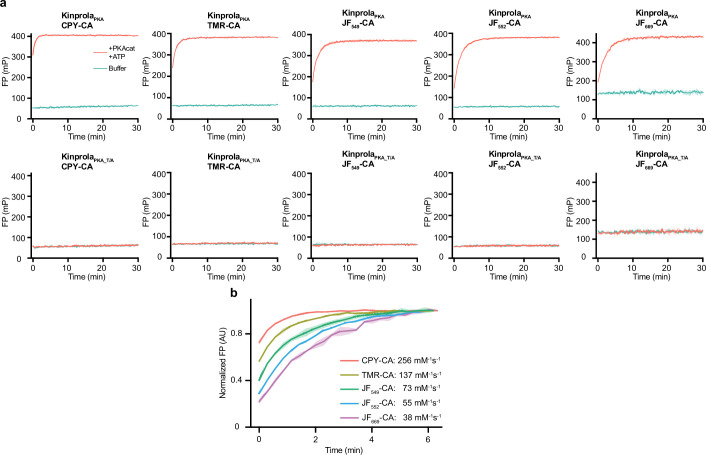
(related to Fig. 1) Labeling kinetics of Kinprola with different fluorescent HaloTag substrates. (**a**) Labeling kinetics of Kinprola_PKA_ and Kinprola_PKA_T/A_ (200 nM) with different fluorescent HaloTag substrates (50 nM) in the presence or absence of PKAcat (25 ng μL^−1^) and ATP (500 μM), measured by fluorescence polarization (FP). TMR-CA kinetics are shown in Fig. 1b. (**b**) Comparison of normalized labeling kinetics across substrates from (**a**), with fluorescence polarization values normalized to unbound and fully bound states. AU, arbitrary units. Data from three technical replicates. Source data

We first focused on the generation of a recorder for PKA, Kinprola_PKA_, in which reversible phosphorylation of Kinprola_PKA_ by PKA would activate its labeling activity. To develop Kinprola_PKA_, we utilized the split-HaloTag system recently developed by our group^17^. The system is comprised of a truncated, circularly permutated HaloTag (cpHaloΔ) that retains the overall fold of HaloTag but exhibits almost no activity and a decapeptide (Hpep) that can bind to cpHaloΔ and restore its activity toward chloroalkane (CA) substrates. We envisioned generating Kinprola_PKA_ by connecting cpHaloΔ and Hpep through a linker comprising the forkhead-associated domain 1 (FHA1) and a PKA-specific substrate peptide (PKAsub). Specific binding of FHA1 to phosphorylated PKAsub should then result in a conformational change of Kinprola_PKA_ that enables the activation of cpHaloΔ by binding to Hpep (Fig. 1a and Extended Data Fig. 1a). We used structural information on the FHA1–phosphothreonine peptide complex^18^ to design circularly permuted FHA1 variants and tested, together with wild-type FHA1, their performance in Kinprola_PKA_. We identified circularly permuted FHA1 variants with new C and N termini at positions 53 and 54, respectively, which showed a strong dependence of labeling rates on phosphorylation of Kinprola_PKA_. In addition, we incorporated the FHA1 mutation N49Y, which is known to enhance protein thermostability^19^ (Extended Data Fig. 1b and Supplementary Table 1). Finally, we systematically optimized the length and composition of the linker sequences and screened different Hpep variants. The final version of Kinprola_PKA_ showed no significant labeling with a fluorescent tetramethyl-rhodamine HaloTag substrate (TMR-CA) in its nonphosphorylated form but exhibited a more than thousandfold increase in labeling speed after being phosphorylated by PKA catalytic subunit (PKAcat) (that is, second-order rate constant after phosphorylation (*k*_TMR-CA_) = 1.37 × 10^5^ M^−1^ s^−1^) (Fig. 1b, Extended Data Fig. 2 and Supplementary Table 2). When mutating the phospho-acceptor site threonine to alanine (T/A) in the PKAsub, resulting in Kinprola_PKA_T/A_, no increase in labeling rate was observed after incubation with PKAcat and ATP (Fig. 1b and Extended Data Fig. 2). Kinprola_PKA_ was also rapidly labeled in a PKA-dependent manner by several other spectrally distinct fluorescent substrates (Extended Data Fig. 2, Supplementary Fig. 1 and Supplementary Table 2).

**Extended Data Fig. 3:**
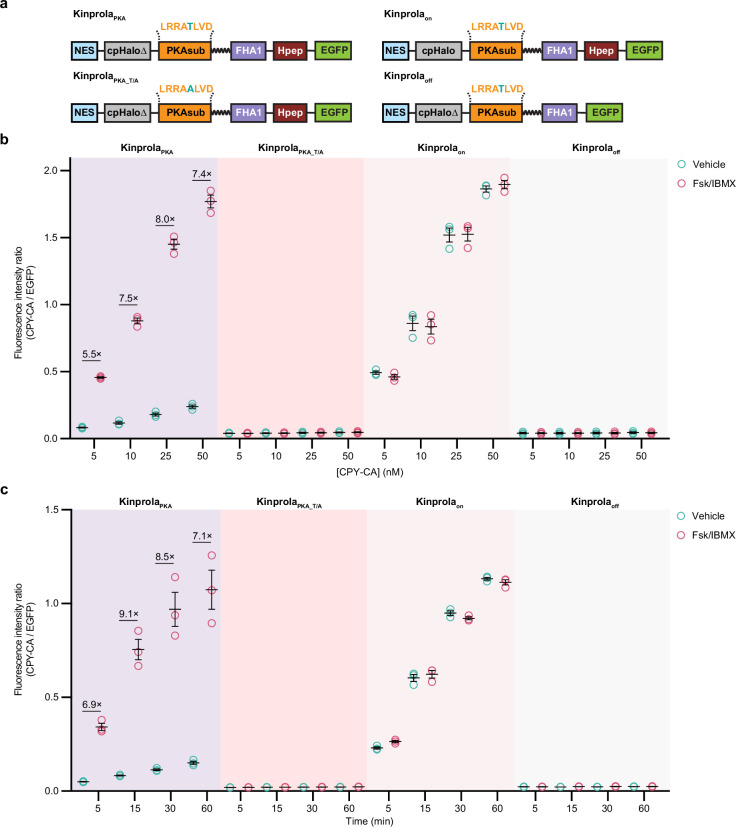
(related to Fig. 1) Time and fluorescent substrate-dependence of Kinprola labeling. (**a**) Domain structures of Kinprola_PKA_, Kinprola_PKA_T/A_, Kinprola_on_, and Kinprola_off_. (**b**) Flow cytometry analysis of HeLa cells expressing the indicated Kinprola variants labeled with varying concentrations of CPY-CA, with or without 50 μM Fsk/100 μM IBMX stimulation for 30 min. (**c**) Time-course flow cytometry analysis of Kinprola labeling with 25 nM CPY-CA under the same stimulation conditions as in (**b**). Error bars indicate mean ± SEM, data from three independent experiments with triplicates (**b**, **c**). Source data

**Extended Data Fig. 4:**
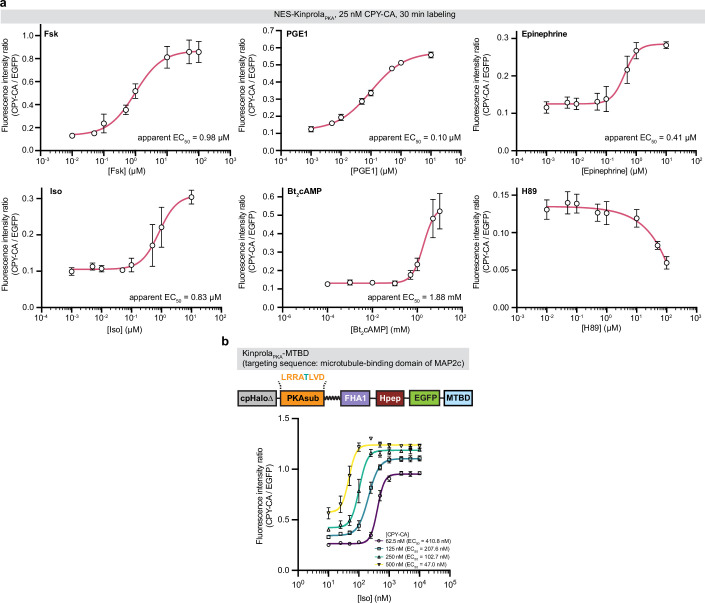
(related to Fig. 1) Kinprola_PKA_ labeling in response to varying concentrations of PKA modulators in HEK293 cells. (**a**) Flow cytometry analysis of HEK293 cells stably expressing cytoplasm-targeted Kinprola_PKA_ labeled with 25 nM CPY-CA for 30 min in the presence of varying concentrations of PKA modulators. (**b**) Flow cytometry analysis of HEK293 cells stably expressing microtubule-targeted Kinprola_PKA_ treated with increasing concentrations of Iso and CPY-CA for 30 min. Error bars indicate mean ± SEM, data from three (**a**) or two (**b**) independent experiments with replicates. Curves were fitted with the sigmoidal function to determine the apparent EC_50_ values. Source data

**Extended Data Fig. 5:**
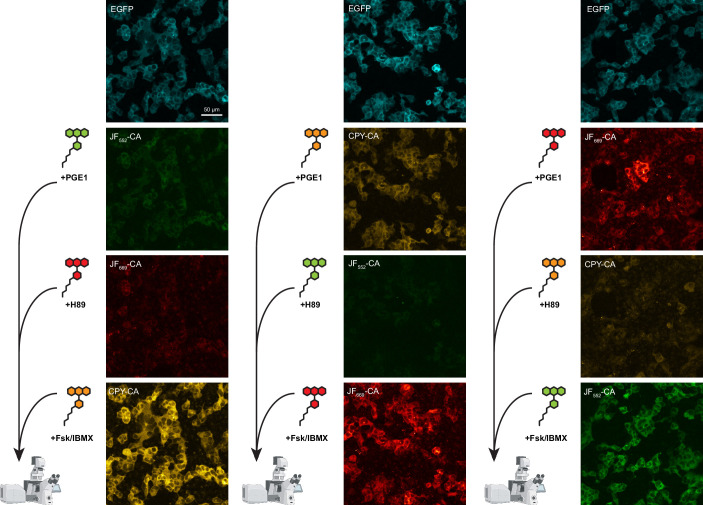
(related to Fig. 1) Sequential recording of PKA activity in Kinprola_PKA_-expressing HEK293 cells. Fluorescence images showing three successive recordings of PKA activity following: (1) PGE1 stimulation, (2) H89 inhibition, and (3) Fsk/IBMX stimulation, using JF_552_-CA or JF_669_-CA or CPY-CA. Cells rested for 2 h between treatments. Imaging was performed after the third incubation period. The extent of labeling is proportional to treatment strength: H89 < PGE1 < Fsk/IBMX, independent of the fluorescent substrate. Representative images from three independent experiments with similar results. Partial images shown in Fig. 1h. Created in BioRender. Sun, D. (2025) https://BioRender.com/6y47d5r. Scale bar: 50 μm.

To record cytosolic PKA activities in cultured mammalian cells, we fused a nuclear export signal sequence and enhanced green fluorescent protein (EGFP) to Kinprola_PKA_. EGFP was introduced to normalize the fluorescence signal of labeled Kinprola_PKA_ for differences in expression levels of the recorder. We also constructed constitutively active Kinprola_on_ by replacing cpHaloΔ with full length cpHalo and constitutively inactive Kinprola_off_ by deleting Hpep. Similar to Kinprola_PKA_T/A_, Kinprola_off_ only accumulated background labeling (Extended Data Fig. 3a). The HEK293 cells expressing Kinprola_PKA_ were incubated with a fluorescent carbopyronine HaloTag substrate (CPY-CA) in the presence or absence of the adenylyl cyclase activator forskolin (Fsk) and the pan-phosphodiesterase inhibitor 3-isobutyl-1-methylxanthine (IBMX), a stimulation that induces high PKA activity by raising cellular cAMP levels^12,20^. Incubation with Fsk/IBMX led to strong labeling, whereas very little labeling was observed in its absence. Furthermore, the fluorescent labeling resisted subsequent chemical fixation (Fig. 1c and Supplementary Fig. 2). A flow cytometry analysis revealed an eightfold change in normalized fluorescence intensity (CPY-CA/EGFP) between Fsk/IBMX-treated and untreated cells (Fig. 1d and Supplementary Fig. 3). Labeling times as short as 5 min and CPY-CA concentrations as low as 5 nM were sufficient to detect a difference between Fsk/IBMX-treated and untreated cells (Extended Data Fig. 3). By contrast, Kinprola_on_ showed negligible differences with or without Fsk/IBMX treatment, and neither Kinprola_off_ nor Kinprola_PKA_T/A_ showed significant labeling (Extended Data Fig. 3). Kinprola_PKA_ labeling was dose dependent on Fsk, and directly raising intracellular cAMP concentrations with the cell-permeable cAMP analog (Bt_2_cAMP) also increased Kinprola_PKA_ labeling (Fig. 1d and Extended Data Fig. 4a). The cAMP/PKA pathway acts as a central downstream mediator of multiple G-protein-coupled receptor (GPCR) signaling pathways and Kinprola_PKA_ also strongly responded to several key Gα_s_-coupled receptor agonists such as epinephrine, isoproterenol (Iso) and prostaglandin E1 (PGE1) in a dose-dependent manner (Fig. 1d,e and Extended Data Fig. 4a). Furthermore, incubation with the PKA inhibitor H89 further suppressed the already low basal labeling of Kinprola_PKA_ (Extended Data Fig. 4a). Various stimulators of kinases other than PKA did not increase Kinprola_PKA_ labeling, confirming that Kinprola_PKA_ specifically reports on PKA activation (Fig. 1d). Using different concentrations of CPY-CA, the responsiveness of Kinprola_PKA_ to varying doses of Iso stimulation can be finely tuned, both for Kinprola_PKA_ with cytosolic localization as well as microtubule targeting (Fig. 1e and Extended Data Fig. 4b). While Kinprola_PKA_ was designed to record periods of PKA activity for later analysis, it can also be used for real-time recording. Addition of Fsk to HEK293 cells expressing Kinprola_PKA_ resulted in an increase in Kinprola_PKA_ labeling as measured by timelapse fluorescence microscopy. In these experiments, the rate of Fsk-induced labeling can be modulated by varying the concentration of CPY-CA (Supplementary Fig. 4) and attenuated to the basal labeling level by the addition of the PKA inhibitor H89 (Fig. 1f). This demonstrates that Kinprola_PKA_ responds to sudden changes in cellular PKA activity. Furthermore, a variety of different fluorescent HaloTag substrates can be used for the Fsk/IBMX-dependent fluorescent marking of Kinprola_PKA_-expressing cells (Fig. 1g and Supplementary Fig. 1), and we leveraged this to distinguish multiple recording periods in a single cell. Specifically, the cells expressing Kinprola_PKA_ were first incubated with CPY-CA and PGE1 (moderate stimulation of PKA), then with the fluorescent Janelia Fluor (JF) 552 HaloTag substrate (JF_552_-CA)^21^ in the presence of H89 (inhibition of PKA) and finally with JF_669_-CA^22^ and Fsk/IBMX (strong stimulation of PKA). Post hoc imaging showed the expected pattern of moderate labeling with CPY, weak labeling with JF_552_ and strong labeling with JF_669_ (Fig. 1h). By switching the combination of fluorescent substrates and drugs, the differences in labeling efficiencies for the three periods were observed to be independent of which fluorescent substrate was used for which recording period (Extended Data Fig. 5).

**Extended Data Fig. 6:**
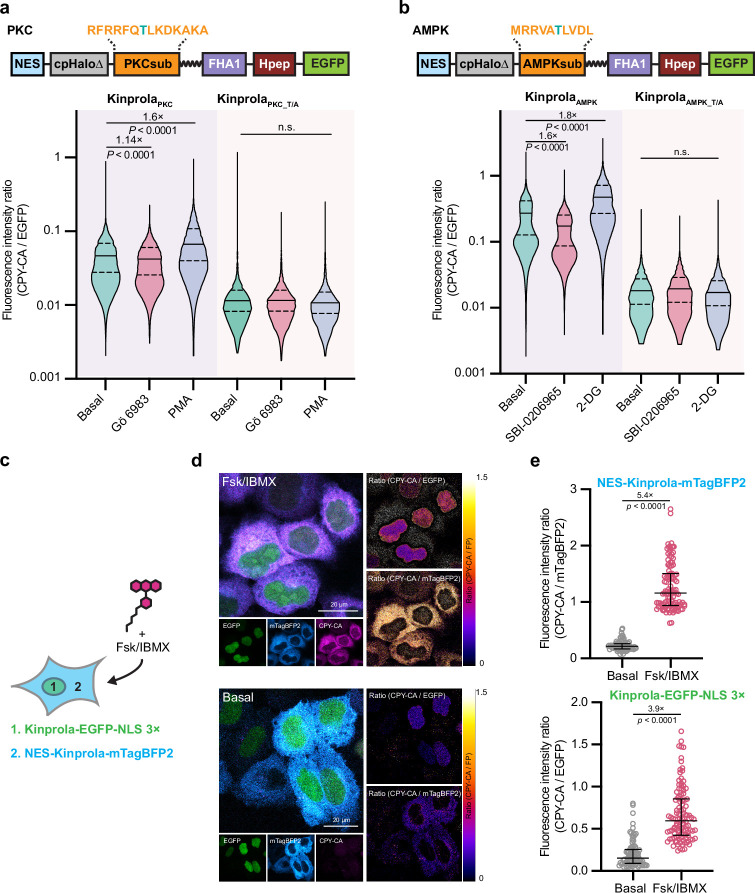
(related to Fig. 1) Kinprola for recording diverse kinase activities and subcellular PKA signaling. Flow cytometry analysis of Kinprola labeling with 25 nM CPY-CA for 30 min in HEK293 cells treated with corresponding kinase activators or inhibitors. Data from three independent experiments with triplicates. *p* < 0.0001 between Kinprola basal and treatments; *p* = 0.3472, 0.5634 between Kinprola_PKC_T/A_ basal and Gö 6983 or PMA; *p* > 0.9999 between Kinprola_AMPK_T/A_ basal and treatments. (**c**) Schematic of simultaneous Kinprola_PKA_ recording in nuclear and cytoplasmic compartments. (**d**) Fluorescence images of HeLa cells expressing nuclear and cytoplasmic Kinprola_PKA_, with or without Fsk/IBMX stimulation in the presence of 50 nM CPY-CA for 30 min. Representative images from three independent experiments with similar results. Scale bars: 20 μm. (**e**) Dot plot of normalized fluorescence intensities (CPY-CA/mTagBFP2) and (CPY-CA/EGFP) from (**d**), data collected from three independent experiments. *p* < 0.0001 between basal and Fsk/IBMX group. Error bars indicate median with interquartile range (**a**, **b**, **e**). Statistical significance was calculated with one-way ANOVA with Dunnett’s Post hoc test (**a**, **b**) or unpaired two-tailed Welch’s *t* test (**e**). Source data

Kinprolas for other kinases can be created by substituting the PKAsub with substrate peptides specific for other kinases such as protein kinase C (PKC)^23^, c-Jun N-terminal kinases (JNKs)^24^ and AMP-activated protein kinase (AMPK)^25^. Without additional engineering, these Kinprola variants recorded the activities of their cognate kinases during drug stimulation or inhibition in the cytosol of mammalian cells, with minimal responses observed in T/A mutant negative controls (Fig. 1i–l and Extended Data Fig. 6a,b). Furthermore, Kinprola_PKA_ can be (simultaneously) used in different cellular compartments. For this, Kinprola_PKA_ variants with different localization tags were simultaneously expressed in cytoplasm and nucleus of single cells. Different fluorescent proteins were introduced to normalize the labeling intensity separately (Extended Data Fig. 6c). Fsk/IBMX stimulation, in the presence of CPY-CA, resulted in increased fluorescent labeling both in the nucleus and the cytosol compared with fluorescent labeling observed in the absence of Fsk/IBMX (Extended Data Fig. 6d,e).

### Kinprola_PKA_ recording for transcriptome analysis

**Fig. 2:**
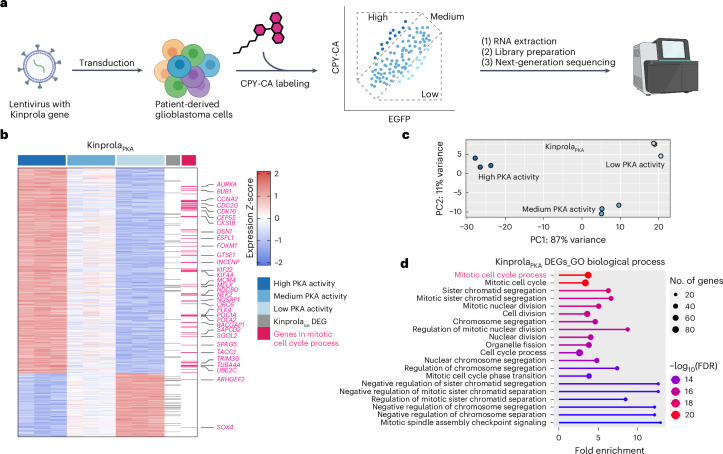
Kinprola enables selection of cell subpopulations based on PKA activity for transcriptome analysis. **a**, A schematic of Kinprola_PKA_ recording in GBCs for subsequent bulk RNA-seq. Created with BioRender.com. **b**, The transcriptional profiles of three sorted Kinprola_PKA_-expressing GBC groups. The DEGs are color-coded by *Z*-score. The genes in the GO term ‘mitotic cell cycle process’ are highlighted in magenta, and the representative gene symbols are listed. The overlapped DEGs in both the Kinprola_PKA_ and Kinprola_on_ groups are in gray. RNA-seq is from biological triplicates. **c**, The principal component analysis of the three sorted Kinprola_PKA_-expressing GBC groups. PC1 (x axis) and PC2 (y axis) correspond to the first and second principal components. **d**, The top 20 GO biological processes enriched in Kinprola_PKA_-identified DEGs, ranked by a FDR. n.s., not significant. Source data

Recording transient kinase activity for later analysis is particularly valuable for correlating cellular phenotypes with kinase signaling in large and heterogeneous cell populations. An example of a heterogeneous cell combination is glioblastoma, the most frequent and aggressive adult-type diffuse glioma^26,27^. Glioblastoma cells (GBCs) exhibit diverse phenotypic and behavioral characteristics. For instance, the formation of tumor microtubes diversifies GBC invasiveness, which also positively correlates with their proliferation^28–31^. Recently, using Caprola_6_, a split-HaloTag recorder for cytosolic calcium transients, we observed heterogeneous calcium signaling signatures in GBC subpopulations^17,31^. To investigate whether GBC subpopulations selected using Kinprola_PKA_ with varying PKA activities and the potential connection with calcium signaling, we expressed Kinprola_PKA_ in patient-derived GBCs and cultured them in a two-dimensional monoculture under serum-free stem-like conditions, in which GBCs retain their capacity for tumor microtubes formation^31,32^. Following labeling with CPY-CA, the GBCs were sorted into high, medium and low normalized labeling intensity groups using fluorescence-activated cell sorting (FACS) and subjected to bulk RNA sequencing (RNA-seq) (Fig. 2a and Supplementary Fig. 5a). As a control, GBCs expressing Kinprola_on_ underwent the same procedure to eliminate potential labeling differences due to CPY-CA permeability heterogeneity (Supplementary Fig. 5b).

**Extended Data Fig. 7:**
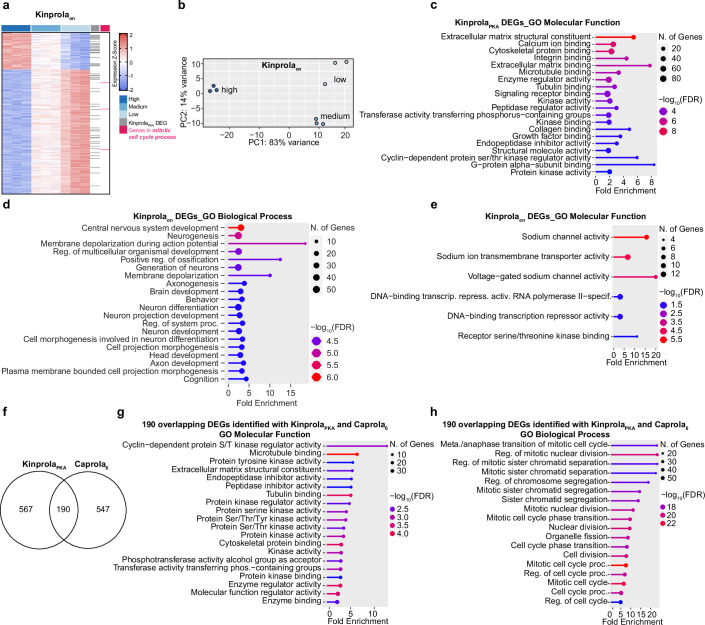
(related to Fig. 2) Transcriptome analysis of GBC subpopulations selected by Kinprola labeling. (**a**) Heatmap of DEGs from three Kinprola_on_-labeled GBC subpopulations, color-coded by Z-score. Genes in the GO term ‘mitotic cell cycle process’ are in magenta; DEGs shared with Kinprola_PKA_ are in gray. (**b**) First principal coordinate analysis of the three Kinprola_on_-expressing groups. (**c**–**e**) GO term enrichment for Kinprola_PKA_ (**c**) and Kinprola_on_ (**d**, **e**) DEGs, ranked by FDR. (**f**) Venn diagram showing overlap between Kinprola_PKA_- and Caprola_6_-identified DEGs. (**g**, **h**) GO terms for overlapping DEGs from (**f**), ranked by FDR. Source data

The transcriptomic profiles of three groups were clearly separated in Kinprola_PKA_, and 737 differentially expressed genes (DEGs) between all groups in pairwise group analysis were identified (Fig. 2b,c). Among the 737 Kinprola_PKA_-identified DEGs, only 55 DEGs were overlapped with 326 DEGs identified by Kinprola_on_ (Extended Data Fig. 7a,b), suggesting their unique signatures. Subsequent Gene Ontology (GO) analysis, conducted without the coinciding DEGs, revealed that GO terms associated with proliferation, such as mitotic cell cycle process and extracellular matrix were over represented in the 682 Kinprola_PKA_-specific DEGs (Fig. 2d and Extended Data Fig. 7c). By contrast, the molecular function of DEGs identified by Kinprola_on_ was associated with transporters and other features (Extended Data Fig. 7d,e). This observation is consistent with previous studies on PKA activity oscillations during the cell mitotic process^33–35^, suggesting the impact of PKA activity for GBC proliferation. Interestingly, 190 Kinprola_PKA_ DEGs overlapped with those identified by Caprola_6_^17^, while the remaining three-quarters are unique to their respective signatures (Extended Data Fig. 7f). This demonstrates the specificity of Kinprola_PKA_ and Caprola_6_ to select GBC subpopulations on the basis of PKA activities and calcium transients. GO analysis of these overlapping DEGs revealed enriched features including kinase activity and mitotic cell cycle process (Extended Data Fig. 7g,h), indicating that both PKA and cytosolic calcium play important roles in regulating these overlapping genes during the GBC mitotic process. Taking together, these experiments demonstrate the capability of Kinprola for stably marking, selecting and analyzing cell subpopulations within heterogeneous networks in a high-throughput and scalable manner.

### Kinprola_PKA_ recording for CRISPR-knockout screening

**Fig. 3:**
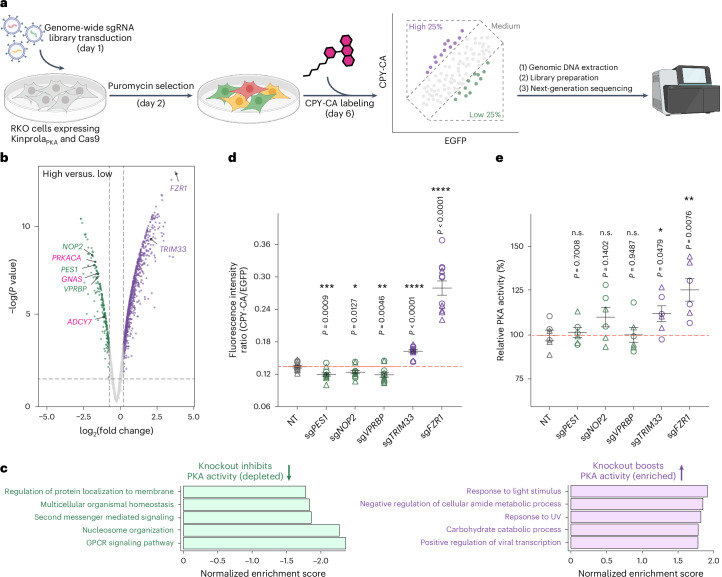
Kinprola enables identification of PKA signaling regulators through CRISPR-knockout screening. **a**, A schematic of Kinprola_PKA_ recording in RKO cells for pooled CRISPR-knockout screening. Created with BioRender.com. **b**, A volcano plot of gene enrichment comparing ‘high’ versus ‘low’ Kinprola_PKA_-labeled populations. The dashed lines indicate the FDR threshold (0.05). Putative hits and canonical regulators (*PRKACA*, *GNAS* and *ADCY7*) are highlighted. The data are from two independent screens. The statistical significance was calculated by fitting a linear model for each gene, and the resulting *P* value was corrected for multiple testing using a Benjamini–Hochberg correction. **c**, GSEA of the top five categories from the ‘high’ versus ‘low’ comparison. **d**, A dot plot of normalized Kinprola_PKA_ labeling (CPY-CA/EGFP) in cells with individual knockouts of putative regulators. The NT group represents the NT control sgRNAs. **e**, A validation of putative regulators by ELISA-based PKA activity assay in cell lysates. The PKA activity was normalized to the NT group. The circles and triangles represent two distinct sgRNAs per gene for **d** and **e**. The error bars indicate the mean ± standard error of the mean for **d** and **e**, and the data are from six (for **d**) or three (for **e**) independent experiments with duplicates. The same RKO cell line was used in **b**–**e**. The statistical significance was calculated with unpaired two-tailed Welch’s *t-*test. The *P* values are shown for **d** and **e**. n.s., not significant; UV, ultraviolet. Source data

**Extended Data Fig. 8:**
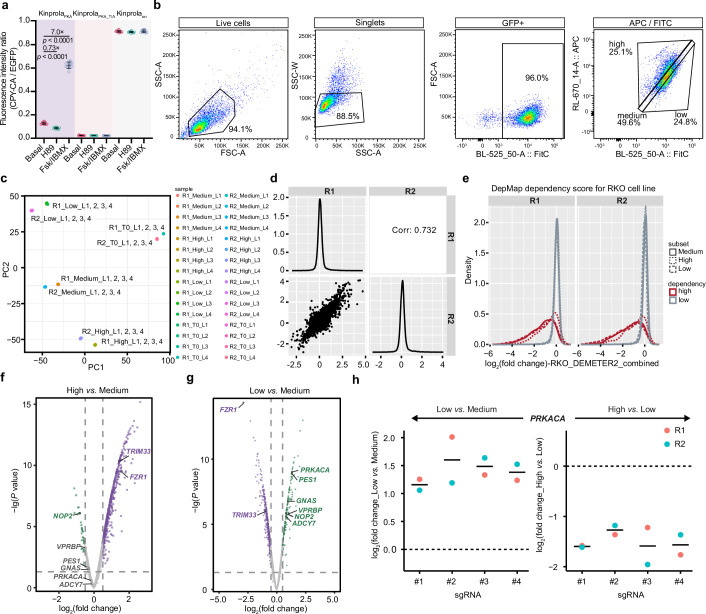
(related to Fig. 3) CRISPR knockout screen of RKO cells sorted by Kinprola_PKA_ labeling. (**a**) Flow cytometry analysis of RKO cells stably expressing Kinprolas under different treatments (125 nM CPY-CA, 30 min). Statistical analysis by unpaired two-tailed Welch’s *t* test; *p* < 0.0001 between basal and treatments. Error bars, mean ± SD from three independent experiments with triplicates. (**b**) Gating and sorting strategy of CPY-CA-labeled cells for screening. (**c**) Principal component analysis of sorted populations from two independent screens. (**d**) Gene-level reproducibility between two independent screens. (**e**) Core-essential genes (DepMap) were significantly depleted relative to nonessential genes in CRISPR screen. (**f**, **g**) Volcano plots of gene enrichment in ‘high’ *vs*. ‘medium’ and ‘low’ *vs*. ‘medium’ populations; FDR 0.05 cutoff shown. Putative hits and canonical regulators (*PRKACA*, *GNAS*, *ADCY7*) highlighted. Statistical significance was calculated by fitting a linear model for each gene, and the resulting *p* values were corrected for multiple testing using Benjamini-Hochberg correction. (**h**) Enrichment of four *PRKACA*-targeting sgRNAs in sorted groups. All enriched in ‘low’ and depleted in ‘high’ groups across two independent screens, with central lines indicating the average. Source data

To demonstrate the capability of Kinprola_PKA_ for identifying potential regulators of PKA, we combined a pooled CRISPR-knockout screening approach with Kinprola_PKA_, which can select cells on the basis of their relative PKA activities after genetic perturbation. First, transgenes for Kinprola_PKA_ and Cas9 were introduced into RKO colon cancer cells by lentiviral integration. The cells stably expressing the two transgenes exhibited robust responses to both drug stimulation and inhibition compared with cells expressing Kinprola_on_ and the T/A mutant (Extended Data Fig. 8a). To conduct pooled genetic perturbation, the stable cells were transduced with a lentiviral genome-wide single guide RNA (sgRNA) library^36^ at a multiplicity of infection (MOI) of 0.2 to 0.3, followed by puromycin selection of transduced cells, expansion and labeling with CPY-CA at day 6 after transduction. The labeled cells were then sorted into three populations on the basis of their normalized labeling intensity (high 25%, lowest 25% and medium) (Fig. 3a and Extended Data Fig. 8b). Next-generation sequencing was performed on each sorted sample to determine the abundance of each sgRNA present in the three cell subpopulations. By comparing the sgRNA representation in each subpopulation, the sgRNAs that significantly altered Kinprola_PKA_ labeling were inferred. The screen was independently performed twice to identify differentially regulated gene targets (Extended Data Fig. 8c–e). Of the 18,659 protein-coding genes targeted by the sgRNA library, a total of 340 hits from the ‘high’ versus ‘low’ comparison were selected with a false discovery rate (FDR) threshold of 0.05 (Fig. 3b). A total of 45 genes were identified that decreased normalized labeling intensity, which represent knockouts that decreased PKA activity, whereas 295 genes were identified that increased PKA activity after knockout. Notably, canonical regulators in the GPCR-cAMP-PKA signaling pathway, including the catalytic subunit α of PKA (*PRKACA*), the heterotrimeric G protein Gα_s_ subunit (*GNAS*) and the adenylyl cyclase 7 (*ADCY7*), were among the identified hits for which knockout decreased PKA activity. These three regulators were also found to be enriched in the ‘low’ versus ‘medium’ comparison but not in the ‘high’ versus ‘medium’ comparison (Fig. 3b and Extended Data Fig. 8f–h). A gene set enrichment analysis (GSEA) was subsequently performed to identify relevant biological processes that were enriched among the hits. The GO terms related to stress response and metabolic process were over represented in hits for which knockout increased PKA activity, whereas the terms associated with GPCR signaling pathways, second messenger-mediated signaling and nucleosome organization were over represented in collections of genes for which knockout decreases PKA activity (Fig. 3c and Supplementary Fig. 6).

We then examined three genes for which knockout decreased PKA activity, that is, *PES1*, *NOP2* and *VPRBP*, and two genes for which knockout increased PKA activity, that is, *TRIM33* and *FZR1*. For each of the five genes, we used two individual sgRNAs for knockouts and examined their effect on PKA activity as measured by Kinprola_PKA_ in the cell line used in the CRISPR screen. Consistent with the results from the CRISPR screen, knockout of *PES1*, *NOP2* and *VPRBP* decreased PKA activity, and knockout of *TRIM33* and *FZR1* increased PKA activity as compared with nontargeting (NT) sgRNAs (Fig. 3d). Furthermore, we attempted to measure PKA activity in cell lysates of these five knockout lines. Using an enzyme-linked immunosorbent assay (ELISA)-based PKA colorimetric activity assay, we were able to detect a significant increase in PKA activity in lysates of cells in which *FZR1* was knocked out. For the four other knockouts, only subtle or nonsignificant changes were observed in cell lysates (Fig. 3e and Supplementary Fig. 7). This can be attributed to the moderate editing efficacy (Supplementary Figs. 8 and 9), low sensitivity of the underlying assay and the nonphysiological conditions during the measurement. *FZR1* encodes cdh1, an important component of the anaphase promoting complex/cyclosome (APC/C), which controls cell cycle fate decisions^37^. Knockout of *FZR1* leads to unscheduled cell cycle progression, subsequently triggering replicative stress and DNA damage responses^38^. In addition, previous studies showed that PKA activity oscillates throughout the cell cycle and negatively regulates APC/C by phosphorylating several of its components to ensure proper activation^33,35^. Our screen results thus indicate a potential regulatory connection between *FZR1* function and PKA activity. Overall, our CRISPR screen highlights the influence of diverse biological processes on the regulation of PKA signaling and outlines how Kinprola can be used in genetic screens to unravel genes regulating kinase activity.

### Recording PKA activation in neurons, brain slices and in vivo

**Fig. 4:**
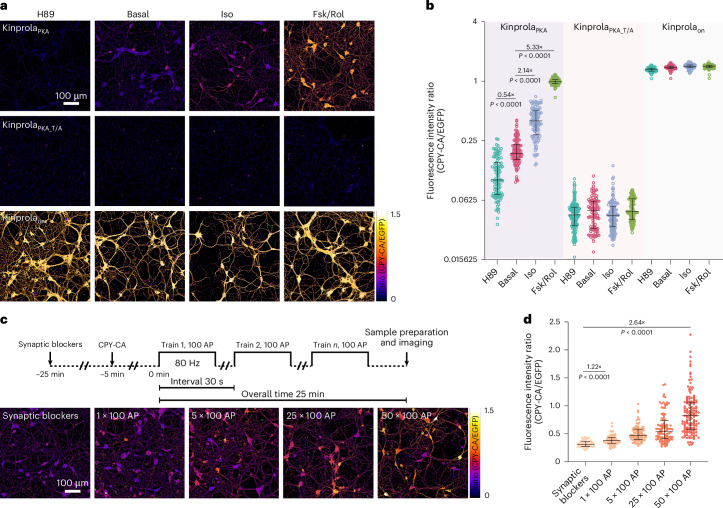
Kinprola enables rapid recording of PKA activation in cultured neurons. **a**, The ratiometric fluorescence images (CPY-CA/EGFP) of primary rat hippocampal neurons expressing Kinprola_PKA_, Kinprola_PKA_T/A_ and Kinprola_on_ labeled with CPY-CA (25 nM, 45 min) following treatment with H89, Iso, Fsk/Rol or vehicle. **b**, A dot plot of normalized fluorescence intensity from **a** (*n* = 102, 104, 104, 102, 142, 96, 123, 106, 109, 103, 92 and 91 neurons). **c**, Ratiometric fluorescence images of neurons expressing Kinprola_PKA_ labeled with 125 nM CPY-CA following defined electrical field stimulation. AP, action potential. **d**, The dot plots of normalized fluorescence intensity from **c** (*n* = 51, 65, 92, 100 and 138 neurons). The error bars indicate the median with the interquartile range, and the data were collected from six fields of view across two cultures in one independent experiment for **b** and **d**. The statistical significance was calculated with an unpaired two-tailed Welch’s *t*-test for **b** and **d**, *P* < 0.0001 between treatments and basal or synaptic blockers. The representative images are from three independent experiments with similar results. Scale bars, 100 μm for **a** and **c**. Source data

**Extended Data Fig. 9:**
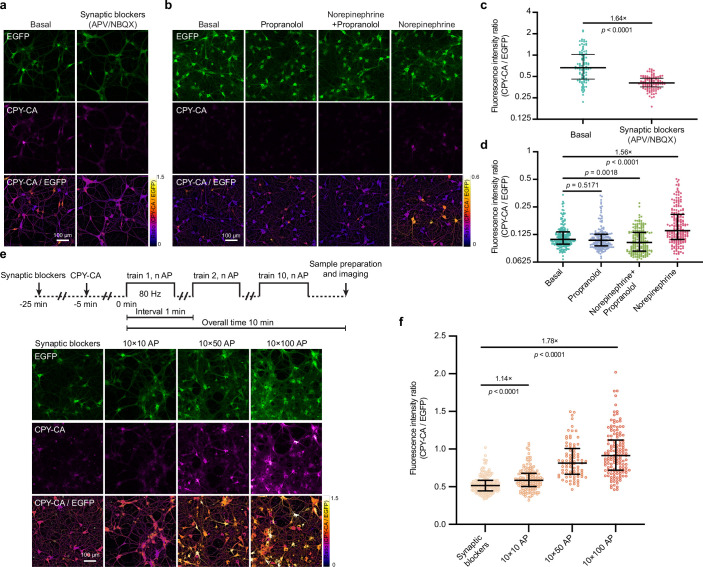
(related to Fig. 4) Kinprola_PKA_ records PKA activity in neurons under pharmacological and electrophysiological treatment. (**a**) Fluorescence images of primary rat hippocampal neurons expressing Kinprola_PKA_ labeled with CPY-CA (125 nM, 45 min) in the presence of synaptic blockers (APV/NBQX) or vehicle. Representative images from three independent experiments with duplicates. (**b**) Fluorescence images of neurons expressing Kinprola_PKA_ labeled with CPY-CA (25 nM, 45 min) treated with norepinephrine, propranolol, norepinephrine/propranolol or vehicle. Representative images from two independent experiments with duplicates. (**c**) Quantification of labeling in (**a**). n = 101 and 108 neurons, respectively. *p* < 0.0001 between basal and synaptic blockers group. (**d**) Quantification of labeling in (**b**). n = 183, 194, 168 and 185 neurons, respectively. *p* = 0.5171, 0.0018 and < 0.0001 between basal and treatments. (**e**) Fluorescence images of neurons expressing Kinprola_PKA_ labeled with 125 nM CPY-CA upon defined electrical field stimulation. Representative images from three independent experiments with duplicates. (**f**) Quantification of labeling in (**e**). n = 170, 146, 84 and 140 neurons, respectively. *p* < 0.0001 between synaptic blockers and 10×10 AP or 10×100 AP. Error bars indicate median with interquartile range; data collected from six fields of view across two cultures in one independent experiment, and statistical significance was calculated with unpaired two-tailed Welch’s *t* test (**c**, **d**, **f**). Scale bars: 100 μm (**a**, **b**, **e**). Source data

PKA integrates multiple GPCR signaling pathways and plays a key role in neuronal excitability and plasticity. Consequently, monitoring cellular PKA activity provides a valuable readout for neuromodulatory events^3–13^. To investigate if Kinprola_PKA_ can be used to track PKA activity changes in the nervous system, we first expressed Kinprola in cultured primary rat hippocampal neurons. Compared with Kinprola_on_ and the T/A mutant, the neurons expressing Kinprola_PKA_ exhibited a robust increase in labeling relative to basally active neurons when stimulated with Fsk/Rolipram (Rol) or Iso (Fig. 4a,b). By increasing the concentration of CPY-CA, Kinprola_PKA_ effectively captured transient PKA activity changes stimulated by low doses of Iso (Supplementary Fig. 10). Conversely, PKA inhibition by H89 or synaptic transmission silencing between neurons with glutamate receptor antagonists NBQX/APV resulted in decreased labeling compared with basally active neurons (Extended Data Fig. 9a,c). Moreover, the neurons expressing Kinprola_PKA_ responded to stimulation by the neuromodulator norepinephrine, with the response effectively blocked by cotreatment with the β-adrenergic receptor antagonist propranolol (Extended Data Fig. 9b,d). In NBQX/APV-silenced neurons, Kinprola_PKA_ effectively recorded the increase in cytosolic PKA activity elicited by electrically evoked action potentials (Fig. 4c,d and Extended Data Fig. 9e,f). Furthermore, the fluorescent Kinprola_PKA_ labeling signal in live neurons remained detectable for at least 3 days after recording (Supplementary Fig. 11).

**Fig. 5:**
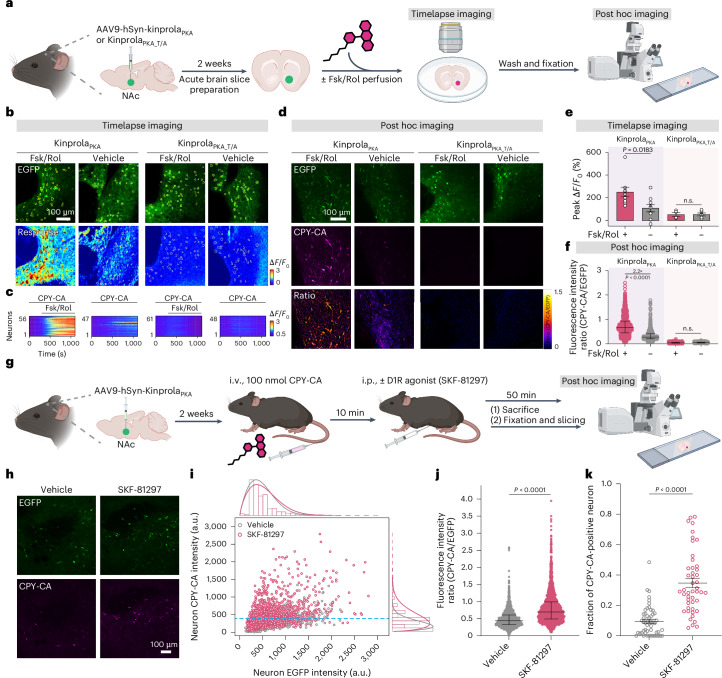
Kinprola records neuromodulation-induced PKA activation in brain slices and freely moving mice. **a**, A schematic illustrating Kinprola recording of pharmacological PKA activation in the NAc of acute mouse brain slices. **b**, Timelapse imaging of brain slices expressing Kinprola_PKA_ or Kinprola_PKA_T/A_ during Fsk/Rol or vehicle treatment. CPY-CA (250 nM) was perfused before and during stimulation. The response of each region of interest (ROI) was calculated using the equation Δ*F*/*F*_0_ = (*F* − *F*_0_) / *F*_0_, where *F*_0_ is defined as the average baseline fluorescence intensity; *F* is defined as the fluorescence intensity at each acquisition time point during perfusion. **c**, The fluorescence traces of individual neurons from **b**. **d**, The fixed-slice images from the timelapse experiment, representative images from three independent experiments with similar results. **e**, A quantification of peak CPY-CA signal changes (*n* = 9/5, 9/5, 4/3 and 6/3 slices/mice, respectively). *P* = 0.0183, >0.9999 between vehicle and Fsk/Rol treatment in Kinprola_PKA_ and Kinprola_PKA_T/A_ groups. **f**, A dot plot of CPY-CA/EGFP ratios for individual neurons from **d** (*n* = 1045, 1294, 438 and 614, respectively). *P* < 0.0001, *P* = 0.9980 between vehicle and Fsk/Rol treatment in Kinprola_PKA_ and Kinprola_PKA_T/A_ groups. **g**, A schematic illustrating in vivo Kinprola recording after SKF-81297 treatment. **h**, Representative images of CPY-CA-labeled NAc neurons after SKF-81297 or vehicle injection (four mice per group from two independent experiments and similar results from the other three mice shown in Supplementary Fig. 12a). **i**, A scatter plot of CPY-CA versus EGFP fluorescence (*n* = 2512 neurons/47 slices/4 mice/SKF-81297 group; 2593/52/4/vehicle group). The horizontal dashed line indicates the 90th percentile threshold of CPY-CA fluorescence in vehicle-treated neurons. **j**, A dot plot of the CPY-CA/EGFP signal of individual neurons (same neuron numbers as in **i**). **k**, The fraction of EGFP-positive neurons with CPY-CA above vehicle 90th percentile threshold (same slice numbers as in **i**). The error bars indicate the mean ± standard error of the mean for **e** and **k** or the median with interquartile range for **f** and **j**. The statistical significance was calculated with a one-way ANOVA with a Tukey’s post hoc test for **e** and **f** or unpaired two-tailed Welch’s *t*-test for **j** and **k**; *P* < 0.0001 between vehicle and SKF-81297 groups for **j** and **k**. Panels **a** and **g** were created with BioRender.com. Scale bars, 100 μm for **b**, **d** and **h**. i.v., intravenous; i.p., intraperitoneal; n.s., not significant. Source data

Next, we characterized the performance of Kinprola_PKA_ in acute mouse brain slices (Fig. 5a). Adeno-associated viruses (AAVs) with transgenes of Kinprola_PKA_ or its T/A mutant were stereotactically injected into bilateral nucleus accumbens (NAc) of mice, a striatal region that receives extensive dopaminergic input. A total of 2 weeks post injection, the acute brain slices were prepared, perfused with Fsk/Rol to evoke PKA activity and Kinprola_PKA_ labeling was followed through timelapse fluorescence microscopy. Rapid fluorescence elevation in CPY-CA channel was observed in Fsk/Rol-perfused slices expressing Kinprola_PKA_, whereas the fluorescence changes were significantly smaller in basally active slices (Fig. 5b,c,e). By contrast, no obvious response was detected in slices expressing the T/A mutant whether perfused with Fsk/Rol or vehicle (Fig. 5b,c,e). These experiments demonstrate rapid and specific Kinprola_PKA_ labeling upon PKA activation in a complex and near-native context. After real-time recording, free CPY-CA was washed out, and the slices were fixed and mounted. The distinct labeling differences between Fsk/Rol-perfused and vehicle-perfused slices were well preserved in the post hoc imaging and even enhanced relative to those observed in live-cell imaging as washing out free CPY-CA reduced background fluorescence (Fig. 5d,f).

Finally, we applied Kinprola_PKA_ to record neuromodulation-induced PKA activity changes in the NAc of freely moving mice. Dopaminergic signaling through abundant type 1 dopamine receptors (D1R) in the NAc can activate PKA and subsequently modulate a multitude of brain functions, including reward signaling and reinforcement learning^3,10,11,39^. To assess whether Kinprola_PKA_ can record PKA activity in response to D1R activation, mice expressing Kinprola_PKA_ first received an intravenous tail vein injection of CPY-CA. After 10 min, SKF-81297, a potent and selective D1/D5R agonist known to increase signaling via the cAMP/PKA pathway^8,10,11,40,41^ was administered via intraperitoneal (IP) injection. 50 min later, mice were sacrificed and NAc slices were processed for post hoc imaging (Fig. 5g). The normalized fluorescence intensity of single neurons expressing Kinprola_PKA_ significantly increased following SKF-81297 injection compared with the fluorescence labeling observed after vehicle injection (Fig. 5h–k and Supplementary Fig. 12). In SKF-81297-injected mice, around 35% neurons expressing Kinprola_PKA_ per slices in the NAc exhibited strong CPY-CA labeling, compared with around 10% after injection of vehicle (Fig. 5k). These experiments demonstrate the ability of Kinprola_PKA_ to directly and rapidly record PKA activity for monitoring neuromodulation with cellular resolution in vivo for later analysis in deep tissues of freely moving mice, providing a scalable and complementary strategy to current real-time PKA activity biosensors.

## Discussion

Genetically encoded fluorescent kinase activity reporters have been extensively utilized to monitor kinase activities in real time for elucidating the connections between intracellular signaling cascades and neuromodulation in the nervous system. However, such reporters face limitations when challenged to record kinase activities with high spatiotemporal resolution in a scalable manner or in deep tissues of freely moving animals. These limitations are mainly due to the inherent constraints of light microscopy. A strategy that potentially addresses these limitations would involve separating the recording period from its analysis by converting transient protein kinase activities into a ‘permanent’ mark for later analysis. To achieve this, we developed Kinprola, a chemigenetic approach for recording protein kinase activity based on our recently reported split-HaloTag system^17^. Kinprola accumulates an irreversible fluorescent mark in the presence of both a specific protein kinase activity and a fluorescent substrate. Thus, the light delivery required for monitoring the activity with fluorescent biosensors is replaced by the delivery of a fluorescent substrate. The recording window is time-gated by applying or washing out the fluorescence substrate, typically spanning from a few minutes to hours. Kinprola rapidly responds to the cellular phosphorylation state, enabling successive recordings of kinase activity during different periods through the use of spectrally distinct substrates. Kinprola is applicable across various systems, including cultured cell lines, primary neurons, brain slices and in vivo.

The design principle of Kinprola was inspired by the classical kinase activity reporters, in which the reversible binding of a phosphorylated substrate peptide to a specific phosphoamino acid binding domain triggers proximity between two tethered fluorescent proteins forming a FRET pair^15^. In Kinprola, these two fluorescent proteins are replaced by the two components of a split-HaloTag system. Phosphorylation of Kinprola then leads to reconstitution of active HaloTag, which in the presence of a fluorescent HaloTag substrate leads to its irreversible labeling. Using this modular design principle, we have generated recorders for PKA, PKC, JNK and AMPK by swapping in specific substrate peptides identified in previous studies that developed selective real-time biosensors for these kinases^15^. The phosphorylation sites and recognition motifs of the substrate peptides ensure high specificity for their cognate kinases while minimizing cross-reactivity with other kinases. Kinprola may be further extended to record the activity of other types of kinase, such as extracellular signal-regulated kinase (ERK) and tyrosine kinases, by using appropriate substrate peptides and phosphoamino acid binding domains. By tagging with distinct localization signals and fluorescent proteins, Kinprola_PKA_ variants can be used for simultaneous recording of PKA activation in distinct cellular compartments in response to the same stimulus. In principle, it should also be possible to record the activities of multiple kinases by spatially separating appropriate Kinprola variants.

The large majority of the experiments reported here were performed with Kinprola_PKA_. A key feature of Kinprola_PKA_ is the persistence of the fluorescent mark, which remains detectable for days in live neurons and is resistant to fixation procedures. This allows the sorting of cells based on their kinase activity histories from large and heterogeneous cell populations for later analysis. Transcriptome analysis of GBC subpopulations sorted according to Kinprola labeling highlights the important role of PKA in cell cycle regulation, which potentially correlates with glioblastoma proliferation and invasion. Furthermore, a Kinprola_PKA_-based CRISPR-knockout screening identified numerous putative noncanonical regulators that influence PKA activity, potentially broadening our understanding of PKA signaling and aiding in therapeutic target identification. Of note, using different cell sorting strategies, the putative hits identified by CRISPR-knockout screening in RKO cells did not significantly correlate with DEGs identified by RNA-seq in GBCs, where no genetic perturbation was introduced. This demonstrates that Kinprola recording can be tailored to address various biological questions by adjusting the recording parameters and experimental context. Compared with real-time biosensors, Kinprola can analyze fixed samples, which comes with a much higher throughput. Therefore, Kinprola should also be well suited for drug screening when combined with high-throughput methods such as flow cytometry and automated plate readers. For single-cell analysis, Kinprola can be directly integrated with in situ RNA-seq, enabling the stable recording of kinase activity alongside single-cell gene expression profiling within the same cells. This approach can also be extended to other single-cell perturbation analyses, such as high-content imaging-based CRISPR screening.

Real-time PKA activity biosensors demonstrate rapid on kinetics of PKA activity in response to neuromodulatory inputs; however, the off phase spans several minutes, probably due to the slow dissociation of cAMP from the PKA regulatory subunits (dissociation constant *K*_d_ around 0.15 min^−1^)^9,42,43^. In the presence of HaloTag substrate, Kinprola_PKA_ continuously accumulates labeling signals throughout the entire duration of one or multiple PKA activation cycles. We demonstrated that Kinprola_PKA_ is both sufficiently fast and sensitive to record PKA activation at a minute-scale resolution, even when stimulated by relatively weak and transient stimuli, such as low doses of Iso or electrophysiological stimulation in primary neurons. Three key parameters—(1) kinase activation strength, (2) HaloTag substrate concentration and (3) recording time—determine the degree of Kinprola labeling. By adjusting the substrate concentration, users of Kinprola can fine tune the responsiveness to suit their specific experimental needs. Kinprola is also a promising tool for recording PKA activity in vivo, as demonstrated by the labeling of Kinprola_PKA_ in NAc neurons with elevated PKA activity provoked by a selective D1/D5R agonist in freely moving mice. The fast in vivo clearance kinetics of fluorescent HaloTag substrates in mice (*t*_1/2_ around 15 min) restricts the recording period of Kinprola_PKA_ after a single injection of CPY-CA to minute timescales^44,45^. Extending the recording period of Kinprola *in vivo* would thus require either different delivery methods of the HaloTag substrates or the development of HaloTag substrates with more favorable pharmacokinetic properties.

PKA activity has been monitored as a central surrogate for intracellular neuromodulation throughout the nervous system, and Kinprola should thus become a complementary tool to existing fluorescent PKA activity reporters for dissecting mechanisms related to neuromodulatory activity ex vivo and in vivo. In the future, it should be possible to stably mark neurons with elevated PKA activity using Kinprola in the nervous system for transcriptome analysis and pharmacological or genetic screenings, enabling systematic studies of the molecular features of signaling pathways during neuromodulation.

## Methods

### General information

All reagents were purchased from commercial suppliers and used without further purification. Detailed information on the reagents and resource is listed in Supplementary Table 3. The fluorescent HaloTag substrates were synthesized according to literature procedures^46,47^. The JF dyes were generously provided by L. D. Lavis (Janelia Research Campus). The fluorescent substrates were dissolved in dry dimethyl sulfoxide (DMSO) to create stock solutions and subsequently diluted in the respective buffers to ensure the final DMSO concentration was within 1% (vol/vol). The chemical structures and photophysical properties of these substrates are presented in Supplementary Fig. 1. For drug treatment, an equal volume of DMSO was used as the vehicle control unless otherwise specified. The composition of common buffers used in this study is detailed in Supplementary Table 4.

### Molecular cloning

For protein production in *Escherichia* *coli*, the pET51b(+) vector (Novagen) was used. For mammalian cell expression, the pcDNA5/FRT vector (ThermoFisher Scientific) was used. Unless otherwise specified, molecular cloning was performed using Gibson assembly^48^, In-Fusion Cloning or the Q5 site-directed mutagenesis kit, following the manufacturer’s protocols. The primers were synthesized by Sigma-Aldrich or Eurofins. DNA amplification was carried out by PCR using KOD-hot-start DNA polymerase master mix or Q5 high-fidelity DNA polymerase. The PCR products were purified using the QIAquick PCR purification kit or the NucleoSpin Gel and PCR Clean-up kit. The cloning products generated by Gibson assembly were electroporated into *E. coli* strain 10G. For cloning virus-related plasmids, NEB stable competent *E. coli* and One Shot Stbl3 chemically competent *E. coli* were used. The plasmids were purified with the QIAprep Spin Miniprep Kit. For cell transfection purpose, endotoxin was removed using the GeneJET Endo-Free Plasmid Maxiprep Kit. All the sequences were verified by Sanger sequencing, and the integrity of inverted terminal repeats recombination sites of virus-related plasmids was also verified. All the plasmids used in this study can be found in Supplementary Table 5.

### Single sgRNA plasmid cloning

For cloning individual sgRNA sequences into the HDCRISPRv1 vector^36^, the vector was sequentially digested with BfuAI and BsrGI-HF, followed by dephosphorylation using Quick CIP. The digested vector was then purified by gel electrophoresis. The annealed oligos were ligated into digested vector using the Quick Ligation Kit according to the manufacturer’s protocol. All the sgRNA sequences can be found in Supplementary Table 6.

### Protein expression and purification

The proteins were expressed in *E. coli* strain BL21(DE3). The lysogeny broth cultures were grown at 37 °C until the optical density at 600 nm (OD 600 nm) reached 0.8. Protein expression was induced by adding 0.5 mM isopropyl-β-d-1-thiogalactopyranoside (IPTG), and the cultures were then grown at 16 °C overnight. The cell cultures were collected by centrifugation (4,500*g*, 10 min, 4 °C) and lysed by sonication on ice in IMAC lysis buffer (Supplementary Table 4). The cell lysate was cleared by centrifugation (70,000 g, 20 min, 4 °C). The proteins were purified using HisPur Ni-NTA Superflow Agarose or on the ÄktaPure FPLC system (Cytiva) with IMAC wash and elution buffer (Supplementary Table 4) and concentrated using Ultra-15 centrifugal filter units with a molecular weight cutoff smaller than the protein size, followed by buffer exchange into the activity buffer (Supplementary Table 4, final imidazole concentration <0.1 mM). The purification His_10_ tag was removed by overnight cleavage with tobacco etch virus (TEV) protease at 30 °C as previously described^49^. The cleaved proteins were purified using a HisTrap FF crude column (Cytiva) on the ÄktaPure FPLC system (Cytiva) by collecting the flow-through. The proteins were further purified by size-exclusion chromatography (HiLoad 26/600 Superdex75, Cytiva) using the activity buffer. The purified proteins were either flash frozen in liquid nitrogen and stored at −80 °C or mixed 1:1 (vol/vol) with 90% (wt/vol) glycerol in activity buffer and stored at −20 °C. The final protein concentration ranged from 100 to 500 μM. The correct size and purity of proteins were assessed by SDS–polyacrylamide gel electrophoresis and liquid chromatography–mass spectrometry. The protein amino acid sequences are listed in Supplementary Note 1.

### In vitro phosphorylation of Kinprola_PKA_ protein

In a 500-µl kinase assay buffer system (Supplementary Table 4), 10 µM of purified Kinprola_PKA_ protein was mixed with 10 µg of PKAcat and 200 µM of ATP, then incubated at 30 °C for 1 h with shaking at 300 rpm. The phosphorylated proteins were purified by size-exclusion chromatography and assessed by liquid chromatography–mass spectrometry.

### Protein thermostability measurement

The protein thermostability was measured at 0.5 mg ml^−1^ in activity buffer using a nanoscale differential scanning fluorimeter (NanoDSF) Prometheus NT 48 device (NanoTemper Technologies). The temperature range for measurement was from 20 °C to 95 °C with a heating rate of 1 °C min^−1^. The changes in the ratio of the fluorescence intensities at 350 and 330 nm were monitored. The indicated melting temperature (mean of two technical replicates) corresponds to the point of inflection (maximum of the first derivative). The melting temperatures of the proteins are listed in Supplementary Table 1.

### Kinprola labeling kinetics

The labeling kinetics of Kinprola was measured by recording fluorescence polarization over time at 30 °C using a Tecan microplate reader. The measurements were conducted in black, nonbinding, flat bottom, 96-well polystyrene plates (OptiPlate, PerkinElmer). The experiments were performed in kinase assay buffer (Supplementary Table 4) with or without PKAcat and ATP, in technical triplicates. In a 200 μl assay system, the final concentrations were as follows: 200 nM Kinprola protein, 25 ng μl^−1^ PKAcat, 500 μM ATP and 50 nM fluorescent HaloTag substrate (CPY-CA, TMR-CA, JF_549_-CA, JF_552_-CA or JF_669_-CA). Kinprola protein and fluorescent substrate were prepared separately in 50 μl aliquots, and PKAcat + ATP was prepared in 100 μl. Kinprola protein was first incubated with kinase assay buffer containing PKAcat + ATP for 30 min at 30 °C. The reactions were then started by adding 50 μl of the fluorescent HaloTag substrate. The control experiments were conducted with the same procedure but in kinase assay buffer without PKAcat and ATP. The data analysis was performed as previously described^17^. Kinprola labeling kinetics parameters are listed in Supplementary Table 2.

### Cell culture

HeLa Kyoto Flp-In (provided by Dr. Amparo Andres-Pons, EMBL), human embryonic kidney 293 (HEK293) Flp-In T-REx cells and HEK293T cells were cultured in Dubeco’s modified Eagle medium supplemented with 10% (vol/vol) fetal bovine serum (FBS) in a humidified 5% CO_2_ incubator at 37 °C. The RKO cells were cultured in RPMI + GlutaMAX medium supplemented with 10% (vol/vol) FBS, 100 units ml^−1^ penicillin and 100 µg ml^−1^ streptomycin. The primary rat hippocampal neurons were cultured in NeuroBasal medium supplemented with 1× GlutaMAX, 1× B-27, 100 units ml^−1^ penicillin and 100 µg ml^−1^ streptomycin. The patient-derived GBC line (PDGCL) S24 (refs. ^31,50^) was cultured as nonadherent neurospheres in PDGCL medium, consisting of Dubeco’s modified Eagle medium/F-12, 1× B-27 supplement, 5 μg ml^−1^ insulin, 5 μg ml^−1^ heparin, 20 ng ml^−1^ epidermal growth factor and 20 ng ml^−1^ basic fibroblast growth factor. The cell lines were regularly tested for mycoplasma contamination and were mycoplasma-free.

### Transient transfection of cells

Transient transfection was performed using Lipofectamine 3000 transfection reagent unless otherwise specified. For transfecting a single well of a 96-well plate, 100 ng of DNA was first diluted into 10 μl of Opti-MEM I and mixed with 0.2 μl of P3000. Separately, 0.2 μl of Lipofectamine 3000 was diluted with 10 μl of Opti-MEM I. The two solutions were then mixed and incubated for 15 min at room temperature. The prepared DNA–Lipofectamine complex was added to cells at 50–70% confluency. The medium was changed after 12 h of incubation in a humidified 5% CO_2_ incubator at 37 °C. The cells were then cultured under the same conditions for another 12–36 h before further treatment.

### Stable cell line establishment

HeLa and HEK293 stable cell lines were generated using the Flp-In system. Briefly, HEK293 Flp-In T-Rex or HeLa Kyoto Flp-In cells were cultured to 80% confluency in T-25 cell culture flasks. The cells were then cotransfected with 440 ng of a pCDNA5/FRT plasmid encoding the gene of interest (GOI) and 3,560 ng of the pOG44 Flp-recombinase expression plasmid (Invitrogen) using the Lipofectamine 3000 transfection reagent as described above. The cell culture medium was changed 12 h post transfection, and the medium was replaced with cell culture medium supplemented with 100 μg ml^−1^ hygromycin B 24 h post transfection to select cells that stably integrated the GOI into the genome. After 48–72 h of selection, the cells were recovered in fresh cell culture medium until reaching confluency. The cells with high expression levels of GOI were sorted in bulk population on the basis of EGFP fluorescence intensity (blue laser, 488 nm with 530/30 bandpass filter) using FACS with a FACSMelody cell sorter (BD Biosciences). A list of established stable cell lines can be found in Supplementary Table 5.

### rAAV preparation

The recombinant AAVs (rAAVs) used for cell experiments were produced as previously described^51^. Briefly, HEK293 cells were transfected with plasmids pRV1 (containing AAV2 Rep and Cap sequences), pH21 (containing AAV1 Rep and Cap sequences), pFD6 (adenovirus helper plasmid) and the AAV plasmid containing the recombinant expression cassette driven by the hSyn1 or CAG promoter and flanked by AAV2 packaging signals (inverted terminal repeats recombination sites). Transfection was performed using polyethylenimine (PEI) 25,000. A total of 5 days post transfection, the culture medium and cells were collected by centrifugation at 1,000*g* for 5 min at 4 °C. The cells were lysed using TNT extraction buffer (Supplementary Table 4). The cell debris was removed by centrifugation at 3,000*g* for 5 min at 4 °C. The supernatant was treated with 50 U ml^−1^ benzonase nuclease for 30–60 min at 37 °C, with mixing by inverting every 20 min. The rAAVs were purified from the medium and supernatant via Äkta-Quick FPLC system (Cytiva) with AVB Sepharose HiTrap columns (Cytiva). The columns were first equilibrated with PBS (pH 7.4), and the virus particles were then eluted with 50 mM glycine–HCl (pH 2.7). The purified virus particles were concentrated and buffer exchanged to PBS (pH 7.3) using Amicon Ultra centrifugal filters (Millipore) with a molecular weight cutoff of 100 kDa. The rAAVs were aliquoted, flash frozen and stored at −80 °C until further use. The rAAV titer was quantified by quantitative PCR as previously described^52^. For mice brain injection and expression, AAV9-hSyn-Kinprola_PKA_ and AAV9-hSyn-Kinprola_PKA_T/A_ were packaged at BrainVTA.

### Lentivirus production

Lentivirus were produced as previously described^36^. Briefly, the lentiviral packaging vector psPAX2 and the lentiviral envelope vector pMD2.G were cotransfected with the respective lentiviral expression vector (in a ratio of 5.25:3.15:10) using TransIT-LT1 transfection reagent and Opti-MEM, according to the manufacturer’s protocol, into low-passage (<15) HEK293T cells at 70–80% confluency. Approximately 16 h post transfection, the medium was replaced with fresh culture medium and supernatant was collected 48 h post transfection by filtration through a 0.45-μm low protein binding PES membrane. The collected lentiviral supernatant was aliquoted and stored at −80 °C.

### Cell fixation

After treatment and labeling, the cells were washed with prewarmed PBS and fixed with 4% (wt/vol) methanol-free formaldehyde (PFA) in PBS at 37 °C for 15 min. Subsequently, the cells were washed three times with PBS for further imaging.

### Recording PKA activities in cells during pharmacological treatment

In general, the cells expressing Kinprola_PKA_ variants were seeded into chambered coverslips or 96-well culture dishes and grown to approximately 80% confluency. The cells were treated with various compounds (50 μM Fsk/100 μM IBMX, 1 μM PGE1, 10 μM Iso, 1 μM epinephrine, 1 mM Bt_2_cAMP, 20 μM H89, 100 nM thapsigargin, 100 ng ml^−1^ phorbol 12-myristate 13-acetate (PMA), 10 μM anisomycin, 1 μM ionomycin and 40 mM 2-deoxy-d-glucose (2-DG)) in the presence of 25 nM CPY-CA for 30 min in a humidified incubator at 37 °C with 5% CO_2_ atmosphere. After treatment and labeling, the cells were then washed with PBS and incubated with medium supplemented with 5 μM recombinant HaloTag protein for 10 min. Subsequently, the cells were washed again with PBS and either fixed for fluorescence imaging or detached with transparent TrypLE Express Enzyme for flow cytometry analysis.

### Real-time recording of labeling signal integration in HEK293 cells

The HEK293 cells stably expressing Kinprola_PKA_ were seeded into poly-d-lysine-coated eight-well imaging chambered coverslips and cultured in 150 μl of transparent medium per well. During timelapse imaging, five baseline frames were first captured. Then, the medium was replaced with 150 μl of medium containing 25 nM CPY-CA, followed by a 10-min imaging acquisition period. Afterward, 150 μl of medium containing 25 nM CPY-CA and 100 μM Fsk was added, and another 5-min imaging acquisition was performed. Finally, 150 μl of medium containing 25 nM CPY-CA, 50 μM Fsk and 60 μM H89 was added for approximately 15 min of imaging acquisition. The final concentrations of CPY-CA, Fsk and H89 in the imaging medium were maintained constant at 25 nM, 50 μM and 20 μM, respectively. Signal background from free CPY-CA was subtracted using cocultured cells without Kinprola_PKA_ expression.

### Successive recordings during pharmacological treatment in HEK293 cells

The HEK293 cells stably expressing Kinprola_PKA_ were seeded into poly-d-lysine-coated 96-well imaging plates. For successive recordings with spectrally distinguishable fluorescent HaloTag substrates, the cells were sequentially treated as follows: (1) cotreated with 10 μM PGE1 and fluorophore substrate 1 for 30 min, (2) cotreated with 10 μM H89 and fluorophore substrate 2 for 30 min and (3) cotreated with 50 μM Fsk/100 μM IBMX and fluorophore substrate 3 for 20 min. Between different recordings, the cells were washed with medium supplemented with 5 μM recombinant HaloTag protein for 10 min, followed by two washes with medium and then allowed to rest for 2 h in fresh medium in a humidified incubator at 37 °C with 5% CO_2_ atmosphere. For these recordings, the following substrates were used: 25 nM CPY-CA, 100 nM JF_552_-CA and 100 nM JF_669_-CA. After the final recording session, the cells were fixed for further imaging as described above.

### Recording activities of different kinases in cells during pharmacological treatment

HeLa or HEK293 cells were seeded into 96-well culture dishes and grown to approximately 50% confluency. For HEK293 cells, the 96-well culture dishes were precoated with poly-d-lysine. Kinprola variant plasmids were then transfected into cells using Lipofectamine 3000. A total of 24 h post transfection, the cells were treated with various compounds (for Kinprola_PKC_ and Kinprola_PKC_T/A_, 100 ng ml^−1^ PMA, 1 μM Gö 6983; for Kinprola_JNK_ and Kinprola_JNK_T/A_, 10 μM anisomycin, 10 μM JNK inhibitor III; for Kinprola_AMPK_ and Kinprola_AMPK_T/A_, 40 mM 2-DG, 30 μM SBI-0206965 (SBI) in the presence of 25 nM CPY-CA for 30 min in a humidified incubator at 37 °C with 5% CO_2_ atmosphere. After treatment, the cells were then washed with PBS and incubated with medium supplemented with 5 μM recombinant HaloTag protein for 10 min. Subsequently, the cells were washed again with PBS and detached with transparent TrypLE Express Enzyme for flow cytometry analysis.

### Simultaneously recording PKA activities in different cellular compartments during pharmacological treatment

The HeLa cells were transduced using AAVs under a CAG promoter with nuclear export signal (NES)-Kinprola_PKA_-mTagBFP2 and Kinprola_PKA_-mEGFP-NLS 3×. A total of 36 h post transduction, the cells were stimulated with 50 μM Fsk/100 μM IBMX in the presence of 50 nM CPY-CA for 30 min in a humidified incubator at 37 °C with 5% CO_2_ atmosphere. After treatment, the cells were then washed with PBS and incubated with medium supplemented with 5 μM recombinant HaloTag protein for 10 min. Subsequently, the cells were washed again with PBS and fixed for fluorescence imaging.

### Generation of GBCs stably expressing Kinprola

The Kinprola expression cassette was cloned into a pLKO.1-puro vector for lentivirus production. Lentivirus transduction of glioblastoma S24 cells was performed as previously described^28^. Successfully transduced S24 cells were selected using 1 μg ml^−1^ puromycin. The EGFP-positive cells were subsequently sorted using a FACSMelody cell sorter (BD Biosciences, excitation 488 nm, filter 530/30 nm) and propagated for further use.

### RNA-seq data generation

The RNA-seq sample preparation was conducted as previously described with modifications^17^. Glioblastoma S24 cells stably expressing Kinprola_PKA_ or Kinprola_on_ were plated at a density of 4 × 10^6^ cells onto Matrigel-coated T-25 cell culture flasks in growth factor-devoid PDGCL medium supplemented with 50 mM glucose (high-glucose medium, HGM). These serum-free stem-like conditions preserve both the gene expression and biological properties of the original tumor, such as diffuse growth and network formation^53^. A total of 48 h after seeding, the Kinprola_PKA_-expressing S24 cells were labeled with 100 nM CPY-CA for 30 min at 37 °C in a humidified incubator with 5% CO_2_ atmosphere, while Kinprola_on_-expressing S24 cells were labeled for a decreased time of 2.5 min as a control. Afterward, the cells were rinsed with HGM and incubated with HGM containing 4 μM recombinant HaloTag protein for 10 min. The cells were then rinsed with PBS, detached using accutase, resuspended in cold PBS and subjected to FACS sorting on a FACSAria Fusion Special Order System (BD Biosystems). Kinprola_PKA_- or Kinprola_on_-expressing S24 cells were sorted into three groups on the basis of high (around 5%), medium and low (around 5%) normalized fluorescence intensity (CPY-CA/EGFP). Three replicate samples per group were collected, and the RNA was isolated with the Arcturus PicoPure Frozen RNA Isolation Kit, according to the manufacturer’s instructions. On-column DNase digestion was performed using the RNase-Free DNase Set, and RNA integrity was verified using the high-sensitivity RNA ScreenTape System (Agilent) and the 4150 Tapestation System (Agilent). Library preparation and RNA-seq of the three replicates per condition were performed on a NovaSeq6000 device (Illumina) by the Genomics and Proteomics Core Facility at the German Cancer Research Center (DKFZ, Heidelberg).

### RNA-seq data analysis

The RNA-seq reads were aligned with STAR^54^ (v.2.5.3a) against the GRCh38 human reference genome. A gene-count matrix was generated using featureCounts^55^ in Subread (v.1.5.3) against GENCODE^56^ (v.32). The pairwise DEGs between groups were identified with generalized linear models using DeSeq2 (ref. ^57^) (v1.38.3) (that is, DEGs in ‘high’ versus ‘medium’, ‘high’ versus ‘low’ and ‘medium’ versus ‘low’). The DEGs were retained with a FDR <0.05, a raw count >9 in all samples and replicates, a log_2_ fold change (log_2_FC) >0.5 and log_2_FC <−0.5 and those recurrently identified in all three comparisons. The analysis yielded 737 DEGs in Kinprola_PKA_ data and 326 DEGs in Kinprola_on_ data. The expression levels (log_2_ fragments per kilobase per million mapped fragments) of DEGs were *Z*-score scaled across samples and visualized with GraphPad Prism (version 10.2.1). The multidimensional scaling plots of RNA-seq datasets with the principal component analysis (PCA) were plotted using the plotPCA function after normalization with the vst() function in DESeq2 (ref. ^57^). ShinyGO^58^ (RRID: SCR_019213, v.0.741 and v.0.80) was used for a GO enrichment analysis. The FDR and fold enrichment were calculated by comparing DEGs lists with a background of all protein-coding genes in the human genome.

### Generation of RKO cells stably expressing Kinprola and Cas9

The Kinprola expression cassette was cloned into a lenti-EF1α-Cas9-T2A-Blasticidin expression plasmid (Supplementary Table 5 and Supplementary Note 1). Lentivirus production was performed as described above. Successfully transduced RKO cells were selected using 15 μg ml^−1^ Blasticidin for 7 days. The EGFP-positive cells were sorted using a FACSMelody cell sorter (BD Biosciences, excitation 488 nm, filter 530/30 nm) and propagated for further use.

### Recording PKA activities during pharmacological treatment in RKO cells

The RKO cells stably expressing Kinprola_PKA_, Kinprola_PKA_T/A_ and Kinprola_on_ were seeded at 8 × 10^3^ cells per well into 96-well plates. A total of 2 days post seeding, the cells were incubated with vehicle or with compounds (50 μM Fsk/100 μM IBMX or 20 μM H89) in the presence of 125 nM CPY-CA for 30 min at 37 °C in a humidified incubator with 5% CO_2_ atmosphere. The cells were then rinsed with fresh medium, incubated with medium containing 5 μM recombinant HaloTag protein for 10 min, rinsed with PBS, detached using transparent TrypLE Express Enzyme, resuspended into PBS containing 2% (vol/vol) FBS and finally analyzed via flow cytometry.

### Lentiviral CRISPR sgRNA library preparation

The HD CRISPR library sublibrary A was constructed on an HD CRISPR vector as previously described^36^. Briefly, the lentivirus was produced using HEK293T cells as the host. Together with psPAX2 and pMD2.G packaging plasmids, the sgRNA plasmid library was transfected into HEK293T cells using TransIT-LT1 transfection reagent. A total of 48 h post transfection, the virus-containing supernatant was filtered through a 0.45-μm low protein binding PES membrane, aliquoted and stored at −80 °C until further use. For determining the MOI, the RKO cells were transduced with varying amounts of lentiviral supernatant in the presence of 10 μg ml^−1^ polybrene according to the manufacturer’s protocol. A total of 24 h post transduction, the cells were selected with 2 μg ml^−1^ puromycin for another 48 h, and the number of surviving cells was compared with a nontransduced control sample. The titer of HD CRISPR sublibrary A in RKO cells, as produced in this study, was determined to be 2.19 × 10^7^ transduction units per milliliter.

### Pooled CRISPR-knockout screen

A total of 2.5 × 10^8^ RKO cells stably expressing Kinprola_PKA_ and Cas9 were transduced with the lentiviral HD CRISPR sublibrary A and 10 μg ml^−1^ polybrene in five-layer cell culture multiflasks to achieve an initial library coverage of around 500-fold upon infection at an MOI of 0.2–0.3 (ensuring the large majority of cells receive only one sgRNA for gene editing). A total of 24 h post transduction, the transduced cells were selected with 2 μg ml^−1^ puromycin. One flask was cultured without puromycin selection for MOI determination. Then, 48 h post selection, the cells were passaged and the MOI was determined by calculating the ratio of live cells with and without puromycin selection. The cells were reseeded at a 500-fold library coverage and cultured for another 2 days. To prepare labeled cells for sorting, the cells were incubated with 125 nM CPY-CA for 30 min at 37 °C in a humidified incubator with 5% CO_2_ atmosphere. The cells were further rinsed with PBS and washed with medium containing 2 μM recombinant HaloTag protein for 15 min. Then the cells were detached with accutase, pelleted by centrifugation at 300*g* for 5 min, resuspended in ice-cold PBS supplemented with 2% (vol/vol) FBS and 5 mM EDTA, filtered with cell strainers and stored on ice until sorting. The labeled cells were sorted into three groups with the high (around 25%), medium (around 50%) and low (around 25%) normalized fluorescence intensity (CPY-CA/EGFP). Once sorted, the cells were pelleted by centrifugation at 300*g* for 5 min, washed with PBS and stored dry at −20 °C until genomic DNA extraction.

### Genomic DNA extraction, library preparation and sequencing

These steps were performed as previous described with modifications^36^. Briefly, genomic DNA was isolated from frozen cell pellets using the QIAamp DNA Blood and Cell Culture DNA Maxi Kit according to the manufacturer’s instructions and purified by ethanol precipitation. The DNA concentration in all subsequent steps was measured using Nanodrop or Qubit dsDNA HS and BR Assay Kits. For PCR amplification of the sgRNA cassette, 100 μg of genomic DNA was used with unique indexing primers for each sample. Illumina adapters, and indices were added in the same one-step PCR reaction using the KAPA HiFi HotStart ReadyMix. The PCR product was purified using the QIAQuick PCR Purification Kit and further gel purified to remove genomic DNA contamination using the QiaQuick Gel Extraction kit. The quality and purity of the amplified PCR product were determined using the Agilent 2100 Bioanalyzer system. DNA concentrations from all conditions were adjusted and pooled at equimolar ratios. The sequencing reaction was performed on an Illumina NextSeq 500/550 system with a High-Output Kit v2.5 (75 cycles) to read the 20-nt sgRNA sequence and quantify the number of copies, by the Deep Sequencing Core Facility (Bioquant, Heidelberg University).

### Pooled CRISPR-knockout screen data processing and analysis

The absolute sgRNA read counts were collected and demultiplexed using the MAGeCK version 0.5.9.4 package^59^. To process the raw data, the read counts were first normalized and log-transformed. The fold changes between conditions were then determined by subtracting the log-normalized read count of the control samples from that of the corresponding treated sample. The replicates were collapsed by arithmetic mean for each gene and each sgRNA. The statistical significance of differential gene expression was calculated using the LIMMA package^60^. The *P* values were corrected for multiple testing by Benjamini−Hochberg correction. An FDR cutoff of 0.05 was applied to hit selection. The hit list was further filtered by eliminating RKO core-essential genes according to dependency score available in the Cancer Dependency Map Project (DepMap, https://depmap.org/).

### Hits validation by individual retests of select sgRNAs

Two NT sgRNAs and two sgRNAs targeting each hit of interest were cloned into the parental HDCRISPRv1 (ref. ^36^) vector for sgRNA expression under a U6 promoter (Supplementary Table 6). The RKO cells stably expressing Kinprola_PKA_ and Cas9 were seeded into six-well plates at a density of 5 × 10^5^ cells per well. After 24 h, the cells were transfected with plasmids containing the sgRNAs with jetOPTIMUS transfection reagent. For a single well transfection, 3 μg of plasmid and 3 μl of jetOPTIMUS transfection reagent in 200 μl of jetOPTIMUS buffer were used. The cells were changed into new culture medium after 6 h. A total of 24 h after transfection, the cells were selected with new culture medium supplemented with 1.25 μg ml^−1^ puromycin. After 48 h of selection, the cells were labeled with 125 nM CPY-CA for 30 min and prepared as described above for flow cytometry. SYTOX blue dead cell stain was used for gating out dead cells according to manufacturer’s protocol. To evaluate sgRNA cutting efficacy, genomic DNA was extracted using the QIAamp DNA Blood Maxi Kit. The fragments flanking the sgRNA target sites were amplified by PCR, purified by gel extraction using the QIAquick Gel Extraction Kit and sequenced via Sanger sequencing. The editing efficacy was then analyzed using Tracking of Indels by Decomposition (TIDE)^61^ by comparing chromatogram sequence files from edited samples and the NT control. The primers used for amplifying the genomic regions are listed in Supplementary Table 7.

### ELISA-based colorimetric PKA activity measurement

The PKA activity in cell lysate was measured using an ELISA-based PKA colorimetric activity kit according to the manufacturer’s instructions. Briefly, sgRNA-transfected cells, prepared as described above, were collected using a cell scraper and lysed in activated cell lysis buffer (Supplementary Table 4) for 30 min on ice with occasional vortexing, followed by centrifugation at 9,391*g* for 10 min at 4 °C. The supernatants were then frozen as single-use aliquots at −80 °C. The protein concentrations were quantified using a Bicinchoninic acid assay before the following colorimetric assay. The colorimetric assay was performed according to the manufacturer’s protocol, and the optical density was measured using a Tecan microplate reader.

### Western blot analysis

The cells were washed twice with ice-cold PBS and lysed using RIPA lysis buffer supplemented with complete protease inhibitor cocktail and complete PhosSTOP. The cells were scraped from the six-well plates, and the lysates were incubated at 4 °C with rotational agitation for 30 min to ensure complete lysis. The lysates were then clarified by centrifugation at 21,130*g* for 20 min at 4 °C. The protein concentrations were determined using the BCA Protein Assay Kit according to the manufacturer’s instructions. The protein samples were denatured in 1× Laemmli buffer by heating at 95 °C for 5 min and then loaded onto Bolt 4-12% Bis–Tris Plus Protein Gels. The proteins were separated by SDS–polyacrylamide gel electrophoresis using 3-(N-morpholino)propanesulfonic acid (MOPS) running buffer alongside a Pageruler Prestained Protein Ladder. Following electrophoresis, the proteins were transferred to polyvinylidene difluoride membranes via wet transfer in a buffer containing 10% (vol/vol) methanol. The transfer efficiency was confirmed by staining the membranes with Ponceau S solution. The membranes were destained with Tris-buffered saline with Tween-20 (TBST) and blocked with 5% (wt/vol) skim milk in TBST for 30 min at room temperature. The membranes were then incubated with primary antibodies overnight at 4 °C or for 2 h at room temperature on a roller mixer. After incubation, the membranes were washed three times for 5 min each with TBST and incubated with horseradish peroxidase (HRP)-conjugated secondary antibodies for 1 h at room temperature. The membranes were washed again three times for 5 min each with TBST. The protein signals were detected using enhanced chemiluminescence substrates and visualized using a ChemiDoc Imaging System with Image Lab software (Bio-Rad, version 6.1.0). All antibodies were diluted in 5% skim milk in TBST. The following antibodies and dilution were used: FZR1 polyclonal antibody (Proteintech, 16368-1-AP, 1:1,000), TRIM33 polyclonal antibody (Proteintech, 55374-1-AP, 1:1,000), PES1 polyclonal antibody (Proteintech, 13553-1-AP, 1:1,000), VPRBP polyclonal antibody (Proteintech, 11612-1-AP, 1:1,000), NOP2 polyclonal antibody (Proteintech, 10448-1-AP, 1:1,000), anti-vinculin antibody (Sigma-Aldrich, V9264, 1:10,000), HRP-AffiniPure polyclonal goat anti-mouse IgG (H + L) (Jackson ImmunoResearch, JIM-115-035-003, 1:10,000), HRP-AffiniPure polyclonal goat anti-rabbit IgG (H + L) (Jackson ImmunoResearch, JIM-111-035-003, 1:10,000).

### Primary rat hippocampal neurons preparation

All procedures were conducted in strict accordance with the Animal Welfare Act of the Federal Republic of Germany (Tierschutzgesetz der Bundesrepublik Deutschland, TierSchG) and the Animal Welfare Laboratory Animal Regulations (Tierschutzversuchsverordnung). According to these regulations, no ethical approval from an ethics committee is required for euthanizing rodents when the organs or tissues are used for scientific purposes. The euthanasia procedure for rats in this study was supervised by animal welfare officers of the Max Planck Institute for Medical Research and was carried out and documented in compliance with the TierSchG (permit number assigned by the Max Planck Institute for Medical Research: MPI/T-35/18). The primary rat hippocampal neurons were prepared from isolated hippocampi obtained from postnatal P0–P1 Wistar rats of both sexes, following the established protocols as previously described^62^. The neurons were seeded onto poly‑l‑ornithine (100 μg ml^−1^ in water) and laminin (1 μg ml^−1^ in HBSS) 24-well or 96-well glass bottom imaging plates and maintained in a humidified cell culture incubator with 5% CO_2_ at 37 °C.

### rAAV transduction

At day 6, the neurons were refreshed with one-third of the medium. On day 7, the neurons were transduced with purified rAAVs (serotype 2/1) at concentrations ranging from 10^9^ to 10^10^ genome copies per milliliter. The cultures were allowed to express transgenes for 7 days. One-third of the medium was changed every 3 days during this period.

### Recording PKA activities in cultured neurons during pharmacological treatment

The neurons seeded in 96-well glass bottom imaging plates were used at 14–16 days in vitro. The PKA modulators (50 μM Fsk/2 μM Rol, 1 μM Iso, 1 μM propranolol, 1 μM norepinephrine or 20 μM H89) were applied along with 25 nM CPY-CA to neuronal cultures. The treatments were conducted in a humidified cell culture incubator with 5% CO_2_ at 37 °C for 45 min. Afterward, the neurons were rinsed with warm NeuroBasal medium, followed by incubation with warm NeuroBasal medium supplemented with 5 μM recombinant HaloTag protein for 15 min. The neurons were then washed with warm HBSS and fixed with 4% (wt/vol) PFA for 15 min at 37 °C. After fixation, the neurons were washed with HBSS and stored at 4 °C for further imaging.

### Recording PKA activities in cultured neurons during electric field stimulation

The cultured neurons in 24-well glass bottom imaging plates were prepared for electric field stimulation using a custom-build 24-well cap stimulator equipped with platinum electrodes connected to a stimulation control unit, as previously described^63^. Before stimulation, the neurons were treated with a synaptic blocker solution (25 μM APV/10 μM NBQX in NeuroBasal medium) for 25 min at 37 °C in a humidified cell culture incubator with 5% CO_2_. The neuron cultures were then transferred to a widefield microscope stage housed in an environmental chamber set to 37 °C with 5% CO_2_. The cap stimulator was positioned on top of the neuron cultures. The neurons were preincubated with 125 nM CPY-CA for 5 min before stimulation. The stimulation patterns were set to 80 Hz frequency, 100 mA intensity and 1 ms pulse width, generating defined trains of action potentials in the presence of CPY-CA. Following electric field stimulation, the neurons were washed and fixed for further imaging, following the procedures described above.

### Stability measurement of fluorescent Kinprola_PKA_ labeling signal in cultured neurons

The neurons were incubated with 125 nM CPY-CA for 1 h at 37 °C in a humidified cell culture incubator with 5% CO_2_ and then washed as described above. The experiments were conducted in three 24-well imaging plates as replicates. The fluorescent intensities of basally active neurons were recorded and measured at 24-h intervals over a period of 3 days. Between measurements, the neurons were maintained at 37 °C in a humidified cell culture incubator with 5% CO_2_.

### Animals

All procedures for animal surgery and experimentation were performed using protocols approved by the Institutional Animal Care and Use Committee at Peking University. The mice were group- or pair-housed in a temperature-controlled (18–23 °C) and humidity-controlled (40–60%) room with a 12-h light–dark cycle. Food and water were available ad libitum.

### Expression of Kinprola in mice brain

Male C57BL/6N mice (6–8 weeks of age) were anesthetized with an intraperitoneal injection of 2,2,2-tribromoethanol (Avertin; 500 mg kg^−1^ of body weight). The AAV9-hSyn-Kinprola_PKA_ (500 nl, 2.5 × 10^12^ viral genomes per millliliter, BrainVTA) and AAV9-hSyn-Kinprola_PKA_T/A_ (300 nl, 6 × 10^12^ viral genomes per millliliter, BrainVTA) viruses were injected into the bilateral NAc separately (anteroposterior (AP): +1.4 mm relative to Bregma; mediolateral (ML): ±1.2 mm relative to Bregma; dorsoventral (DV): −4.0 mm from the dura) at a rate of 50 nl min^−1^. The experiments were performed 2−3 weeks after virus injection.

### Acute mouse brain slices preparation

The mice were anesthetized with 2,2,2-tribromoethanol (Avertin, 500 mg kg^−1^ body weight) and perfused with ice-cold oxygenated slicing buffer (Supplementary Table 4). The brains were then dissected, and the coronal slices of 300 μm thickness were obtained using a VT1200 vibratome (Leica) in ice-cold oxygenated slicing buffer. These slices were then transferred in oxygenated artificial cerebrospinal fluid (ACSF) (Supplementary Table 4) and allowed to recover for at least 30 min at 34 °C.

### Recording PKA activities in acute mouse brain slices during pharmacological treatment

The acute brain slices were moved to a custom-made perfusion chamber and placed on the stage of an upright LSM 710 confocal microscope (ZEISS). During timelapse imaging experiments, the slices were perfused with oxygenated ACSF containing 250 nM CPY-CA with or without 50 μM Fsk/2 μM Rol. After imaging, the slices were transferred to oxygenated ACSF containing 2 μM recombinant HaloTag protein for 10 min. Subsequently, the brain slices were fixed with 4% (wt/vol) PFA at 4 °C overnight, followed by washing with 0.2% (vol/vol) Tween 20 in PBS for 2 h. For further imaging on a SP8X confocal microscope (Leica), the fixed brain slices were mounted using Fluoromount-G (SouthernBiotech) in a custom-made imaging chamber. For chamber preparation, a coverslip (no. 1.5, ~0.17 mm thick, Paul Marienfeld) were cut to the desired size to serve as a spacer. Two spacers were stacked (~0.34 mm thick), and two pairs of these stacks were placed at either side of a coverslip to form an imaging chamber, which was then sealed with epoxy before imaging.

### Recording neuromodulation-induced PKA activation in mice brain during SKF-81297 treatment

CPY-CA solution for in vivo administration were prepared as previously described^64^. Briefly, 100 nmol of CPY-CA was first dissolved in 20 μl DMSO. Then, 20 μl of a Pluronic F-127 solution (20% (wt/wt) in DMSO) was added and mixed by pipetting. This stock solution was diluted into 100 μl sterile saline. The dye solution was prepared freshly before injection to avoid freeze–thaw cycles. Before experiments, the mice were placed in individual clean cages without food and water for 1 h of habituation over two consecutive days. A total of 2 weeks post viral expression, the mice were first injected with 100 nmol of CPY-CA solution via tail vein (intravenous). After 10 min, the mice received intraperitoneal injections of SKF-81297 (10 mg kg^−1^, diluted in saline, 300 μl) or vehicle (equal volume of DMSO diluted in saline, 300 μl). The mice were placed in separated clean cages without food and water following the injection and were sacrificed 50 min after the IP injections. The mice were perfused with cold PBS supplemented with 50 μg ml^−1^ heparin, followed with cold 4% (wt/vol) PFA in PBS. The brains were dissected and fixed overnight at 4 °C in 4% (wt/vol) PFA in PBS. The brains were then dehydrated with 30% (wt/vol) sucrose solution, embedded into optimal cutting temperature (OCT) compound and sectioned in the coronal plane at 40 μm thickness using a CM1900 cryostat (Leica). The brain slices were washed three times with 0.2% (vol/vol) Tween 20 in PBS and mounted using Fluoromount-G for further imaging.

### Flow cytometry

Unless otherwise specified, the labeled cells were detached from culture plates using transparent TrypLE Express Enzyme and suspended in PBS containing 2% (vol/vol) FBS and transferred into U-shaped-bottom 96-well microplates. The cell samples were subjected to the autosampler of a BD LSRFortessa X-20 flow cytometry analyzer. The fluorescence recording parameters were set as follows: EGFP (excitation 488 nm, emission 530/30 nm), fluorophore excitation in 525–570 nm range (excitation 561 nm, emission 586/15 nm) and fluorophore excitation in 620–680 nm range (excitation 640 nm, emission 670/30 nm). Photomultiplier tube detectors were adjusted to prevent signal saturation. The same recording parameters were used consistently throughout a set of experiment.

### Flow cytometry analysis

The raw data obtained from flow cytometry was imported into FlowJo suite (version 10.10.0) and processed as follows. First, live (SSC-A/FSC-A) and single-cell (SSC-H/SSC-A) gates were gated and cells with EGFP fluorescence intensities below certain attribute unit (for example, 10^3^ attribute unit) were excluded from further analysis to minimize background noise. The fluorescence intensity ratios were calculated for each cell by dividing the fluorescence intensities of certain fluorophores by the fluorescence intensities of EGFP. The same gating strategies were used consistently throughout a set of experiment. Quantitative assessment and statistical analysis were performed using either R package^65^ or GraphPad Prism (version 10.2.1).

### Microscopy

Fluorescence imaging for cultured cells and primary neurons was performed on a commercial Leica Stellaris 5 confocal microscope with a supercontinuum white light laser (470–670 nm) and hybrid photodetectors for single-molecule detection. The laser power output was set to 85% of maximum power and regularly calibrated. The microscope stage was maintained in an environmental chamber (set to 37 °C, 5% CO_2_). Before imaging, the imaging plate was equilibrated on the microscope stage for 30 min to avoid thermal drifting during image acquisition. Unless specified, the following imaging settings were used: a HC PL APO CS2 ×20/0.75-NA air/water objective, 581.82 × 581.82 μm^2^, scan speed 400 MHz and *Z*-stacks with a 2-μm step size. For timelapse imaging, the settings were used: scan speed 600 MHz, 45 s per frame, *Z*-stacks with a 2-μm step size and a physical length of 10 μm. Timelapse imaging of acute mouse brain slices was performed on a LSM 710 upright microscope with a W N-Achroplan ×20/0.5-NA M27 water objective. The settings were used: 425.1 × 425.22 μm^2^, pinhole 150 μm, zoom 1.0, pixel dwell time 2.55 μs, average 1; for EGFP channel, excitation 488 nm, emission 498–550 nm; for CPY-CA channel, excitation 633 nm, emission 638–747 nm. The fluorescence imaging of fixed brain slices was performed on a Leica SP8X laser scanning confocal system with a HC PL APO CS2 ×40/1.30-NA oil objective, a supercontinuum white light laser (470–670 nm) and hybrid photodetectors. Unless specified, the settings were used: 387.5 × 387.5 μm^2^, zoom 0.75, scan speed 400 MHz, *Z*-stacks with a 2-μm step size and a physical length of 38 μm. The imaging acquisition parameters for each image are listed in Supplementary Table 8.

### Imaging processing and analysis

All images were processed and analyzed using ImageJ/Fiji (version 2.9.0/1.54h)^66^. Unless specified, the *Z*-stack images (12 or 16 bit) were first converted into maximum intensity projections. From the channel indicating Kinprola expression (mostly EGFP channel), the regions of interest were delineated manually or segmented using Cellpose 2.0 (ref. ^67^), and the mean fluorescence intensities from individual regions of interest were derived for multiple fields of view. Unhealthy neurons were excluded from analyses. For tissue imaging, background signal was determined by average mean fluorescence intensities from one to three cell-free regions and subtracted for background correction. The BRET-Analyzer (v1.0.8) plugin was employed for presenting ratiometric projections^68^. Specifically, the EGFP channel underwent thresholding (autoTh-Chastagnier method, Gaussian radius 1 and 2 using default value of 5 and 15), and the fluorescence channels were divided (for example, CPY-CA/EGFP) to generate ratiometric images.

### Data representation, reproducibility and statistical analysis

The numerical data was analyzed and plotted using the Excel (version 16.78.3), R package (version 4.3.1, 2023-06-16)^65^, OriginPro 2020b (OriginLab) and GraphPad Prism (version 10.2.1). The schemes and figures were assembled in Adobe Illustrator 2024, using some elements adopted from BioRender (https://www.biorender.com/). For fluorescence images presentation, brightness and contrast were adjusted identically for each channel. Unless specified, all in vitro measurements were performed in three technical replicates. All cell experiments were performed at least in two biological replicates. The statistical significance was determined by performing a two-tailed unpaired *t*-test with Welch’s correction or one-way analysis of variance (ANOVA) with Dunnett’s or Tukey’s post hoc test using the GraphPad Prism. The following notations apply for all statistical analyses: not significant (n.s.) *P* ≥ 0.05, *P < 0.05, **P < 0.01, ***P < 0.001 and ****P < 0.0001. The *P* values are provided for comparison.

### Reporting summary

Further information on research design is available in the Nature Portfolio Reporting Summary linked to this article.

## Online content

Any methods, additional references, Nature Portfolio reporting summaries, source data, extended data, supplementary information, acknowledgements, peer review information; details of author contributions and competing interests; and statements of data and code availability are available at 10.1038/s41589-025-01949-6.

## Supplementary information

Supplementary Information Supplementary Figs. 1–12, Tables 1–8 and Notes 1 and 2.

Reporting Summary

Supplementary Data 1 Source data the supplementary figures.

## Source data

Source Data Fig. 1 Statistical source data.

Source Data Fig. 2 Statistical source data.

Source Data Fig. 3 Statistical source data.

Source Data Fig. 4 Statistical source data.

Source Data Fig. 5 Statistical source data.

Source Data Extended Data Fig. 2 Statistical source data.

Source Data Extended Data Fig. 3 Statistical source data.

Source Data Extended Data Fig. 4 Statistical source data.

Source Data Extended Data Fig. 6 Statistical source data.

Source Data Extended Data Fig. 7 Statistical source data.

Source Data Extended Data Fig. 8 Statistical source data.

Source Data Extended Data Fig. 9 Statistical source data.

## Data Availability

All data are available in the Article or its Supplementary Information. The plasmids of interest from the study have been deposited at Addgene, and the accession codes are provided in the Supplementary Information. The GRCh38 human reference genome was downloaded under (https://www.ncbi.nlm.nih.gov/datasets/genome/GCF_000001405.26/). The raw RNA-seq data and raw sequencing data of CRISPR screen have been deposited in NCBI’s Gene Expression Omnibus^69^ and are accessible through GEO Series accession numbers GSE269419 (https://www.ncbi.nlm.nih.gov/geo/query/acc.cgi?acc=GSE269419) and GSE277987 (https://www.ncbi.nlm.nih.gov/geo/query/acc.cgi?acc=GSE277987). The reagents and materials are available from the corresponding authors upon request. Source data are provided with this paper.

## Extended data

are available for this paper at 10.1038/s41589-025-01949-6.

## References

[1] Zhang, J., Yang, P. L. & Gray, N. S. Targeting cancer with small molecule kinase inhibitors. *Nat. Rev. Cancer* **9**, 28–39 (2009). 19104514 10.1038/nrc2559 PMC12406740

[2] Taskén, K. & Aandahl, E. M. Localized effects of cAMP mediated by distinct routes of protein kinase A. *Physiol. Rev.* **84**, 137–167 (2004). 14715913 10.1152/physrev.00021.2003

[3] Yagishita, S. et al. A critical time window for dopamine actions on the structural plasticity of dendritic spines. *Science* **345**, 1616–1620 (2014). 25258080 10.1126/science.1255514 PMC4225776

[4] Yamaguchi, T. et al. Role of PKA signaling in D2 receptor-expressing neurons in the core of the nucleus accumbens in aversive learning. *Proc. Natl Acad. Sci. USA* **112**, 11383–11388 (2015). 26305972 10.1073/pnas.1514731112 PMC4568655

[5] Goto, A. et al. Circuit-dependent striatal PKA and ERK signaling underlies rapid behavioral shift in mating reaction of male mice. *Proc. Natl Acad. Sci. USA* **112**, 6718–6723 (2015). 25964359 10.1073/pnas.1507121112 PMC4450387

[6] Chen, Y. et al. Endogenous Gαq-coupled neuromodulator receptors activate protein kinase A. *Neuron* **96**, 1070–1083.e5 (2017). 29154125 10.1016/j.neuron.2017.10.023 PMC5726796

[7] Tang, S. & Yasuda, R. Imaging ERK and PKA activation in single dendritic spines during structural plasticity. *Neuron* **93**, 1315–1324.e3 (2017). 28285819 10.1016/j.neuron.2017.02.032 PMC6042854

[8] Yapo, C. et al. Detection of phasic dopamine by D1 and D2 striatal medium spiny neurons. *Physiol. J.* **595**, 7451–7475 (2017). 10.1113/JP274475 PMC5730852 28782235

[9] Ma, L. et al. A highly sensitive A-kinase activity reporter for imaging neuromodulatory events in awake mice. *Neuron* **99**, 665–679.e5 (2018). 30100256 10.1016/j.neuron.2018.07.020 PMC6152931

[10] Lee, S. J., Chen, Y., Lodder, B. & Sabatini, B. L. Monitoring behaviorally induced biochemical changes using fluorescence lifetime photometry. *Front. Neurosci.* **13**, 766 (2019). 31417343 10.3389/fnins.2019.00766 PMC6685078

[11] Lee, S. J. et al. Cell-type-specific asynchronous modulation of PKA by dopamine in learning. *Nature* **590**, 451–456 (2021). 33361810 10.1038/s41586-020-03050-5 PMC7889726

[12] Zhang, J.-F. et al. An ultrasensitive biosensor for high-resolution kinase activity imaging in awake mice. *Nat. Chem. Biol.* **17**, 39–46 (2021). 32989297 10.1038/s41589-020-00660-y PMC7773213

[13] Ma, L. et al. Locomotion activates PKA through dopamine and adenosine in striatal neurons. *Nature* **611**, 762–768 (2022). 36352228 10.1038/s41586-022-05407-4 PMC10752255

[14] Mandell, J. W. Phosphorylation state-specific antibodies. *Am. J. Pathol.* **163**, 1687–1698 (2003). 14578166 10.1016/S0002-9440(10)63525-0 PMC1892416

[15] Greenwald, E. C., Mehta, S. & Zhang, J. Genetically encoded fluorescent biosensors illuminate the spatiotemporal regulation of signaling networks. *Chem. Rev.* **118**, 11707–11794 (2018). 30550275 10.1021/acs.chemrev.8b00333 PMC7462118

[16] Lin, W. et al. Light-gated integrator for highlighting kinase activity in living cells. *Nat. Commun.* **15**, 7804 (2024). 39242543 10.1038/s41467-024-51270-4 PMC11379911

[17] Huppertz, M.-C. et al. Recording physiological history of cells with chemical labeling. *Science* **383**, 890–897 (2024). 38386755 10.1126/science.adg0812

[18] Durocher, D. et al. The molecular basis of FHA domain: phosphopeptide binding specificity and implications for phospho-dependent signaling mechanisms. *Mol. Cell* **6**, 1169–1182 (2000). 11106755 10.1016/s1097-2765(00)00114-3

[19] Pershad, K., Wypisniak, K. & Kay, B. K. Directed evolution of the forkhead-associated domain to generate anti-phosphospecific reagents by phage display. *J. Mol. Biol.* **424**, 88–103 (2012). 22985966 10.1016/j.jmb.2012.09.006 PMC3488158

[20] Adams, S. R., Harootunian, A. T., Buechler, Y. J., Taylor, S. S. & Tsien, R. Y. Fluorescence ratio imaging of cyclic AMP in single cells. *Nature* **349**, 694–697 (1991). 1847505 10.1038/349694a0

[21] Zheng, Q. et al. Rational design of fluorogenic and spontaneously blinking labels for super-resolution imaging. *ACS Cent. Sci.* **5**, 1602–1613 (2019). 31572787 10.1021/acscentsci.9b00676 PMC6764213

[22] Grimm, J. B. et al. A general method to optimize and functionalize red-shifted rhodamine dyes. *Nat. Methods* **17**, 815–821 (2020). 32719532 10.1038/s41592-020-0909-6 PMC7396317

[23] Mehta, S. et al. Single-fluorophore biosensors for sensitive and multiplexed detection of signaling activities. *Nat. Cell Biol.* **20**, 1215–1225 (2018). 30250062 10.1038/s41556-018-0200-6 PMC6258557

[24] Fosbrink, M., Aye-Han, N.-N., Cheong, R., Levchenko, A. & Zhang, J. Visualization of JNK activity dynamics with a genetically encoded fluorescent biosensor. *Proc. Natl Acad. Sci. USA* **107**, 5459–5464 (2010). 20212108 10.1073/pnas.0909671107 PMC2851826

[25] Schmitt, D. L. et al. Spatial regulation of AMPK signaling revealed by a sensitive kinase activity reporter. *Nat. Commun.* **13**, 3856 (2022). 35790710 10.1038/s41467-022-31190-x PMC9256702

[26] Weller, M. et al. Glioma. *Nat. Rev. Dis. Prim.* **1**, 15017 (2015). 27188790 10.1038/nrdp.2015.17

[27] McNamara, C. et al. 2021 WHO classification of tumours of the central nervous system: a review for the neuroradiologist. *Neuroradiology* **64**, 1919–1950 (2022). 35869291 10.1007/s00234-022-03008-6

[28] Osswald, M. et al. Brain tumour cells interconnect to a functional and resistant network. *Nature* **528**, 93–98 (2015). 26536111 10.1038/nature16071

[29] Venkataramani, V. et al. Glioblastoma hijacks neuronal mechanisms for brain invasion. *Cell* **185**, 2899–2917.e31 (2022). 35914528 10.1016/j.cell.2022.06.054

[30] Ratliff, M. et al. Individual glioblastoma cells harbor both proliferative and invasive capabilities during tumor progression. *Neuro. Oncol.* **25**, 2150–2162 (2023). 37335907 10.1093/neuonc/noad109 PMC10708941

[31] Hai, L. et al. A clinically applicable connectivity signature for glioblastoma includes the tumor network driver CHI3L1. *Nat. Commun.* **15**, 968 (2024). 38320988 10.1038/s41467-024-45067-8 PMC10847113

[32] Hausmann, D. et al. Autonomous rhythmic activity in glioma networks drives brain tumour growth. *Nature* **613**, 179–186 (2023). 36517594 10.1038/s41586-022-05520-4

[33] Kotani, S. et al. PKA and MPF-activated Polo-like kinase regulate anaphase-promoting complex activity and mitosis progression. *Mol. Cell* **1**, 371–380 (1998). 9660921 10.1016/s1097-2765(00)80037-4

[34] Vandame, P. et al. The spatio-temporal dynamics of PKA activity profile during mitosis and its correlation to chromosome segregation. *Cell Cycle* **13**, 3232–3240 (2014). 25485503 10.4161/15384101.2014.950907 PMC4615038

[35] Grieco, D., Porcellini, A., Avvedimento, E. V. & Gottesman, M. E. Requirement for cAMP-PKA pathway activation by M phase-promoting factor in the transition from mitosis to interphase. *Science* **271**, 1718–1723 (1996). 8596931 10.1126/science.271.5256.1718

[36] Henkel, L., Rauscher, B., Schmitt, B., Winter, J. & Boutros, M. Genome-scale CRISPR screening at high sensitivity with an empirically designed sgRNA library. *BMC Biol.* **18**, 174 (2020). 33228647 10.1186/s12915-020-00905-1 PMC7686728

[37] Qiao, X., Zhang, L., Gamper, A. M., Fujita, T. & Wan, Y. APC/C-Cdh1: from cell cycle to cellular differentiation and genomic integrity. *Cell Cycle* **9**, 3904–3912 (2010). 20935501 10.4161/cc.9.19.13585 PMC3047751

[38] García-Higuera, I. et al. Genomic stability and tumour suppression by the APC/C cofactor Cdh1. *Nat. Cell Biol.* **10**, 802–811 (2008). 18552834 10.1038/ncb1742

[39] Kravitz, A. V. & Kreitzer, A. C. Striatal mechanisms underlying movement, reinforcement, and punishment. *Physiology* **27**, 167–177 (2012). 22689792 10.1152/physiol.00004.2012 PMC3880226

[40] Tilden, E. I., Maduskar, A., Oldenborg, A., Sabatini, B. L. & Chen, Y. A Cre-dependent reporter mouse for quantitative real-time imaging of protein kinase A activity dynamics. *Sci. Rep.* **14**, 3054 (2024). 38321128 10.1038/s41598-024-53313-8 PMC10847463

[41] Beaulieu, J.-M. & Gainetdinov, R. R. The physiology, signaling, and pharmacology of dopamine receptors. *Pharmacol. Rev.* **63**, 182–217 (2011). 21303898 10.1124/pr.110.002642

[42] Saucerman, J. J. et al. Systems analysis of PKA-mediated phosphorylation gradients in live cardiac myocytes. *Proc. Natl Acad. Sci. USA* **103**, 12923–12928 (2006). 16905651 10.1073/pnas.0600137103 PMC1568947

[43] Francis, S. H. Structure and function of cyclic nucleotide-dependent protein kinases. *Annu. Rev. Physiol.* **56**, 237–272 (1994). 8010741 10.1146/annurev.ph.56.030194.001321

[44] Bulovaite, E. et al. A brain atlas of synapse protein lifetime across the mouse lifespan. *Neuron* **110**, 4057–4073.e8 (2022). 36202095 10.1016/j.neuron.2022.09.009 PMC9789179

[45] Mohar, B. et al. DELTA: a method for brain-wide measurement of synaptic protein turnover reveals localized plasticity during learning. *Nat. Neurosci.* **28**, 1089–1098 (2025). 40164741 10.1038/s41593-025-01923-4 PMC12081306

[46] Butkevich, A. N. et al. Fluorescent rhodamines and fluorogenic carbopyronines for super-resolution STED microscopy in living cells. *Angew. Chem. Int. Ed.* **55**, 3290–3294 (2016). 10.1002/anie.201511018 PMC4770443 26844929

[47] Grimm, J. B., Brown, T. A., Tkachuk, A. N. & Lavis, L. D. General synthetic method for Si-Fluoresceins and Si-Rhodamines. *ACS Cent. Sci.* **3**, 975–985 (2017). 28979939 10.1021/acscentsci.7b00247 PMC5620978

[48] Gibson, D. G. et al. Enzymatic assembly of DNA molecules up to several hundred kilobases. *Nat. Methods* **6**, 343–345 (2009). 19363495 10.1038/nmeth.1318

[49] Cabrita, L. D. et al. Enhancing the stability and solubility of TEV protease using in silico design. *Protein Sci.* **16**, 2360–2367 (2007). 17905838 10.1110/ps.072822507 PMC2211701

[50] Lemke, D. et al. Primary glioblastoma cultures: can profiling of stem cell markers predict radiotherapy sensitivity? *J. Neurochem.* **131**, 251–264 (2014). 24976529 10.1111/jnc.12802

[51] Zolotukhin, S. et al. Production and purification of serotype 1, 2, and 5 recombinant adeno-associated viral vectors. *Methods* **28**, 158–167 (2002). 12413414 10.1016/s1046-2023(02)00220-7

[52] Aurnhammer, C. et al. Universal real-time PCR for the detection and quantification of adeno-associated virus serotype 2-derived inverted terminal repeat sequences. *Hum. Gene Ther. Methods* **23**, 18–28 (2012). 22428977 10.1089/hgtb.2011.034

[53] Lee, J. et al. Tumor stem cells derived from glioblastomas cultured in bFGF and EGF more closely mirror the phenotype and genotype of primary tumors than do serum-cultured cell lines. *Cancer Cell* **9**, 391–403 (2006). 16697959 10.1016/j.ccr.2006.03.030

[54] Dobin, A. et al. STAR: ultrafast universal RNA-seq aligner. *Bioinformatics* **29**, 15–21 (2013). 23104886 10.1093/bioinformatics/bts635 PMC3530905

[55] Liao, Y., Smyth, G. K. & Shi, W. featureCounts: an efficient general purpose program for assigning sequence reads to genomic features. *Bioinformatics* **30**, 923–930 (2014). 24227677 10.1093/bioinformatics/btt656

[56] Frankish, A. et al. GENCODE 2021. *Nucleic Acids Res.* **49**, D916–D923 (2021). 33270111 10.1093/nar/gkaa1087 PMC7778937

[57] Love, M. I., Huber, W. & Anders, S. Moderated estimation of fold change and dispersion for RNA-seq data with DESeq2. *Genome Biol.* **15**, 550 (2014). 25516281 10.1186/s13059-014-0550-8 PMC4302049

[58] Ge, S. X., Jung, D. & Yao, R. ShinyGO: a graphical gene-set enrichment tool for animals and plants. *Bioinformatics* **36**, 2628–2629 (2020). 31882993 10.1093/bioinformatics/btz931 PMC7178415

[59] Li, W. et al. MAGeCK enables robust identification of essential genes from genome-scale CRISPR/Cas9 knockout screens. *Genome Biol.* **15**, 554 (2014). 25476604 10.1186/s13059-014-0554-4 PMC4290824

[60] Ritchie, M. E. et al. Limma powers differential expression analyses for RNA-sequencing and microarray studies. *Nucleic Acids Res.* **43**, e47 (2015). 25605792 10.1093/nar/gkv007 PMC4402510

[61] Brinkman, E. K., Chen, T., Amendola, M. & van Steensel, B. Easy quantitative assessment of genome editing by sequence trace decomposition. *Nucleic Acids Res.* **42**, e168–e168 (2014). 25300484 10.1093/nar/gku936 PMC4267669

[62] Hellweg, L. et al. A general method for the development of multicolor biosensors with large dynamic ranges. *Nat. Chem. Biol.* **19**, 1147–1157 (2023). 37291200 10.1038/s41589-023-01350-1 PMC10449634

[63] Wardill, T. J. et al. A neuron-based screening platform for optimizing genetically-encoded calcium indicators. *PLoS ONE* **8**, e77728 (2013). 24155972 10.1371/journal.pone.0077728 PMC3796516

[64] Grimm, J. B. et al. A general method to fine-tune fluorophores for live-cell and in vivo imaging. *Nat. Methods* **14**, 987–994 (2017). 28869757 10.1038/nmeth.4403 PMC5621985

[65] R Core Team. R version 4.1.2. *R Foundation for Statistical Computing* https://www.R-project.org/ (2021).

[66] Schindelin, J. et al. Fiji: an open-source platform for biological-image analysis. *Nat. Methods* **9**, 676–682 (2012). 22743772 10.1038/nmeth.2019 PMC3855844

[67] Pachitariu, M. & Stringer, C. Cellpose 2.0: how to train your own model. *Nat. Methods* **19**, 1634–1641 (2022). 36344832 10.1038/s41592-022-01663-4 PMC9718665

[68] Chastagnier, Y., Moutin, E., Hemonnot, A. L. & Perroy, J. Image processing for bioluminescence resonance energy transfer measurement-BRET-Analyzer. *Front. Comput. Neurosci.* **11**, 1–8 (2018). 10.3389/fncom.2017.00118 PMC5767221 29375357

[69] Edgar, R. Gene Expression Omnibus: NCBI gene expression and hybridization array data repository. *Nucleic Acids Res.* **30**, 207–210 (2002). 11752295 10.1093/nar/30.1.207 PMC99122

