# Supplementary material for: Molecular recording of cellular protein kinase activity with chemical labeling
Source: Nat Chem Biol. 2025 Jul 10;21(11):1818–27. doi: 10.1038/s41589-025-01949-6 (PMC12568631; doi:10.1038/s41589-025-01949-6)

nature chemical biology

Supplementary information https://doi.org/10.1038/s41589-025-01949-6

Molecular recording of cellular protein kinase activity with chemical labeling

In the format provided by the authors and unedited

Table of Contents

Supplementary Figures **2**

Supplementary Fig. 1 | Fluorescent HaloTag substrates used in this study. 2

Supplementary Fig. 2 | (related to Fig. 1) Kinprola-recorded fluorescent signal can be preserved by chemical fixation. 3

Supplementary Fig. 3 | (related to Fig. 1) Representative flow cytometry gating strategy for HEK293 cells expressing Kinprola. 4

Supplementary Fig. 4 | (related to Fig. 1) The rate of Kinprola$_{PKA}$ recording can be modulated by varying the concentration of CPY-CA. 5

Supplementary Fig. 5 | (related to Fig. 2) Representative gating and sorting strategies of Kinprola-expressing GBCs for RNA-Seq. 6

Supplementary Fig. 6 | (related to Fig. 3) GSEA comparisons of cell subpopulations selected by Kinprola$_{PKA}$ labeling during CRISPR screening. 8

Supplementary Fig. 7 | (related to Fig. 3) Corelative analysis of putative hits validation using Kinprola$_{PKA}$ labeling and an ELISA-based PKA colorimetric assay. 9

Supplementary Fig. 8 | (related to Fig. 3) Quantification of genome editing efficacy of sgRNAs used in Fig. 3d,e. 10

Supplementary Fig. 9 | (related to Fig. 3) Validation of protein knockout. 12

Supplementary Fig. 10 | (related to Fig. 4) Recording PKA activity in neurons expressing Kinprola$_{PKA}$ in the presence of varying concentrations of Iso. 13

Supplementary Fig. 11 | (related to Fig. 4) Stability of Kinprola$_{PKA}$ labeling signal in neurons over time. 14

Supplementary Fig. 12 | (related to Fig. 5) Kinprola$_{PKA}$ records neuromodulation-induced PKA activation in freely moving mice. 15

Supplementary Tables **17**

Supplementary Table 1 | Protein melting temperature measured by NanoDSF 17

Supplementary Table 2 | Biochemical characterization of Kinprola$_{PKA}$ *in vitro*. 18

Supplementary Table 3 | Reagents and resource used in the study. 19

Supplementary Table 4 | Composition of common buffers used in the study 21

Supplementary Table 5 | Plasmids and stable cell lines used in the study. 22

Supplementary Table 6 | sgRNA sequences selected from HD CRISPR sub-library A. 24

Supplementary Table 7 | Primers for amplifying the genomic regions targeted by individual sgRNAs. 25

Supplementary Table 8 | Imaging acquisition parameters for fluorescence microscopy. 26

Supplementary Note **28**

Supplementary Note 1 | Protein sequences 28

Supplementary Note 2 | Uncropped scans of all blots 31

Supplementary Figures

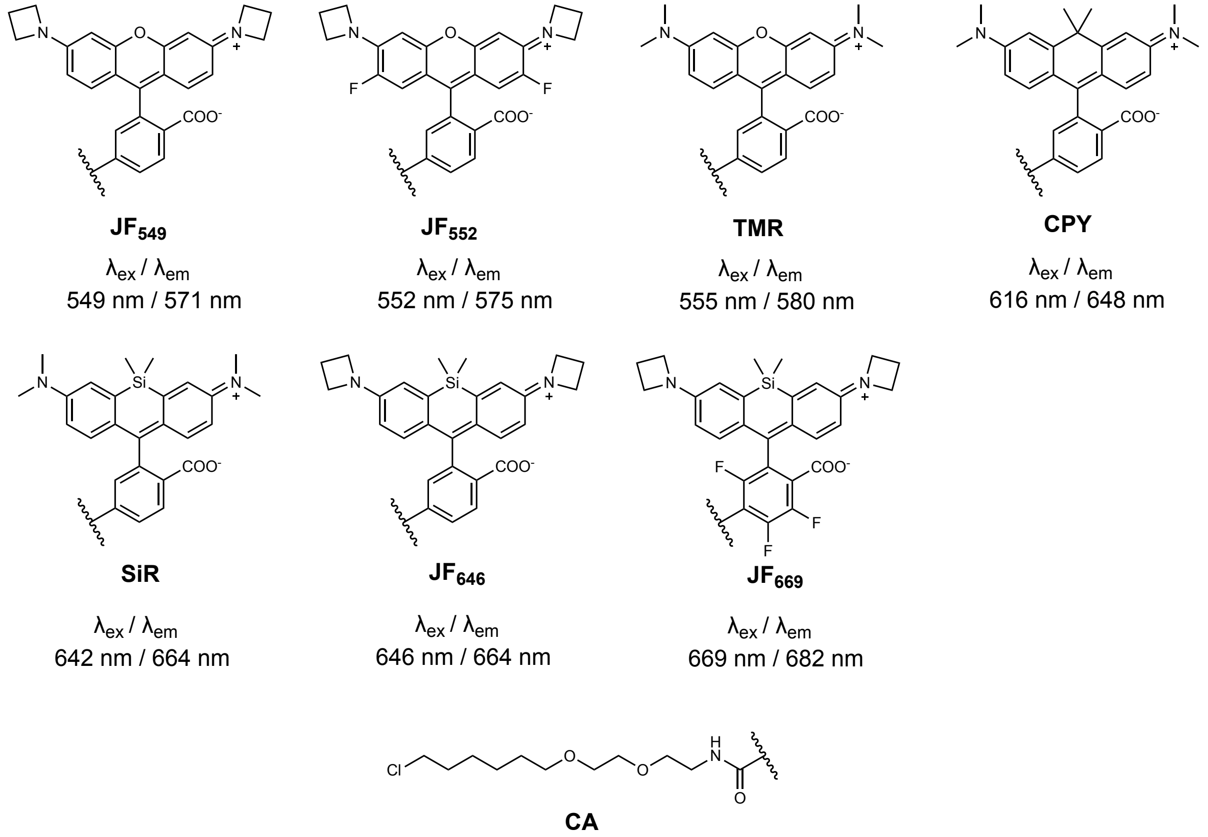

Supplementary Fig. 1 | Fluorescent HaloTag substrates used in this study. Chemical structures of fluorescent HaloTag substrates with excitation and emission maxima. Janelia Fluor (JF) fluorescent substrates were kind gifts of L. D. Lavis (Janelia Research Campus, Ashburn, Virginia). TMR-CA, CPY-CA and SiR-CA were synthesized in house according to literature procedures.

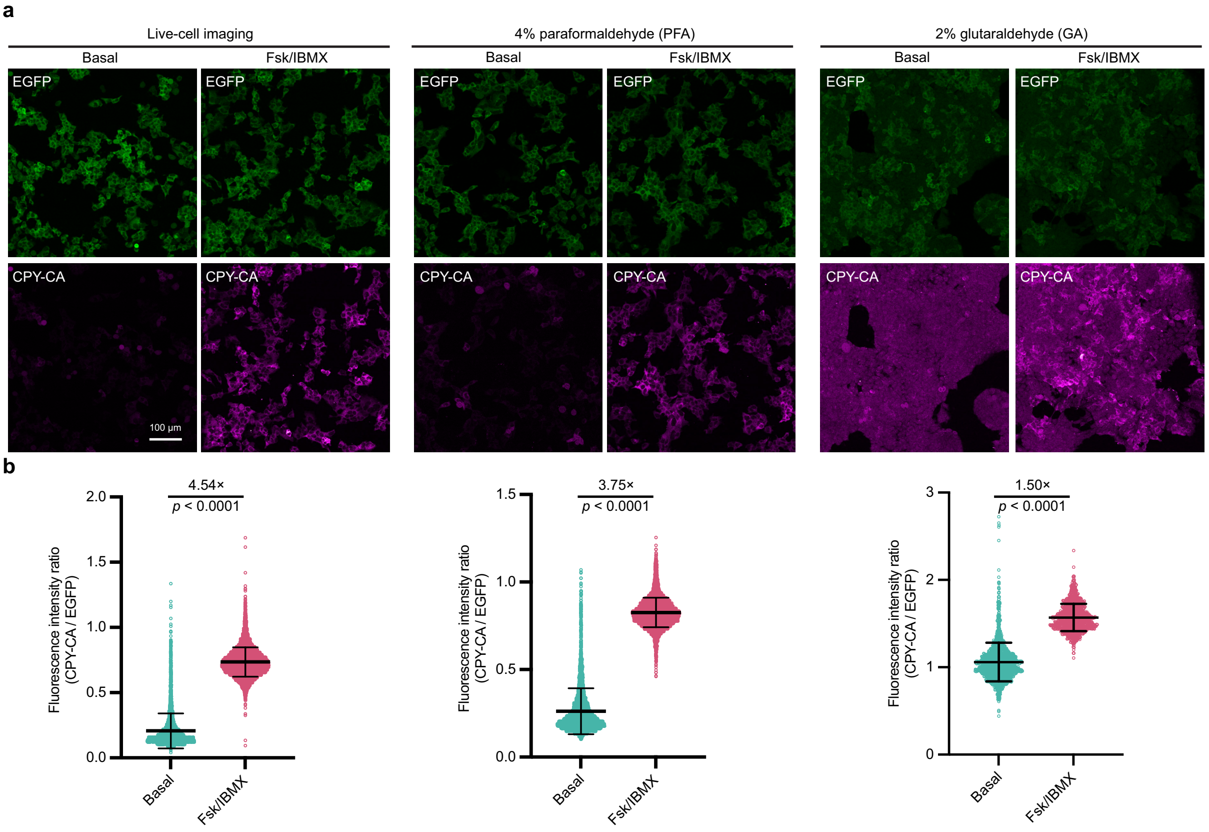

Supplementary Fig. 2 | (related to Fig. 1) Kinprola-recorded fluorescent signal can be preserved by chemical fixation. (**a**) Fluorescence images of HEK293 cells stably expressing Kinprola$_{PKA}$ labeled with 25 nM CPY-CA for 30 min in the presence or absence of 50 µM Fsk/100 µM IBMX stimulation. The cells were then fixed with 4% paraformaldehyde (PFA) or 2% glutaraldehyde (GA) and subsequently imaged. Representative images from four (live-cell imaging and 4% PFA) and two (2% GA) wells of cell culture with similar results. (**b**) Dot plots comparison of normalized fluorescence intensity from (**a**). n = 4541, 5194, 4562, 4657, 1572 and 1117 cells, collected from four (live-cell imaging and 4% PFA) and two (2% GA) cultures in one independent experiment. Error bars indicate median with interquartile range. Statistical significance was calculated with unpaired two-tailed Welch's t test; *p* < 0.0001 between basal and Fsk/IBMX group. Scale bar: 100 µm.

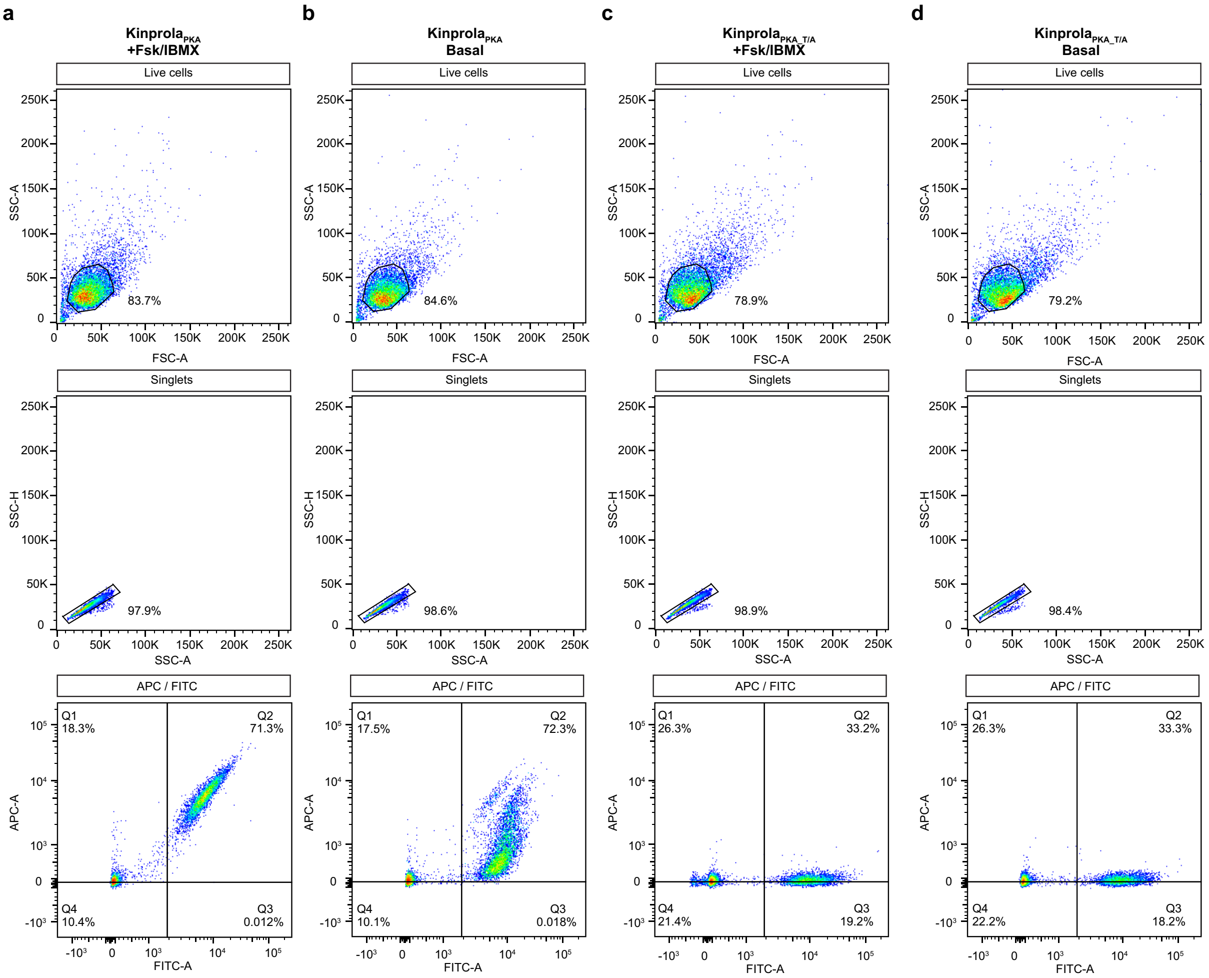

Supplementary Fig. 3 | (related to Fig. 1) Representative flow cytometry gating strategy for HEK293 cells expressing Kinprola. (**a**) Hierarchical gating of live cells (FSC-A *vs.* SSC-A), singlets (SSC-A *vs.* SSC-H) and positive cells (APC *vs.* FITC) for HEK293 cells expressing Kinprola$_{PKA}$ incubated with 25 nM CPY-CA for 30 min with 50 µM Fsk/100 µM IBMX stimulation. (**b**) Hierarchical gating for HEK293 cells expressing Kinprola$_{PKA}$ incubated with 25 nM CPY-CA for 30 min without stimulation (Basal). (**c**) Hierarchical gating for HEK293 cells expressing Kinprola$_{PKA_T/A}$ incubated with 25 nM CPY-CA for 30 min with 50 µM Fsk/100 µM IBMX stimulation. (**d**) Hierarchical gating for HEK293 cells expressing Kinprola$_{PKA_T/A}$ incubated with 25 nM CPY-CA for 30 min without stimulation (Basal).

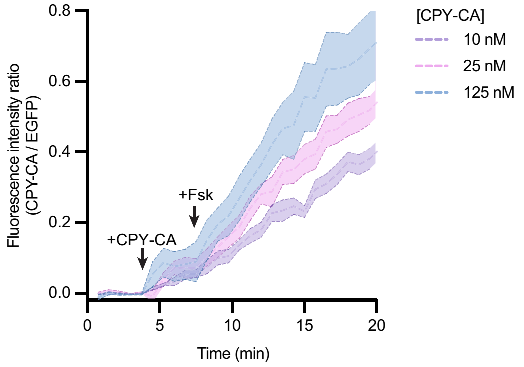

Supplementary Fig. 4 | (related to Fig. 1) The rate of Kinprola$_{PKA}$ recording can be modulated by varying the concentration of CPY-CA. Time-lapse fluorescence traces recorded on HEK293 cells stably expressing Kinprola$_{PKA}$ treated with 50 µM Fsk in the presence of different concentrations of CPY-CA. Ratio indicates CPY-CA/EGFP. Error bars indicate mean ± SD. Representative traces from two independent experiments with similar results.

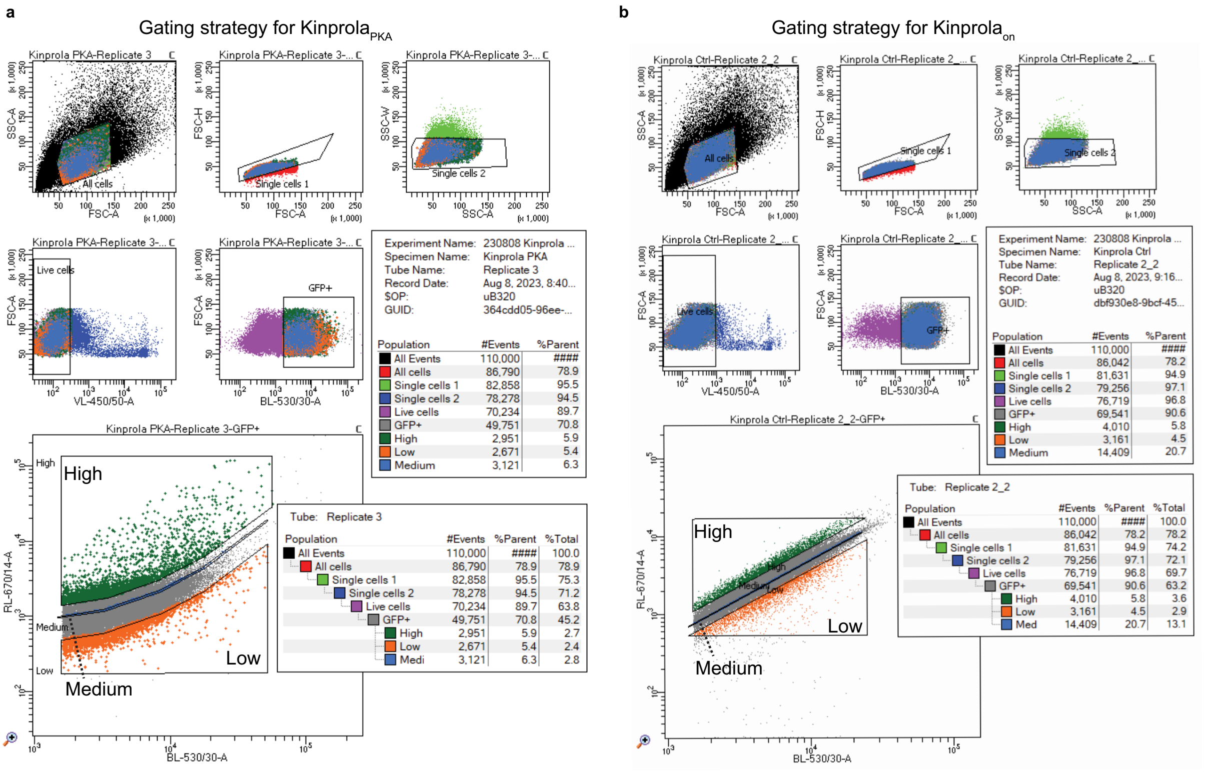

Supplementary Fig. 5 | (related to Fig. 2) Representative gating and sorting strategies of Kinprola-expressing GBCs for RNA-Seq. (**a**) Gating and sorting strategies of CPY-CA-labeled Kinprola$_{PKA}$-expressing GBCs. (**b**) Gating and sorting strategies of CPY-CA-labeled Kinprola$_{on}$-expressing GBCs.

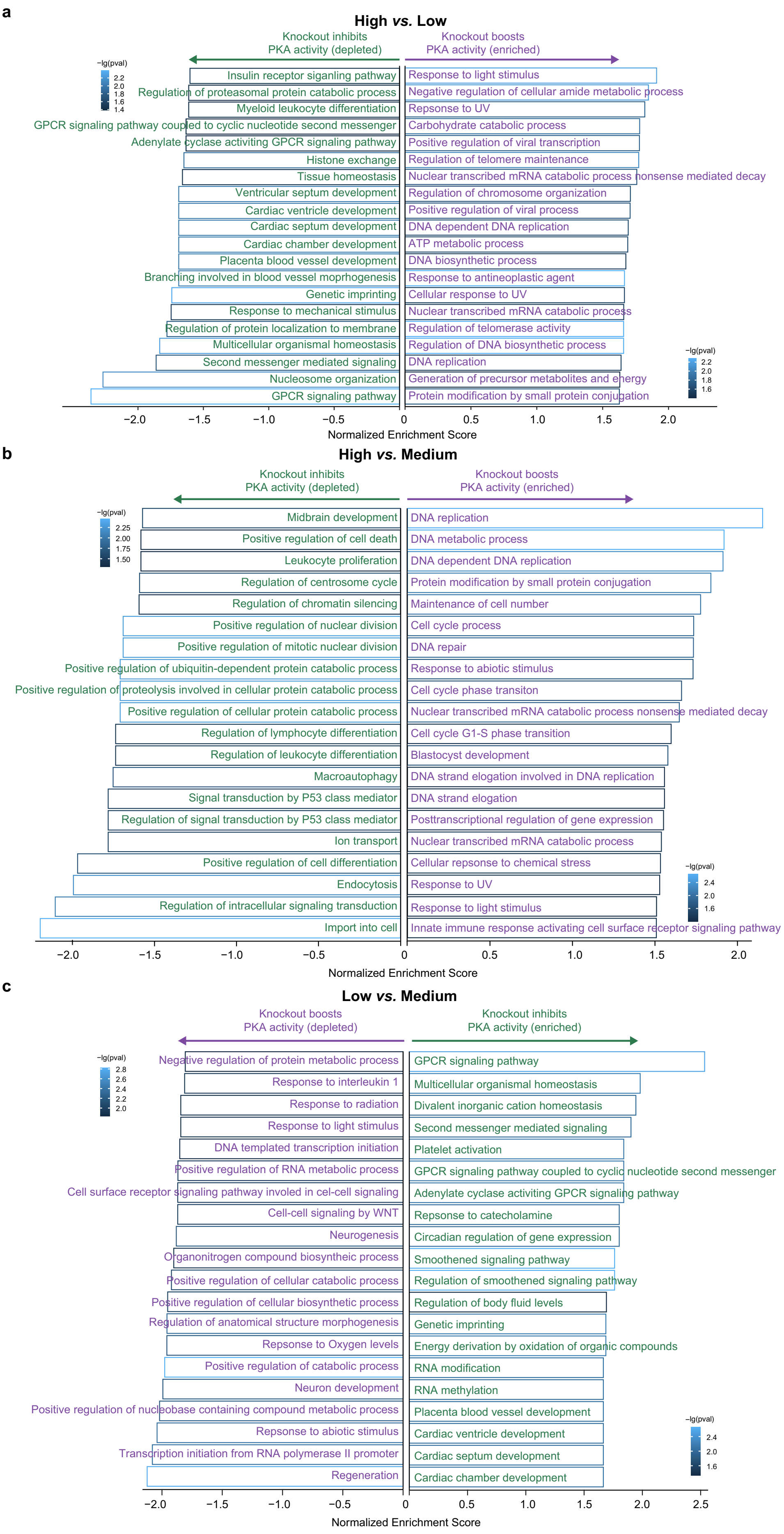
a
High vs. Low
Knockout inhibits PKA activity (depleted)
Knockout boosts PKA activity (enriched)
−lg(pval)
2.2
2.0
1.8
1.6
1.4
Insulin receptor siganling pathway
Regulation of proteasomal protein catabolic process
Myeloid leukocyte differentiation
GPCR signaling pathway coupled to cyclic nucleotide second messenger
Adenylate cyclase activiting GPCR signaling pathway
Histone exchange
Tissue homeostasis
Ventricular septum development
Cardiac ventricle development
Cardiac septum development
Cardiac chamber development
Placenta blood vessel development
Branching involved in blood vessel moprhogenesis
Genetic imprinting
Response to mechanical stimulus
Regulation of protein localization to membrane
Multicellular organismal homeostasis
Second messenger mediated signaling
Nucleosome organization
GPCR signaling pathway
Response to light stimulus
Negative regulation of cellular amide metabolic process
Repsonse to UV
Carbohydrate catabolic process
Positive regulation of viral transcription
Regulation of telomere maintenance
Nuclear transcribed mRNA catabolic process nonsense mediated decay
Regulation of chromosome organization
Positive regulation of viral process
DNA dependent DNA replication
ATP metabolic process
DNA biosynthetic process
Response to antineoplastic agent
Cellular response to UV
Nuclear transcribed mRNA catabolic process
Regulation of telomerase activity
Regulation of DNA biosynthetic process
DNA replication
Generation of precursor metabolites and energy
Protein modification by small protein conjugation
−lg(pval)
2.2
2.0
1.8
1.6
−2.0 −1.5 −1.0 −0.5 0 0.5 1.0 1.5 2.0
Normalized Enrichment Score
b
High vs. Medium
Knockout inhibits PKA activity (depleted)
Knockout boosts PKA activity (enriched)
−lg(pval)
2.25
2.00
1.75
1.50
Midbrain development
Positive regulation of cell death
Leukocyte proliferation
Regulation of centrosome cycle
Regulation of chromatin silencing
Positive regulation of nuclear division
Positive regulation of mitotic nuclear division
Positive regulation of ubiquitin-dependent protein catabolic process
Positive regulation of proteolysis involved in cellular protein catabolic process
Positive regulation of cellular protein catabolic process
Regulation of lymphocyte differentiation
Regulation of leukocyte differentiation
Macroautophagy
Signal transduction by P53 class mediator
Regulation of signal transduction by P53 class mediator
Ion transport
Positive regulation of cell differentiation
Endocytosis
Regulation of intracellular signaling transduction
Import into cell
DNA replication
DNA metabolic process
DNA dependent DNA replication
Protein modification by small protein conjugation
Maintenance of cell number
Cell cycle process
DNA repair
Response to abiotic stimulus
Cell cycle phase transiton
Nuclear transcribed mRNA catabolic process nonsense mediated decay
Cell cycle G1-S phase transition
Blastocyst development
DNA strand elogation involved in DNA replication
DNA strand elogation
Posttranscriptional regulation of gene expression
Nuclear transcribed mRNA catabolic process
Cellular repsonse to chemical stress
Response to UV
Repsonse to light stimulus
Innate immune response activating cell surface receptor signaling pathway
−lg(pval)
2.4
2.0
1.6
−2.0 −1.5 −1.0 −0.5 0 0.5 1.0 1.5 2.0
Normalized Enrichment Score
c
Low vs. Medium
Knockout boosts PKA activity (depleted)
Knockout inhibits PKA activity (enriched)
−lg(pval)
2.8
2.6
2.4
2.2
2.0
Negative regulation of protein metabolic process
Response to interleukin 1
Response to radiation
Response to light stimulus
DNA templated transcription initiation
Positive regulation of RNA metabolic process
Cell surface receptor signaling pathway involed in cel-cell signaling
Cell-cell signaling by WNT
Neurogenesis
Organonitrogen compound biosyntheic process
Positive regulation of cellular catabolic process
Positive regulation of cellular biosynthetic process
Regulation of anatomical structure morphogenesis
Repsonse to Oxygen levels
Positive regulation of catabolic process
Neuron development
Positive regulation of nucleobase containing compound metabolic process
Repsonse to abiotic stimulus
Transcription initiation from RNA polymerase II promoter
Regeneration
GPCR signaling pathway
Multicellular organismal homeostasis
Divalent inorganic cation homeostasis
Second messenger mediated signaling
Platelet activation
GPCR signaling pathway coupled to cyclic nucleotide second messenger
Adenylate cyclase activiting GPCR signaling pathway
Repsonse to catecholamine
Circadian regulation of gene expression
Smoothened signaling pathway
Regulation of smoothened signaling pathway
Regulation of body fluid levels
Genetic imprinting
Energy derivation by oxidation of organic compounds
RNA modification
RNA methylation
Placenta blood vessel development
Cardiac ventricle development
Cardiac septum development
Cardiac chamber development
−lg(pval)
2.4
2.0
1.6
−2.0 −1.5 −1.0 −0.5 0 0.5 1.0 1.5 2.0 2.5
Normalized Enrichment Score

Supplementary Fig. 6 | (related to Fig. 3) GSEA comparisons of cell subpopulations selected by Kinprola$_{PKA}$ labeling during CRISPR screening. (a-c) GSEA top 20 categories among "high" *vs.* "low", "high" *vs.* "medium" and "low" *vs.* "medium" comparisons. Bar graphs are color-coded by *p* values.

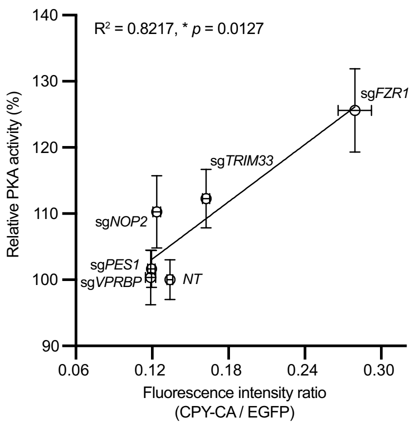

Supplementary Fig. 7 | (related to Fig. 3) Corelative analysis of putative hits validation using Kinprola$_{PKA}$ labeling and an ELISA-based PKA colorimetric assay. The data were replotted from **Fig. 3d**,**e**, and fitted with a simple linear regression model. The Pearson R-squared value and *p* value are provided. Error bars indicate mean ± SEM.

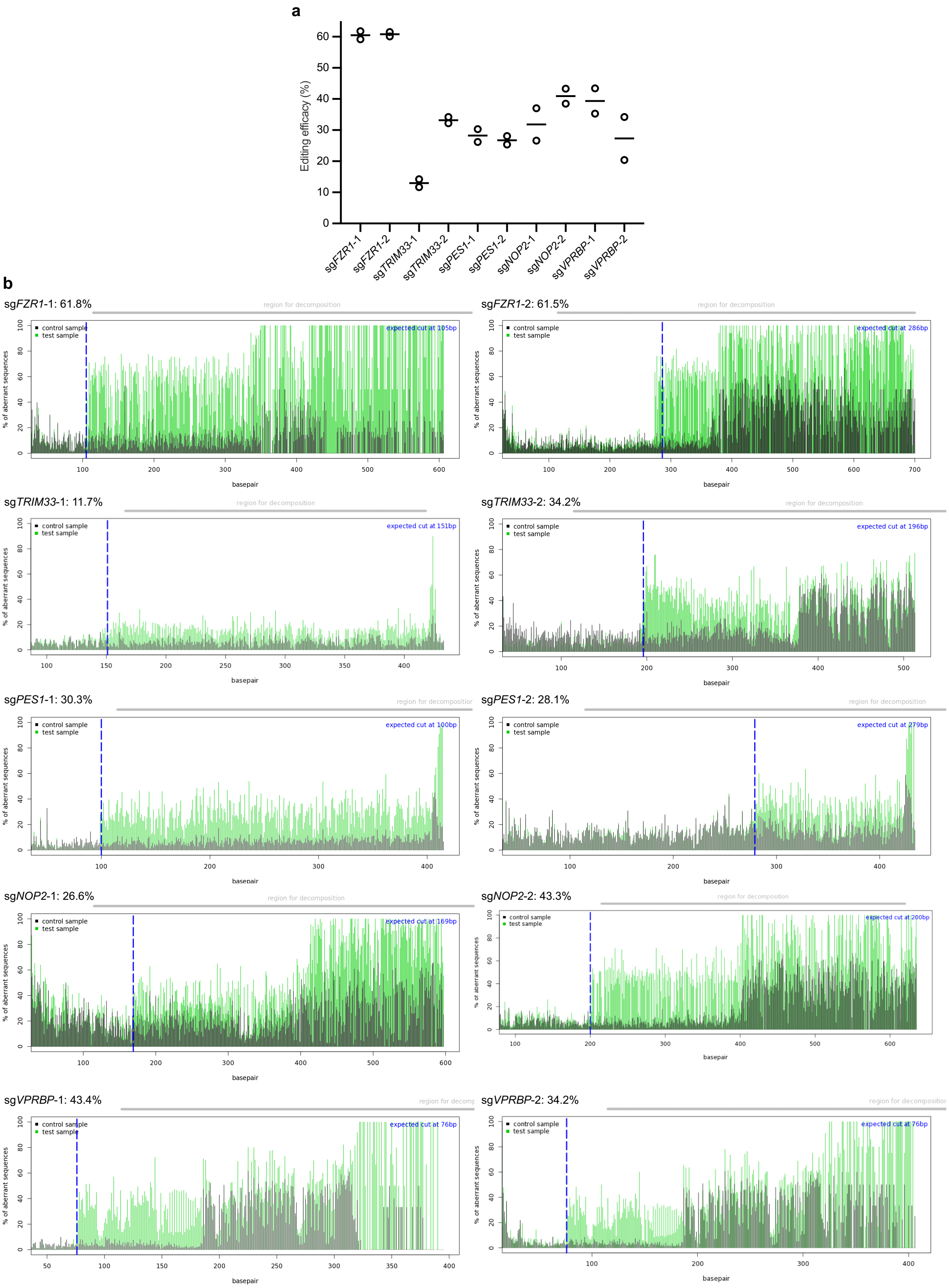

Supplementary Fig. 8 | (related to Fig. 3) Quantification of genome editing efficacy of sgRNAs used in Fig. 3d,e. (**a**) Genome editing efficacy of sgRNAs was quantified using TIDE (Tracking of Indels by Decomposition). Genomic DNA was isolated from cells expressing individual sgRNAs or a non-targeting (NT) control. Fragments flanking the sgRNA target sites were amplified by PCR, purified by gel extraction, and sequenced via Sanger sequencing. Chromatogram sequence files from edited

samples and the NT control were aligned and indel frequencies were analyzed using TIDE. Data from two independent experiments, with central lines indicating the average. (**b**) Chromatogram sequence alignments of edited samples (individual sgRNAs) and the NT control. Representative alignments from two independent experiments with similar results.

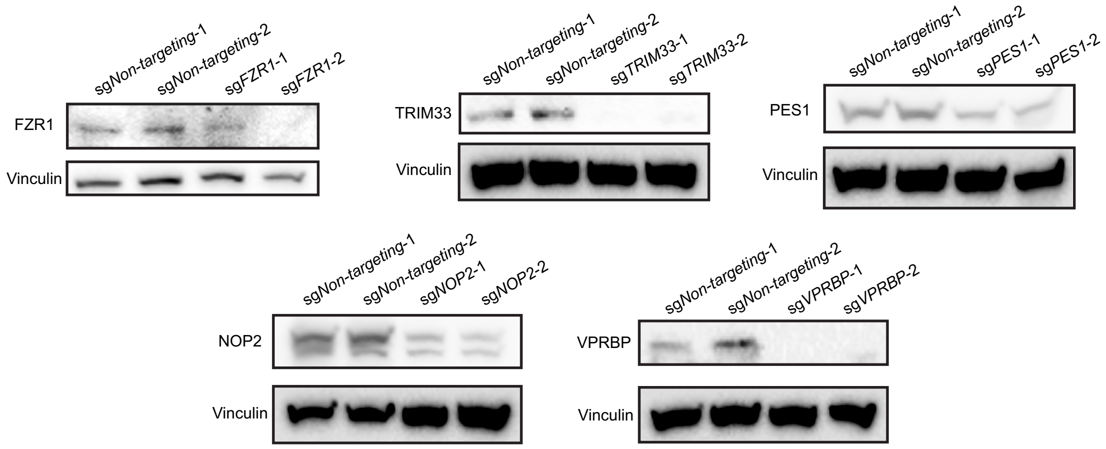

Supplementary Fig. 9 | (related to Fig. 3) Validation of protein knockout. RKO cells expressing Kinprola$_{PKA}$ and Cas9 were transfected with individual sgRNAs and subjected to puromycin selection as described in **Fig. 3** and methods section. Following selection, cells were lysed, and protein knockout level was confirmed by Western blot analysis. Data from one independent experiment.

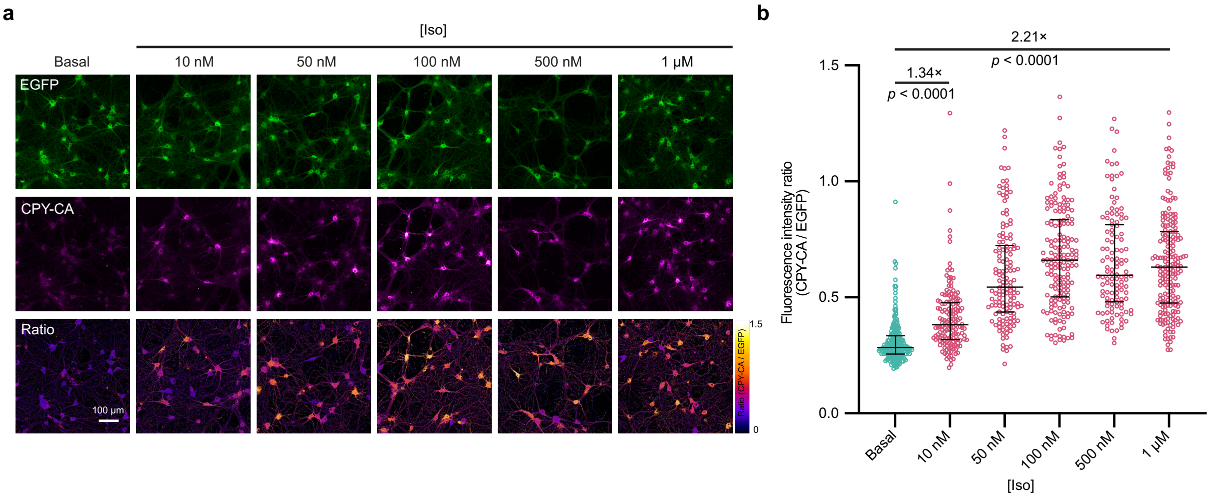

Supplementary Fig. 10 | (related to Fig. 4) Recording PKA activity in neurons expressing Kinprola$_{PKA}$ in the presence of varying concentrations of Iso. (**a**) Fluorescence images of primary rat hippocampal neurons expressing Kinprola$_{PKA}$ labeled with CPY-CA (125 nM, 25 min) in the presence of different concentrations of Iso. Neurons were first pre-incubated with CPY-CA for 5 min before Iso stimulation. Representative images from two wells of neuron culture with similar results. (**b**) Quantification of Kinprola$_{PKA}$ labeling from (**a**). n = 201, 155, 140, 170, 120 and 199 neurons, collected from six fields of view across two cultures in one independent experiment. Error bars indicate median with interquartile range. Statistical significance was calculated with one-way ANOVA with Dunnett's Post hoc test and *p* values are given for comparison. Scale bar: 100 μm.

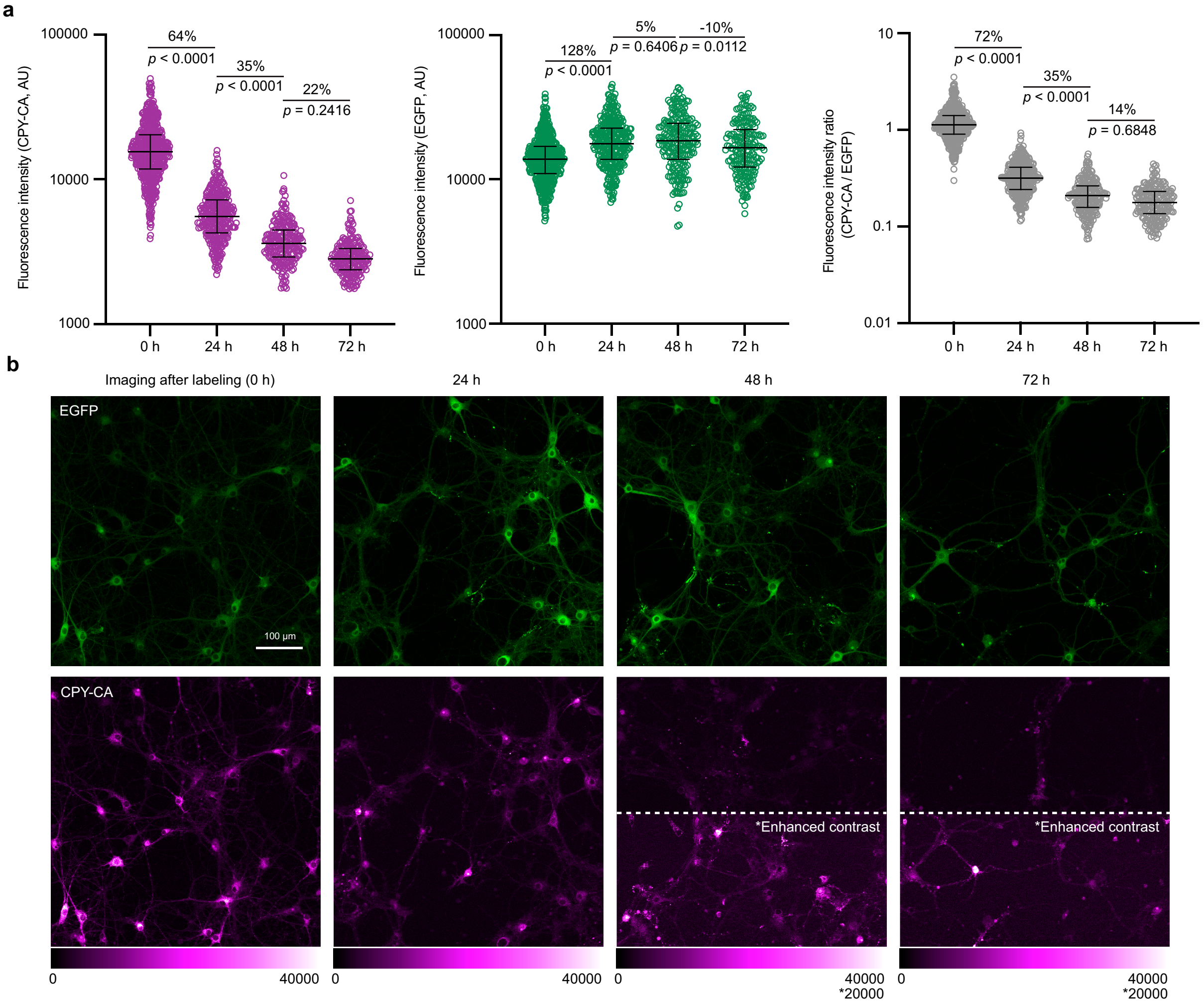

Supplementary Fig. 11 | (related to Fig. 4) Stability of Kinprola$_{PKA}$ labeling signal in neurons over time. (**a**) Dot plots showing the changes in fluorescence intensities (EGFP and CPY-CA) and fluorescence intensity ratios (CPY-CA/EGFP) of Kinprola$_{PKA}$-expressing primary hippocampal neurons over time. Neurons were labeled with 125 nM CPY-CA for 1 h without external stimulation. Images were acquired immediately after labeling (0 h) and subsequently every 24 h over a period of 3 days under live conditions. Error bars indicate median with interquartile range. n = 559, 335, 234 and 205 neurons per group from three 24-well plate cultures in one independent experiment. Statistical significance was calculated with one-way ANOVA with Tukey's Post hoc test and *p* values are given for comparison. (**b**) Representative images from the experiments described in (**a**). The contrast of images acquired at 48 h and 72 h was enhanced (CPY-CA channel, bottom half) to highlight that Kinprola$_{PKA}$ labeling can still be reliably detected after an extended period. Scale bar: 100 µm.

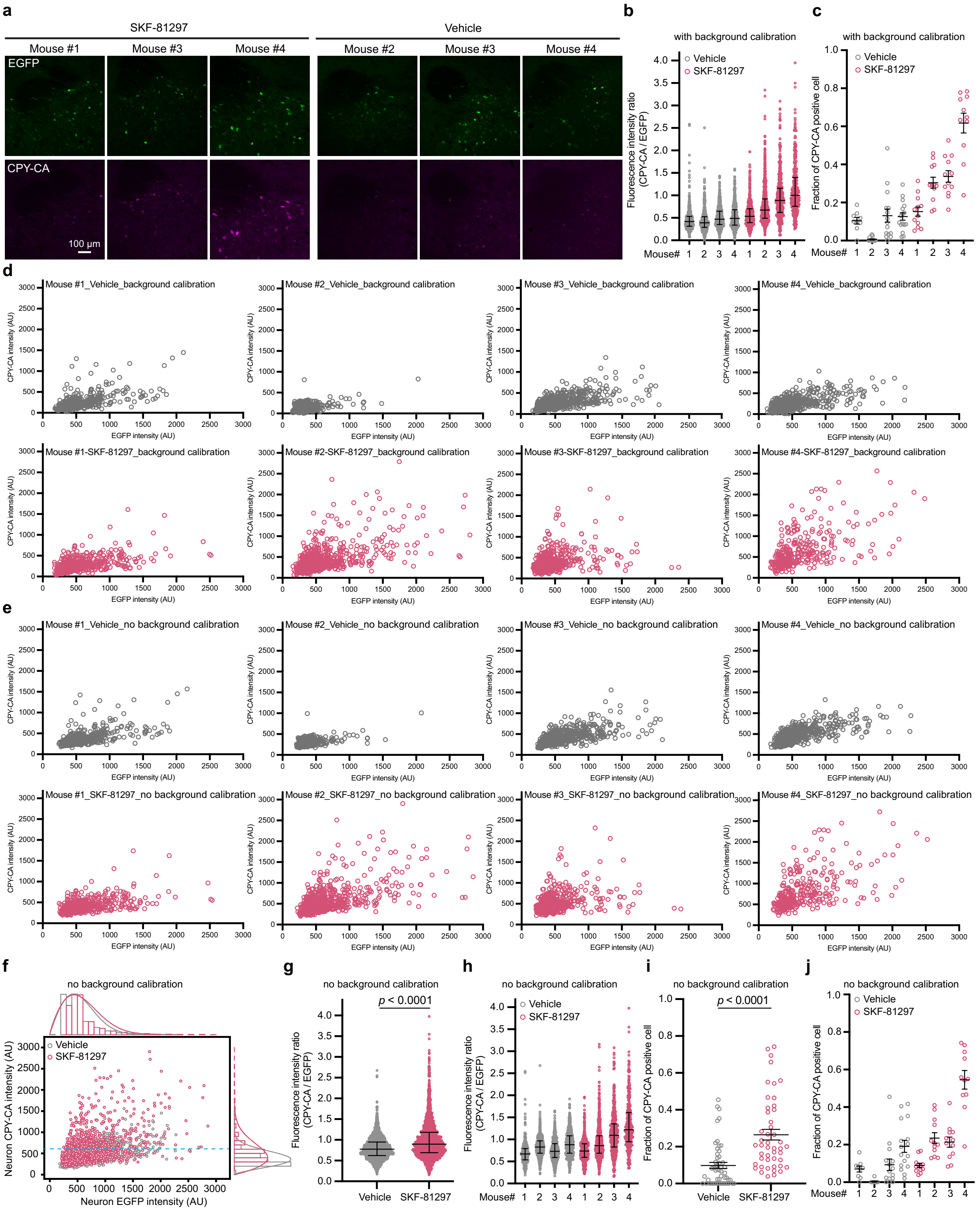

Supplementary Fig. 12 | (related to Fig. 5) Kinprola$_{PKA}$ records neuromodulation-induced PKA activation in freely moving mice. (**a**) Additional representative images of the NAc expressing Kinprola$_{PKA}$ labeled with CPY-CA from 3 mice injected with SKF-81297 or vehicle. Scale bar: 100 μm. (**b**) Dot plot of CPY-CA/EGFP ratios of neurons per mouse from **Fig. 5j**. (**c**) Fraction of EGFP positive neurons with CPY-CA above vehicle 90th percentile threshold per slices per mouse from **Fig. 5k**. (**d**) Scatter plot of CPY-CA *vs.* EGFP fluorescence for individual neurons per mouse from **Fig. 5i**. AU, arbitrary units. (**e**,**f**) Same as (**d**) but with raw intensity values, shown for single mouse (**e**) or pooled (**f**). The horizontal dashed line indicates the 90th percentile threshold of CPY-CA fluorescence in vehicle-treated neurons. (**g**) Dot plot of CPY-CA/EGFP ratios from (**f**). (**h**) Same as (**g**), plotted per mouse. (**i**)

Fraction of CPY-CA positive neurons (threshold from **f**), pooled data. (**j**) Same as (**i**), plotted per mouse. Error bars indicate median with interquartile range (**b**,**g**,**h**) or mean ± SEM (**c**,**i**,**j**). n = 2512 neurons/47 slices/4 mice/SKF-81297 group; 2593/52/4/vehicle group from two independent experiments (**a**-**j**). Statistical significance was calculated with unpaired two-tailed Welch's *t* test (**g**,**i**); $p < 0.0001$ between vehicle and SKF-81297 group (**g**,**i**).

Supplementary Tables

Supplementary Table 1 | Protein melting temperature measured by NanoDSF.

Purified protein	Average melting temperature (°C)
Kinprola$_{PKA}$	45.3
Phosphorated Kinprola$_{PKA}$	49.8
Kinprola$_{PKA_T/A}$	44.8
Kinprola$_{on}$	45.8
FHA1	52.3
cpFHA1	37.2
cpFHA1^{N49Y}	43.8

Supplementary Table 2 | Biochemical characterization of Kinprola$_{PKA}$ *in vitro*.

**Fluorescent HaloTag substrates**	**Kinprola$_{PKA}$ $k_{PKAcat+ATP}$ ($M^{-1}s^{-1}$)**	**Kinprola$_{PKA}$ k_{buffer} ($M^{-1}s^{-1}$)**	**$k_{PKAcat+ATP}$ / k_{buffer}**
CPY-CA	2.56×10^5 ($2.54 - 2.58 \times 10^5$)	54.68 (54.55 - 54.80)	4682 (4656 - 4708)
TMR-CA	1.37×10^5 ($1.36 - 1.38 \times 10^5$)	63.48 (63.37 - 63.60)	2158 (2146 - 2170)
JF_{549}-CA	7.32×10^4 ($7.26 - 7.38 \times 10^4$)	60.90 (60.79 - 61.00)	1202 (1194 - 1210)
JF_{552}-CA	5.46×10^4 ($5.44 - 5.49 \times 10^4$)	57.02 (56.92 - 57.12)	958 (956 - 961)
JF_{669}-CA	3.84×10^4 ($3.81 - 3.87 \times 10^4$)	136.74 (136.32 - 137.16)	281 (279 - 282)

Supplementary Table 3 | Reagents and resource used in the study.

Reagents	Source	Identifier
E.coli strain 10G	Lucigen	60108
E. coli strain BL21(DE3)	Novagen	69451
NEB stable competent *E. coli*	NEB	C3040H
One Shot Stbl3 chemically competent *E. coli*	ThermoFisher Scientific	C737303
Q5 high-fidelity DNA polymerase	NEB	M0491S
KOD-hot-start DNA polymerase master mix	Sigma-Aldrich	71086
Q5 site-directed mutagenesis kit	NEB	E0554S
In-Fusion Cloning	Takara Bio	639650
NucleoSpin Gel and PCR Clean-up, Mini kit	Macherey&Nagel	740609.50
QIAquick PCR Purification Kit	QIAGEN	28106
QIAprep Spin Miniprep Kit	QIAGEN	27104
GeneJET Endo-Free Plasmid Maxiprep Kit	ThermoFisher Scientific	K0861
Quick Ligation Kit	NEB	M2200S
Ultra-15 Centrifugal Filter Unit	Amicon	UFC901024D
Quick CIP	NEB	M0525S
BsrGI-HF	NEB	R3575S
BfuAI	NEB	R0701S
Isopropyl-β-D-thiogalactopyranoside (IPTG)	Carl Roth	CN084
Phenylmethylsulfonyl fluoride (PMSF)	ThermoFisher Scientific	36978
Lysozyme	ThermoFisher Scientific	89833
HisPur Ni-NTA Superflow Agarose	Thermo Scientific	25217
DMEM high glucose + GlutaMAX	Gibco	31966021
DMEM F12	Gibco	11330-032
DMEM high glucose, phenol red-free	Gibco	31053-028
RPMI + GlutaMAX-I	Gibco	61870-036
Neurobasal medium	Gibco	12348-017
PBS pH 7.4	Gibco	10010-015
TrypLE Express Enzyme	Gibco	12604-013
Accutase	Gibco	A1110501
Opti-MEM	Gibco	31985-054
GlutaMAX	Gibco	35050061
B-27	Gibco	17504044
B-27 Supplement, minus vitamin A	Gibco	12587-010
Insulin	Sigma-Aldrich	I9278
Heparin	Sigma-Aldrich	H4784
Human EGF Recombinant Protein	Gibco	PHG0311
Human FGF-basic (FGF-2/bFGF) (aa 10-155) Recombinant Protein	Gibco	PHG0021
Penicillin-Streptomycin (Pen/Strep)	Gibco	15140122
Blasticidin	InvivoGen	ant-bl-1
Puromycin	Biomol	Cay13884
Hygromycin B	Carl Roth	250-545-5
Polybrene	Sigma-Aldrich	TR-1003-G
Poly-D-lysine	Sigma-Aldrich	A-003-E
Lipofectamine 3000 reagent	ThermoFisher Scientific	L3000015
TransIT-LT1 transfection reagent	VWR	731-0027
jetOPTIMUS transfection reagent	Polyplus	101000006
Methanol-free formaldehyde	ThermoFisher Scientific	28908
DL-2-Amino-5-phosphonovaleric acid (APV)	Sigma-Aldrich	A5282
NBQX disodium salt	Abcam	ab120046
Forskolin (Fsk)	TCI	F0855
H-89 dihydrochloride hydrate	Sigma-Aldrich	B1427

3-Isobutyl-1-methylxanthine (IBMX)	Alfa Aesar	J64598
(−)-Isoproterenol hydrochloride (Iso)	Sigma-Aldrich	I6504
N6,2'-O-Dibutyryladenosine 3',5'-cyclic monophosphate sodium salt (Bt_2cAMP)	Sigma-Aldrich	D0627
(−)-Epinephrine (Epi)	Sigma-Aldrich	E4250
L-(−)-Norepinephrine (+)-bitartrate salt monohydrate	Sigma-Aldrich	A9512
(±)-Propranolol hydrochloride	Sigma-Aldrich	40543
Prostaglandin E1 (PGE1)	Avanti Neutral Lipids	900100P
Rolipram (Rol)	TCI	R0110
2-Deoxy-D-glucose (2-DG)	Sigma-Aldrich	D6134
Ionomycin	Abcam	Ab120116
SBI-0206965	Sigma-Aldrich	SML1540
Thapsigargin	ThermoFisher Scientific	T7458
Anisomycin	Sigma-Aldrich	A9789
JNK Inhibitor VIII	Sigma-Aldrich	420135
Phorbol 12-myristate 13-acetate (PMA)	Sigma-Aldrich	P1585
Gö 6983	Sigma-Aldrich	G1918
Adenosine 5'-triphosphate magnesium salt	Sigma-Aldrich	A9187
Fluoromount-G	SouthernBiotech	0100-01
Pluronic F-127	ThermoFisher Scientific	P3000MP
SKF-81297 hydrobromide	MCE	HY-12236
0.45 µm PES membrane	Millipore	HPWP04700
Falcon 875cm² Rectangular Straight Neck Cell Culture Multi-Flask, 5-layer with Vented Cap	Corning	353144
Arcturus PicoPure Frozen RNA Isolation Kit	ThermoFisher Scientific	KIT0204
RNase-Free DNase Set	Qiagen	79254
Kapa HiFi HS RM (6.25 mL)	Roche	07958935001
KAPA NGS Library Quantification Kit - Illumina for 480 Light Cycler	Roche	07960298001
DNA High Sensitivity Kit	Agilent	5067-4626
QIAamp DNA Blood Maxi Kit	Qiagen	51192
QIAquick Gel Extraction Kit	Qiagen	12578
Protease inhibitor cocktail	Sigma-Aldrich	P1860
Phosphatase inhibitor cocktail	Sigma-Aldrich	P5726
SYTOX blue dead cell stain	ThermoFisher Scientific	S34857
Pierce BCA Protein Assay Kit	ThermoFisher Scientific	23225
PKA Colorimetric Activity Kit	ThermoFisher Scientific	EIAPKA

Supplementary Table 4 | Composition of common buffers used in the study.

Buffer name	Composition
Activity buffer	50 mM HEPES, 50 mM NaCl, pH 7.3
Kinase assay buffer	50 mM Tris-HCl, 10 mM $MgCl_2$, 0.1 mM EDTA, 2 mM DTT, 0.5 mg mL^{-1} BSA, pH 7.5
IMAC lysis buffer	50 mM KH_2PO_4, 150 mM NaCl, 5 mM imidazole, 1 mM PMSF,0.25 mg mL^{-1} lysozyme, pH 8.0
IMAC wash buffer	50 mM KH_2PO_4, 300 mM NaCl, 10 mM imidazole, pH 7.5
IMAC elution buffer	50 mM KH_2PO_4, 300 mM NaCl, 500 mM imidazole, pH 7.5
TNT extraction buffer	20 mM Tris, pH 7.5, 150 mM NaCl, 1% (vol/vol) Triton X-100,10 mM $MgCl_2$
Activated cell lysis buffer	Cell lysis buffer provided by PKA Colorimetric Activity Kit, with 0.1% (vol/vol) protease inhibitor cocktail, 1 mM PMSF, 1% (vol/vol) phosphatase inhibitor cocktail
Slicing buffer	110 mM Choline-Cl, 2.5 mM KCl, 7 mM $MgCl_2$, 1 mM NaH_2PO_4, 0.5 mM $CaCl_2$, 25 mM $NaHCO_3$, 25 mM glucose, pH 7.4
Artificial cerebrospinal fluid (ACSF)	125 mM NaCl, 2.5 mM KCl, 1.3 mM $MgCl_2$, 1 mM NaH_2PO_4, 2 mM $CaCl_2$, 25 mM $NaHCO_3$, 25 mM glucose, pH 7.4

Supplementary Table 5 | Plasmids and stable cell lines used in the study.

Construct	Purpose	Addgene #	Gene of interest	Stable cell line
PKAcat	Expressing PKA catalytic subunit alpha	#14921, gift from Susan Taylor	PKA catalytic subunit alpha	n.a.
pMD2.G	VSV-G envelope expressing plasmid	#12259, gift from Didier Trono	VSV G	n.a.
psPAX2	2nd generation lentiviral packaging plasmid	#12260, gift from Didier Trono	n.a.	n.a.
pET-51b(+) FHA1	FHA1 protein production in *E .coli*	n.a.	FHA1	n.a.
pET-51b(+) cpFHA1	cpFHA1 protein production in *E .coli*	n.a.	cpFHA1	n.a.
pET-51b(+) cpFHA1^{N49Y}	cpFHA1^{N49Y} protein production in *E .coli*	n.a.	cpFHA1^{N49Y}	n.a.
pET-51b(+)-His10_TEVsite_NES-Kinprola$_{PKA}$-mEGFP	Kinprola$_{PKA}$ protein production in *E .coli*	n.a.	NES-Kinprola$_{PKA}$-mEGFP	n.a.
pET-51b(+)-His10_TEVsite_NES-Kinprola$_{PKA\ T/A}$-mEGFP	Kinprola$_{PKA\ T/A}$ protein production in *E .coli*	n.a.	NES-Kinprola$_{PKA\ T/A}$-mEGFP	n.a.
pET-51b(+)-His10_TEVsite_NES-Kinprola$_{on}$-mEGFP	Kinprola$_{PKA}$ protein production in *E .coli*	n.a.	NES-Kinprola$_{on}$-mEGFP	n.a.
pCDNA5/FRT_CMV_NES-Kinprola$_{PKA}$-mEGFP	Mammalian cell expression of Kinprola$_{PKA}$ in cytosol	#233353	NES-Kinprola$_{PKA}$-mEGFP	HEK293, HeLa
pCDNA5/FRT_CMV_NES-Kinprola$_{PKA_T/A}$-mEGFP	Mammalian cell expression of Kinprola$_{PKA_T/A}$ in cytosol	#233354	NES-Kinprola$_{PKA_T/A}$-mEGFP	HEK293, HeLa
pCDNA5/FRT_CMV_NES-Kinprola$_{on}$-mEGFP	Mammalian cell expression of Kinprola posCTRL in cytosol	#233355	NES-Kinprola$_{on}$-mEGFP	HEK293, HeLa
pCDNA5/FRT_CMV_NES-Kinprola$_{off}$-mEGFP	Mammalian cell expression of Kinprola negCTRL in cytosol	#233356	NES-Kinprola$_{off}$-mEGFP	HEK293, HeLa
pCDNA5/FRT_CMV_NES-Kinprola$_{PKC}$-mEGFP	Mammalian cell expression of Kinprola$_{PKC}$ in cytosol	#233357	NES-Kinprola$_{PKC}$-mEGFP	n.a.
pCDNA5/FRT_CMV_NES-Kinprola$_{PKC_T/A}$-mEGFP	Mammalian cell expression of Kinprola$_{PKC_T/A}$ in cytosol	#233358	NES-Kinprola$_{PKC_T/A}$-mEGFP	n.a.
pCDNA5/FRT_CMV_NES-Kinprola$_{JNK}$-mEGFP	Mammalian cell expression of Kinprola$_{JNK}$ in cytosol	#233359	NES-Kinprola$_{JNK}$-mEGFP	n.a.
pCDNA5/FRT_CMV_NES-Kinprola$_{JNK_T/A}$-mEGFP	Mammalian cell expression of Kinprola$_{JNK_T/A}$ in cytosol	#233360	NES-Kinprola$_{JNK_T/A}$-mEGFP	n.a.
pCDNA5/FRT_CMV_NES-Kinprola$_{AMPK}$-mEGFP	Mammalian cell expression of Kinprola$_{AMPK}$ in cytosol	#233361	NES-Kinprola$_{AMPK}$-mEGFP	n.a.
pCDNA5/FRT_CMV_NES-Kinprola$_{AMPK_T/A}$-mEGFP	Mammalian cell expression of Kinprola$_{AMPK_T/A}$ in cytosol	#233362	NES-Kinprola$_{AMPK_T/A}$-mEGFP	n.a.
pCDNA5/FRT_CMV_Kinprola$_{PKA}$-mEGFP-MTBD	Mammalian cell expression of Kinprola$_{PKA}$ targeted to microtubule	#233363	Kinprola$_{PKA}$-mEGFP-MTBD	HEK293
pAAV_CAG_NES-Kinprola$_{PKA}$-mTagBFP2_WPRE-SV40	Mammalian cell expression of Kinprola$_{PKA}$ in cytosol	#233364	NES-Kinprola$_{PKA}$-mTagBFP2	n.a.
pAAV_CAG_Kinprola$_{PKA}$-mEGFP-NLS3×_WPRE-SV40	Mammalian cell expression of Kinprola$_{PKA}$ in nucleus	#233365	Kinprola$_{PKA}$-mEGFP-NLS3×	n.a.
pAAV_hSyn_NES-Kinprola$_{PKA}$-mEGFP_WRPE-SV40	Neuronal expression of Kinprola$_{PKA}$ in cytosol	#233366	NES-Kinprola$_{PKA}$-mEGFP	n.a.
pAAV_hSyn_NES-Kinprola$_{PKA\ T/A}$-mEGFP_WRPE-SV40	Neuronal expression of Kinprola$_{PKA\ T/A}$ in cytosol	#233367	NES-Kinprola$_{PKA\ T/A}$-mEGFP	n.a.
pAAV_hSyn_NES-Kinprola$_{on}$-mEGFP_WRPE-SV40	Neuronal expression of Kinprola$_{on}$ in cytosol	#233368	NES-Kinprola$_{on}$-mEGFP	n.a.
pLKO.1-puro_CMV_NES-Kinprola$_{PKA}$-mEGFP	Glioblastoma cell expression of Kinprola$_{PKA}$ in cytosol	#233369	NES-Kinprola$_{PKA}$-mEGFP	GBC S24
pLKO.1-puro_CMV_NES-Kinprola$_{on}$-mEGFP	Glioblastoma cell expression of Kinprola$_{on}$ in cytosol	#233370	NES-Kinprola$_{on}$-mEGFP	GBC S24
pLenti_EF1α-NES-Kinprola$_{PKA}$-mEGFP_bGH-PA term_EF1α_FLAG-SV40NLS-Cas9-NLS-T2A-BSD-WPRE-SV40	RKO cell expression of Kinprola$_{PKA}$ in cytosol and Cas9 in nucleus	#233371	NES-Kinprola$_{PKA}$-mEGFP, FLAG-SV40NLS-Cas9-NLS-T2A-BSD	RKO
pLenti_EF1α-NES-Kinprola$_{PKA\ T/A}$-mEGFP_bGH-PA term_EF1α_FLAG-SV40NLS-Cas9-NLS-T2A-BSD-WPRE-SV40	RKO cell expression of Kinprola$_{PKA\ T/A}$ in cytosol and Cas9 in nucleus	#233372	NES-Kinprola$_{PKA\ T/A}$-mEGFP, FLAG-SV40NLS-Cas9-NLS-T2A-BSD	RKO
pLenti_EF1α-NES-Kinprola$_{on}$-mEGFP_bGH-PA term_EF1α_FLAG-SV40NLS-Cas9-NLS-T2A-BSD-WPRE-SV40	RKO cell expression of Kinprola$_{on}$ in cytosol and Cas9 in nucleus	#233373	NES-Kinprola$_{on}$-mEGFP, FLAG-SV40NLS-Cas9-NLS-T2A-BSD	RKO
HD CRISPR library sub-library A	CRISPR screen	n.a.	genome-scale sgRNA library	n.a.
HDCRISPRv1_U6_sg*FZR1*-1	sgRNA expression for *FZR1* knockout	n.a.	sg*FZR1*-1	n.a.
HDCRISPRv1_U6_sg*FZR1*-2	sgRNA expression for *FZR1* knockout	n.a.	sg*FZR1*-2	n.a.
HDCRISPRv1_U6_sg*TRIM33*-1	sgRNA expression for *TRIM33* knockout	n.a.	sg*TRIM33*-1	n.a.
HDCRISPRv1_U6_sg*TRIM33*-2	sgRNA expression for *TRIM33* knockout	n.a.	sg*TRIM33*-2	n.a.
HDCRISPRv1_U6_sg*PES1*-1	sgRNA expression for *PES1* knockout	n.a.	sg*PES1*-1	n.a.
HDCRISPRv1_U6_sg*PES1*-2	sgRNA expression for *PES1* knockout	n.a.	sg*PES1*-2	n.a.
HDCRISPRv1_U6_sg*NOP2*-1	sgRNA expression for *NOP2* knockout	n.a.	sg*NOP2*-1	n.a.
HDCRISPRv1_U6_sg*NOP2*-2	sgRNA expression for *NOP2* knockout	n.a.	sg*NOP2*-2	n.a.

HDCRISPRv1_U6_sg*VPRBP*-1	sgRNA expression for *VPRBP* knockout	n.a.	sg*VPRBP*-1	n.a.
HDCRISPRv1_U6_sg*VPRBP*-2	sgRNA expression for *VPRBP* knockout	n.a.	sg*VPRBP*-2	n.a.
HDCRISPRv1_U6_sg*Non-targeting*-1	sgRNA expression for non-targeting sequence	n.a.	sg*Non-targeting*-1	n.a.
HDCRISPRv1_U6_sg*Non-targeting*-2	sgRNA expression for non-targeting sequence	n.a.	sg*Non-targeting*-2	n.a.

Supplementary Table 6 | sgRNA sequences selected from HD CRISPR sub-library A.

Gene ID	Gene name	sgRNA sequence 1	sgRNA sequence 2
ENSG00000105325	*FZR1*	CCGCTCAGACCAGCCCACGG	CAGCAGCTCATTCTTGAGCA
ENSG00000197323	*TRIM33*	CTTGCAGAGCCGGCGTGAGG	TTACTAAAGATCACTTGATC
ENSG00000100029	*PES1*	GCATGAACTCCACAGTGAGC	GCTGTGCCGCCGGCTCACTG
ENSG00000111641	*NOP2*	GTATTGGTCCGGAGGGTGAC	GAAGATGGTATGGTGAACCA
ENSG00000145041	*VPRBP*	ACATGGTACCTATCCTTACC	TATTCATACCTGGTAAGGAT
n.a.	*Non-targeting*	GTGACTAGACCCTTACGCGG	GATCGGCGGGTTACCTCTGA

Supplementary Table 7 | Primers for amplifying the genomic regions targeted by individual sgRNAs.

sgRNA name	Forward primer	Reverse primer
sgFZR1-1	GTGGAGGGATGAATGTACCCATGG	CCACTGGACCTGCCATCCTTTC
sgFZR1-2	ACAGGATTAACGTGAGGGGCTG	CCGTGAACAGACCCTTCTTCTCAG
sgTRIM33-1	TTCATCTCCTGCGGCCTCAG	CAGCTTCTCTGGAAAGTGTCCCAG
sgTRIM33-2	TCTGCACAGACTTCGTGTTCTCAG	ACCAGATGCTCCAACAGACTCTGTAG
sgPES1-1	GCATCCTGGTTAAGTGGTTCCTCC	GAGACATCTGGGTGAAGACCATGG
sgPES1-2	TTAAGTGGTTCCTCCTAGGGGCTG	ATGCCTTTGATGGACAGGAAGACC
sgNOP2-1	TTGGCAAGCTCATGGACCTCTTC	AGAGACATCAGGCCTCTGGGTTTG
sgNOP2-2	TGAGGGGTAGAAAGTACAGGCTCC	CCTCTAGTCAGGCTTATCCTTGCC
sgVPRBP-1, -2	GCCATGACTACAGTAGTGGTACATGTGG	ACAGCTTCAGGAAGTAGGGAGATAAAGAC

Supplementary Table 8 | Imaging acquisition parameters for fluorescence microscopy.

Figure	Construct	Label	Microscope	Objective	Excitation (nm)	Emission (nm)	Fluorophore concentration
**1c**	NES-Kinprola$_{PKA}$-mEGFP	mEGFP<br>CPY	Leica Stellaris 5 confocal	20× water<br>0.75-NA	488<br>610	498-550<br>620-720	-<br>25 nM
**1f**	NES-Kinprola$_{PKA}$-mEGFP	mEGFP<br>CPY	Leica Stellaris 5 confocal	20× water<br>0.75-NA	488<br>610	498-550<br>620-720	-<br>25 nM
**1h**	NES-Kinprola$_{PKA}$-mEGFP	mEGFP<br>JF_{552}<br>CPY<br>JF_{669}	Leica Stellaris 5 confocal	20× water<br>0.75-NA	488<br>550<br>610<br>669	498-550<br>560-600<br>620-660<br>679-750	-<br>100 nM<br>25 nM<br>100 nM
**4a**	NES-Kinprola$_{PKA}$-mEGFP<br>NES-Kinprola$_{PKA_T/A}$-mEGFP<br>NES-Kinprola$_{on}$-mEGFP	mEGFP<br>CPY	Leica Stellaris 5 confocal	20× water<br>0.75-NA	488<br>610	498-550<br>620-720	-<br>25 nM
**4c**	NES-Kinprola$_{PKA}$-mEGFP	mEGFP<br>CPY	Leica Stellaris 5 confocal	20× water<br>0.75-NA	488<br>610	498-550<br>620-720	-<br>125 nM
**5b,c**	NES-Kinprola$_{PKA}$-mEGFP<br>NES-Kinprola$_{PKA_T/A}$-mEGFP	mEGFP<br>CPY	ZEISS LSM 710 upright confocal	20× water<br>0.5-NA	488<br>633	498-550<br>638-747	-<br>250 nM
**5d**	NES-Kinprola$_{PKA}$-mEGFP<br>NES-Kinprola$_{PKA_T/A}$-mEGFP	mEGFP<br>CPY	Leica SP8X	40× oil<br>1.3-NA	488<br>610	498-550<br>620-720	-<br>250 nM
**5h**	NES-Kinprola$_{PKA}$-mEGFP	mEGFP<br>CPY	Leica SP8X	20× dry<br>0.7-NA	488<br>610	498-550<br>620-720	-<br>100 nmol
**Extended Data Fig. 5**	NES-Kinprola$_{PKA}$-mEGFP	mEGFP<br>JF_{552}<br>CPY<br>JF_{669}	Leica Stellaris 5 confocal	20× water<br>0.75-NA	488<br>550<br>610<br>669	498-550<br>560-600<br>620-660<br>679-750	-<br>100 nM<br>25 nM<br>100 nM
**Extended Data Fig. 6d**	NES-Kinprola$_{PKA}$-mTagBFP2<br>Kinprola$_{PKA}$-mEGFP-NLS3×	mTagBFP2<br>mEGFP<br>CPY	Leica Stellaris 5 confocal	40× water<br>1.1-NA	405<br>488<br>612	415-480<br>498-550<br>620-700	-<br>-<br>50 nM
**Extended Data Fig. 9a,e**	NES-Kinprola$_{PKA}$-mEGFP	mEGFP<br>CPY	Leica Stellaris 5 confocal	20× water<br>0.75-NA	488<br>610	498-550<br>620-720	-<br>125 nM
**Extended Data Fig. 9b**	NES-Kinprola$_{PKA}$-mEGFP	mEGFP<br>CPY	Leica Stellaris 5 confocal	20× water<br>0.75-NA	488<br>610	498-550<br>620-720	-<br>25 nM
**Supplementary Fig. 2**	NES-Kinprola$_{PKA}$-mEGFP	mEGFP	Leica Stellaris 5 confocal	20× water	488	498-550	-

		CPY		0.75-NA	610	620-720	25 nM
**Supplementary Fig. 4**	NES-Kinprola$_{PKA}$-mEGFP	mEGFP	Leica Stellaris 5 confocal	20× water	488	498-550	-
		CPY		0.75-NA	610	620-720	10, 25, 125 nM
**Supplementary Fig. 10a**	NES-Kinprola$_{PKA}$-mEGFP	mEGFP	Leica Stellaris 5 confocal	20× water	488	498-550	-
		CPY		0.75-NA	610	620-720	125 nM
**Supplementary Fig. 11b**	NES-Kinprola$_{PKA}$-mEGFP	mEGFP	Leica Stellaris 5 confocal	20× water	488	498-550	-
		CPY		0.75-NA	610	620-720	125 nM
**Supplementary Fig. 12a**	NES-Kinprola$_{PKA}$-mEGFP	mEGFP	Leica SP8X	20× dry	488	498-550	-
		CPY		0.7-NA	610	620-720	100 nmol

Supplementary Note

Supplementary Note 1 | Protein sequences

>Kinprola$_{PKA}$
Elements: NES-cpHaloΔ-PKA substrate-cpFHA1-Hpep-mEGFP
MLQNELALKLAGLDINKTGGSDVGRKLIIDQNVFIEGTLPMGVVRPLTEVEMDHYREPFLNPVDREPL
WRFPNELPIAGEPANIVALVEEYMDWLHQSPVPKLLFWGTPGVLIPPAEAARLAKSLPNCKAVDIGPG
LNLLQEDNPDLIGSEIARWLSTLEIGGTGGSGGTGGSGGSIGTGFPFDPHYVEVLGERMHYVDVGPRD
GTPVLFLHGNPTSSYVWRNIIPHVAPTHRCIAPDLIGMGKSDKPDLGYFFDDHVRFMDAFIEALGLEE
VVLVIHDWGSALGFHWAKRNPERVKGIAFMEFIRPIPTWDEWAPGSLRRATLVDPPPPPPPPPPPPPP
PPPPPPPPPPPPPPPPGGSACDYHLGNISRLSNKHFQILLGEDGNLLLNDISTNGTWLNGQKVEKNSY
QLLSQGDEITVGVGVESDILSLVIFINDKFKQCLEQNKVDRGGGMHKFSQEQIGENIVCRVICTTGQI
PIRDLSADISQVLKEKRSIKKVWTFGRNPGSAKETFQAFRSGGSMVSKGEELFTGVVPILVELDGDVN
GHKFSVSGEGEGDATYGKLTLKFICTTGKLPVPWPTLVTTLTYGVQCFSRYPDHMKQHDFFKSAMPEG
YVQERTIFFKDDGNYKTRAEVKFEGDTLVNRIELKGIDFKEDGNILGHKLEYNYNSHNVYIMADKQKN
GIKVNFKIRHNIEDGSVQLADHYQQNTPIGDGPVLLPDNHYLSTQSKLSKDPNEKRDHMVLLEFVTAA
GITLGMDELYK

>Kinprola$_{PKA_T/A}$
Elements: NES-cpHaloΔ-PKA substrate T/A-cpFHA1-Hpep-mEGFP
MLQNELALKLAGLDINKTGGSDVGRKLIIDQNVFIEGTLPMGVVRPLTEVEMDHYREPFLNPVDREPL
WRFPNELPIAGEPANIVALVEEYMDWLHQSPVPKLLFWGTPGVLIPPAEAARLAKSLPNCKAVDIGPG
LNLLQEDNPDLIGSEIARWLSTLEIGGTGGSGGTGGSGGSIGTGFPFDPHYVEVLGERMHYVDVGPRD
GTPVLFLHGNPTSSYVWRNIIPHVAPTHRCIAPDLIGMGKSDKPDLGYFFDDHVRFMDAFIEALGLEE
VVLVIHDWGSALGFHWAKRNPERVKGIAFMEFIRPIPTWDEWAPGSLRRAALVDPPPPPPPPPPPPPP
PPPPPPPPPPPPPPPPGGSACDYHLGNISRLSNKHFQILLGEDGNLLLNDISTNGTWLNGQKVEKNSY
QLLSQGDEITVGVGVESDILSLVIFINDKFKQCLEQNKVDRGGGMHKFSQEQIGENIVCRVICTTGQI
PIRDLSADISQVLKEKRSIKKVWTFGRNPGSAKETFQAFRSGGSMVSKGEELFTGVVPILVELDGDVN
GHKFSVSGEGEGDATYGKLTLKFICTTGKLPVPWPTLVTTLTYGVQCFSRYPDHMKQHDFFKSAMPEG
YVQERTIFFKDDGNYKTRAEVKFEGDTLVNRIELKGIDFKEDGNILGHKLEYNYNSHNVYIMADKQKN
GIKVNFKIRHNIEDGSVQLADHYQQNTPIGDGPVLLPDNHYLSTQSKLSKDPNEKRDHMVLLEFVTAA
GITLGMDELYK

>Kinprola$_{on}$
Elements: NES-cpHalo-PKA substrate-cpFHA1-Hpep-mEGFP
MLQNELALKLAGLDINKTGGSFARETFQAFRTTDVGRKLIIDQNVFIEGTLPMGVVRPLTEVEMDHYR
EPFLNPVDREPLWRFPNELPIAGEPANIVALVEEYMDWLHQSPVPKLLFWGTPGVLIPPAEAARLAKS
LPNCKAVDIGPGLNLLQEDNPDLIGSEIARWLSTLEIGGTGGSGGTGGSGGSIGTGFPFDPHYVEVLG
ERMHYVDVGPRDGTPVLFLHGNPTSSYVWRNIIPHVAPTHRCIAPDLIGMGKSDKPDLGYFFDDHVRF
MDAFIEALGLEEVVLVIHDWGSALGFHWAKRNPERVKGIAFMEFIRPIPTWDEWAPGSLRRATLVDPP
PPPPPPPPPPPPPPPPPPPPPPPPPPPPGGSACDYHLGNISRLSNKHFQILLGEDGNLLLNDISTNGT
WLNGQKVEKNSYQLLSQGDEITVGVGVESDILSLVIFINDKFKQCLEQNKVDRGGGMHKFSQEQIGEN
IVCRVICTTGQIPIRDLSADISQVLKEKRSIKKVWTFGRNPGSAKETFQAFRSGGSMVSKGEELFTGV
VPILVELDGDVNGHKFSVSGEGEGDATYGKLTLKFICTTGKLPVPWPTLVTTLTYGVQCFSRYPDHMK
QHDFFKSAMPEGYVQERTIFFKDDGNYKTRAEVKFEGDTLVNRIELKGIDFKEDGNILGHKLEYNYNS
HNVYIMADKQKNGIKVNFKIRHNIEDGSVQLADHYQQNTPIGDGPVLLPDNHYLSTQSKLSKDPNEKR
DHMVLLEFVTAAGITLGMDELYK

>Kinprola$_{off}$
Elements: NES-cpHaloΔ-PKA substrate-cpFHA1-mEGFP
MLQNELALKLAGLDINKTGGSFARETFQAFRTTDVGRKLIIDQNVFIEGTLPMGVVRPLTEVEMDHYR
EPFLNPVDREPLWRFPNELPIAGEPANIVALVEEYMDWLHQSPVPKLLFWGTPGVLIPPAEAARLAKS
LPNCKAVDIGPGLNLLQEDNPDLIGSEIARWLSTLEIGGTGGSGGTGGSGGSIGTGFPFDPHYVEVLG
ERMHYVDVGPRDGTPVLFLHGNPTSSYVWRNIIPHVAPTHRCIAPDLIGMGKSDKPDLGYFFDDHVRF

MDAFIEALGLEEVVLVIHDWGSALGFHWAKRNPERVKGIAFMEFIRPIPTWDEWAPGSLRRATLVDPP
PPPPPPPPPPPPPPPPPPPPPPPPPPPPGGSACDYHLGNISRLSNKHFQILLGEDGNLLLNDISTNGT
WLNGQKVEKNSYQLLSQGDEITVGVGVESDILSLVIFINDKFKQCLEQNKVDRGGGMHKFSQEQIGEN
IVCRVICTTGQIPIRDLSADISQVLKEKRSIKKVWTFGRNPGGSMVSKGEELFTGVVPILVELDGDVN
GHKFSVSGEGEGDATYGKLTLKFICTTGKLPVPWPTLVTTLTYGVQCFSRYPDHMKQHDFFKSAMPEG
YVQERTIFFKDDGNYKTRAEVKFEGDTLVNRIELKGIDFKEDGNILGHKLEYNYNSHNVYIMADKQKN
GIKVNFKIRHNIEDGSVQLADHYQQNTPIGDGPVLLPDNHYLSTQSKLSKDPNEKRDHMVLLEFVTAA
GITLGMDELYK

>mTagBFP2 (C-terminally fused)
MVSKGEELIKENMHMKLYMEGTVDNHHFKCTSEGEGKPYEGTQTMRIKVVEGGPLPFAFDILATSFLY
GSKTFINHTQGIPDFFKQSFPEGFTWERVTTYEDGGVLTATQDTSLQDGCLIYNVKIRGVNFTSNGPV
MQKKTLGWEAFTETLYPADGGLEGRNDMALKLVGGSHLIANAKTTYRSKKPAKNLKMPGVYYVDYRLE
RIKEANNETYVEQHEVAVARYCDLPSKLGHKLN

>Cas9 expression
Elements: FLAG-SV40 NLS-Cas9-nucleoplasmin NLS-T2A-BSD
MDYKDDDDKMAPKKKRKVGIHGVPAADKKYSIGLDIGTNSVGWAVITDEYKVPSKKFKVLGNTDRHSI
KKNLIGALLFDSGETAEATRLKRTARRRYTRRKNRICYLQEIFSNEMAKVDDSFFHRLEESFLVEEDK
KHERHPIFGNIVDEVAYHEKYPTIYHLRKKLVDSTDKADLRLIYLALAHMIKFRGHFLIEGDLNPDNS
DVDKLFIQLVQTYNQLFEENPINASGVDAKAILSARLSKSRRLENLIAQLPGEKKNGLFGNLIALSLG
LTPNFKSNFDLAEDAKLQLSKDTYDDDLDNLLAQIGDQYADLFLAAKNLSDAILLSDILRVNTEITKA
PLSASMIKRYDEHHQDLTLLKALVRQQLPEKYKEIFFDQSKNGYAGYIDGGASQEEFYKFIKPILEKM
DGTEELLVKLNREDLLRKQRTFDNGSIPHQIHLGELHAILRRQEDFYPFLKDNREKIEKILTFRIPYY
VGPLARGNSRFAWMTRKSEETITPWNFEEVVDKGASAQSFIERMTNFDKNLPNEKVLPKHSLLYEYFT
VYNELTKVKYVTEGMRKPAFLSGEQKKAIVDLLFKTNRKVTVKQLKEDYFKKIECFDSVEISGVEDRF
NASLGTYHDLLKIIKDKDFLDNEENEDILEDIVLTLTLFEDREMIEERLKTYAHLFDDKVMKQLKRRR
YTGWGRLSRKLINGIRDKQSGKTILDFLKSDGFANRNFMQLIHDDSLTFKEDIQKAQVSGQGDSLHEH
IANLAGSPAIKKGILQTVKVVDELVKVMGRHKPENIVIEMARENQTTQKGQKNSRERMKRIEEGIKEL
GSQILKEHPVENTQLQNEKLYLYYLQNGRDMYVDQELDINRLSDYDVDHIVPQSFLKDDSIDNKVLTR
SDKNRGKSDNVPSEEVVKKMKNYWRQLLNAKLITQRKFDNLTKAERGGLSELDKAGFIKRQLVETRQI
TKHVAQILDSRMNTKYDENDKLIREVKVITLKSKLVSDFRKDFQFYKVREINNYHHAHDAYLNAVVGT
ALIKKYPKLESEFVYGDYKVYDVRKMIAKSEQEIGKATAKYFFYSNIMNFFKTEITLANGEIRKRPLI
ETNGETGEIVWDKGRDFATVRKVLSMPQVNIVKKTEVQTGGFSKESILPKRNSDKLIARKKDWDPKKY
GGFDSPTVAYSVLVVAKVEKGKSKKLKSVKELLGITIMERSSFEKNPIDFLEAKGYKEVKKDLIIKLP
KYSLFELENGRKRMLASAGELQKGNELALPSKYVNFLYLASHYEKLKGSPEDNEQKQLFVEQHKHYLD
EIIEQISEFSKRVILADANLDKVLSAYNKHRDKPIREQAENIIHLFTLTNLGAPAAFKYFDTTIDRKR
YTSTKEVLDATLIHQSITGLYETRIDLSQLGGDKRPAATKKAGQAKKKKASGSGEGRGSLLTCGDVEE
NPGPMAKPLSQEESTLIERATATINSIPISEDYSVASAALSSDGRIFTGVNVYHFTGGPCAELVVLGT
AAAAAAGNLTCIVAIGNENRGILSPCGRCRQVLLDLHPGIKAIVKDSDGQPTAVGIRELLPSGYVWEG

Different kinases	Substrate peptides	T/A mutants
PKC	RFRRFQTLKDKAKA	RFRRFQALKDKAKA
JNK	DSVKTPEDEGNPLLEQLEKK	DSVKAPEDEGNPLLEQLEKK
AMPK	MRRVATLVDL	MRRVAALVDL

Localization sequences

>Nuclear export sequence (NES, N-terminally fused)
(M) LQNELALKLAGLDINKT
>Nuclear localization sequence (NLS, C-terminally fused, 3 copies)
KSGLRSRADPKKKRKVDPKKKRKVDPKKKRKVGSTGSR
>Microtubule targeting sequence (MTBD, microtubule-binding domain of MAP2c, C-terminally fused)

SGVYKGTPGTPKSAILVPSEKKVAIIRTPPKSPATPKQLRLINQPLPDLKNVKSKIGSTDNIKYQPKG
GQVQIVTKKIDLSHVTSKCGSLKNIRHRPGGGRVKIESVKLDFKEKAQAKVGSLDNAHHVPGGGNVKI
DSQKLNFREHAKARVDHGAEIITQSPGRSSVASPRRLSNVSSSGSINLLESPQLATLAEDVTAALAKQ
GL

Purification sequences

>Poly-histidine tag + TEV cleavage sequence (N-terminal)
HHHHHHHHHHENLYFQGG (pET-51b(+) plasmid)

Supplementary Note 2 | Uncropped scans of all blots

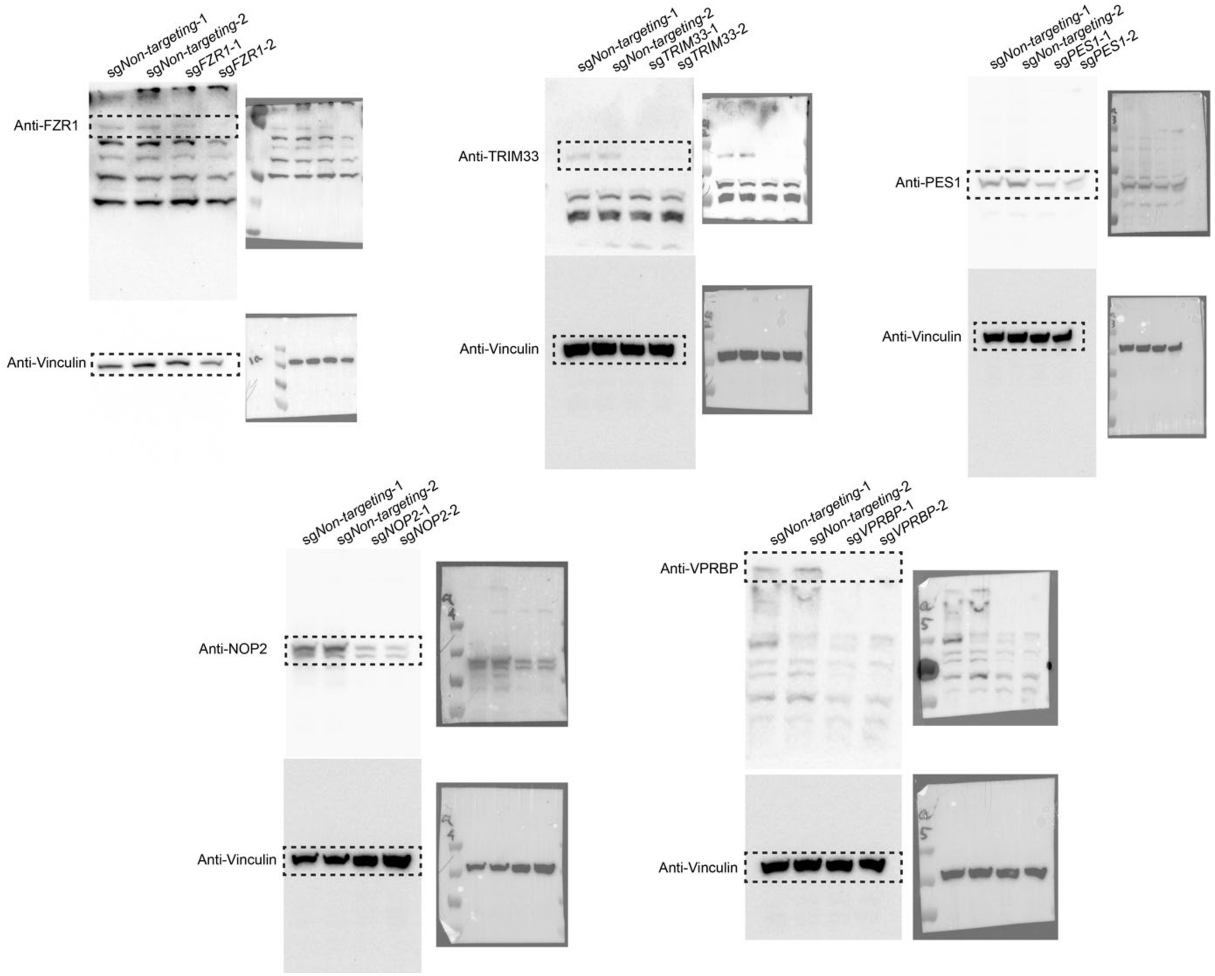

Supplement: Supplementary file 1 — Supplementary Figs. 1–12, Tables 1–8 and Notes 1 and 2. [file 41589_2025_1949_MOESM1_ESM.pdf]
