# Supplementary material for: Molecular recording of cellular protein kinase activity with chemical labeling
Source: Nat Chem Biol. 2025 Jul 10;21(11):1818–27. doi: 10.1038/s41589-025-01949-6 (PMC12568631; doi:10.1038/s41589-025-01949-6)
Supplement: Supplementary file 2 — Reporting Summary [file 41589_2025_1949_MOESM2_ESM.pdf]

nature portfolio

Corresponding author(s): Dr. De-en Sun, Prof. Dr. Kai Johnsson

Last updated by author(s): May 8, 2025

Reporting Summary

Nature Portfolio wishes to improve the reproducibility of the work that we publish. This form provides structure for consistency and transparency in reporting. For further information on Nature Portfolio policies, see our Editorial Policies and the Editorial Policy Checklist.

Statistics

For all statistical analyses, confirm that the following items are present in the figure legend, table legend, main text, or Methods section.

n/a	Confirmed	
☐	☒	The exact sample size (*n*) for each experimental group/condition, given as a discrete number and unit of measurement
☐	☒	A statement on whether measurements were taken from distinct samples or whether the same sample was measured repeatedly
☐	☒	The statistical test(s) used AND whether they are one- or two-sided<br>*Only common tests should be described solely by name; describe more complex techniques in the Methods section.*
☒	☐	A description of all covariates tested
☐	☒	A description of any assumptions or corrections, such as tests of normality and adjustment for multiple comparisons
☐	☒	A full description of the statistical parameters including central tendency (e.g. means) or other basic estimates (e.g. regression coefficient) AND variation (e.g. standard deviation) or associated estimates of uncertainty (e.g. confidence intervals)
☐	☒	For null hypothesis testing, the test statistic (e.g. *F*, *t*, *r*) with confidence intervals, effect sizes, degrees of freedom and *P* value noted<br>*Give P values as exact values whenever suitable.*
☒	☐	For Bayesian analysis, information on the choice of priors and Markov chain Monte Carlo settings
☒	☐	For hierarchical and complex designs, identification of the appropriate level for tests and full reporting of outcomes
☒	☐	Estimates of effect sizes (e.g. Cohen's *d*, Pearson's *r*), indicating how they were calculated

Our web collection on statistics for biologists contains articles on many of the points above.

Software and code

Policy information about availability of computer code

Data collection	Plate reader: Tecan Sparkcontrol Method Editor Version 2.2<br>Confocal Microscopy: Stellaris 5 (Leica), upright LSM 710 confocal microscope (ZEISS), SP8X confocal microscope (Leica)<br>FACS: BD FACSMelody Cell Sorter, BD FACSAria Fusion Special Order System<br>Flow cytometry: BD Fortessa X-20 Cell Analyzer<br>Thermal stability: Prometheus NT48 nanoscale differential scanning fluorimeter<br>Western blot: ChemiDoc MP imager (Bio-Rad)<br>RNA sequencing: NovaSeq6000 device (Illumina), STAR56 (v.2.5.3a), GENCODE (v.32), DeSeq2 (v1.38.3), ShinyGO (RRID:SCR_019213, v.0.741 and v.0.80)<br>CRISPR screen: Illumina NextSeq 500/550 system, MAGeCK version 0.5.9.4 package<br>Schematic elements: BioRender (https://www.biorender.com/)
Data analysis	ImageJ/Fiji (version 2.9.0/1.54h), FlowJo suite (version 10.10.0), Image Lab software (Bio-Rad, version 6.1.0), Excel (version 16.78.3), R package (version 4.3.1, 2023-06-16), OriginPro 2020b (OriginLab), GraphPad Prism (version 10.2.1).

For manuscripts utilizing custom algorithms or software that are central to the research but not yet described in published literature, software must be made available to editors and reviewers. We strongly encourage code deposition in a community repository (e.g. GitHub). See the Nature Portfolio guidelines for submitting code & software for further information.

Data

Policy information about availability of data

All manuscripts must include a data availability statement. This statement should provide the following information, where applicable:

- Accession codes, unique identifiers, or web links for publicly available datasets
- A description of any restrictions on data availability
- For clinical datasets or third party data, please ensure that the statement adheres to our policy

All data are available in the paper or the supplementary materials. Plasmids of interest from the study have been deposited in Addgene, and accession codes are provided in the supplementary information. GRCh38 human reference genome was downloaded under (https://www.ncbi.nlm.nih.gov/datasets/genome/GCF_000001405.26/). Raw RNA-Seq data and raw sequencing data of CRISPR screen have been deposited in NCBI's Gene Expression Omnibus and are accessible through GEO Series accession numbers GSE269419 (https://www.ncbi.nlm.nih.gov/geo/query/acc.cgi?acc=GSE269419) and GSE277987 (https://www.ncbi.nlm.nih.gov/geo/query/acc.cgi?acc=GSE277987). Reagents and materials are available from the corresponding authors upon request.

Human research participants

Policy information about studies involving human research participants and Sex and Gender in Research.

Reporting on sex and gender	N/A
Population characteristics	N/A
Recruitment	N/A
Ethics oversight	N/A

Note that full information on the approval of the study protocol must also be provided in the manuscript.

Field-specific reporting

Please select the one below that is the best fit for your research. If you are not sure, read the appropriate sections before making your selection.

☒ Life sciences ☐ Behavioural & social sciences ☐ Ecological, evolutionary & environmental sciences

For a reference copy of the document with all sections, see nature.com/documents/nr-reporting-summary-flat.pdf

Life sciences study design

All studies must disclose on these points even when the disclosure is negative.

Sample size	Sample sizes were based on experiences in prior similar studies or samples were acquired until a clear trend was evident.<br>e.g., Science 383, 890–897 (2024); Nat. Chem. Biol. 20, 894–905 (2024); Nat. Methods 21, 1725–1735 (2024); Nat. Commun. 15, 7804 (2024).
Data exclusions	For RNA sequencing analysis, genes with fewer than 10 total counts across all samples were excluded, consistent with the filtering criteria used in similar studies by our group and collaborators. e.g, Science 383, 890–897 (2024); Nat. Commun. 15, 968 (2024).<br>No other data was excluded.
Replication	All replicates were successful. The exact number of replicates is indicated in the corresponding figure legends.
Randomization	Cells, slices and animals were randomly allocated into experimental groups.
Blinding	ELISA and genotyping experiments were conducted by a separate experimenter who was blinded to the experimental conditions. For other data collection and analysis, investigators were not blinded, as the experimental conditions were inherently obvious to the researchers and the analysis was performed objectively and not subjective to human bias.

Reporting for specific materials, systems and methods

We require information from authors about some types of materials, experimental systems and methods used in many studies. Here, indicate whether each material, system or method listed is relevant to your study. If you are not sure if a list item applies to your research, read the appropriate section before selecting a response.

Materials & experimental systems

n/a	Involved in the study
☐	☒ Antibodies
☐	☒ Eukaryotic cell lines
☒	☐ Palaeontology and archaeology
☐	☒ Animals and other organisms
☒	☐ Clinical data
☒	☐ Dual use research of concern

Methods

n/a	Involved in the study
☒	☐ ChIP-seq
☐	☒ Flow cytometry
☒	☐ MRI-based neuroimaging

Antibodies

Antibodies used

Commercial primary antibodies:
FZR1/Cdh1 Polyclonal antibody (Proteintech, 16368-1-AP, Lot no. 00041419),
TRIM33 Polyclonal antibody (Proteintech, 55374-1-AP, Lot no. 09000804),
PES1 Polyclonal antibody (Proteintech, 13553-1-AP, Lot no. 00054973),
VPRBP Polyclonal antibody (Proteintech, 11612-1-AP Lot no. 00085369),
NOP2 Polyclonal antibody (Proteintech, 10448-1-AP, Lot no. 00021873),
Anti-Vinculin antibody (Sigma-Aldrich, V9264, Lot no. 0000312702)

Commercial secondary antibodies:
Horseradish peroxidase (HRP)-AffiniPure Polyclonal Goat Anti-Mouse IgG (H+L) (Jackson ImmunoResearch, JIM-115-035-003, Lot no. 174329),
HRP-AffiniPure Polyclonal Goat Anti-Rabbit IgG (H+L) (Jackson ImmunoResearch, JIM-111-035-003, Lot no. 174633)

Validation

All antibodies are validated by vendors indicated above. Many references are listed on respective manufacture's websites. Below we list one reference for each antibody.
FZR1/Cdh1 Polyclonal antibody, DOI: 10.1126/sciadv.abg9335
TRIM33 Polyclonal antibody, DOI: 10.1038/s41467-021-26807-6
PES1 Polyclonal antibody, DOI: 10.1186/s13046-021-02162-8
VPRBP Polyclonal antibody, DOI: 10.1016/j.molcel.2018.05.007
NOP2 Polyclonal antibody, DOI: 10.1016/j.celrep.2023.113280
Anti-Vinculin antibody, DOI: 10.1038/s41467-021-27077-y
HRP-AffiniPure Polyclonal Goat Anti-Mouse IgG (H+L), DOI: 10.1038/s41467-025-56675-3
HRP-AffiniPure Polyclonal Goat Anti-Rabbit IgG (H+L), DOI: 10.1038/s41589-024-01614-4

Eukaryotic cell lines

Policy information about cell lines and Sex and Gender in Research

Cell line source(s)

HeLa Kyoto Flp-In cell (RRID:CVCL_192219, from Cell Biology and Biophysics Unit, European Molecular Biology Laboratory, Heidelberg, Germany), Cell 126, 473–484 (2009); Science 383, 890–897 (2024).
HEK293 cell from Leibniz Institute DSMZ German Collection of Microorganisms and Cell Cultures (ACC-305)
HEK293 Flp-In-TREx cell from Thermo Fisher Scientific (R78007)
Patient derived glioblastoma cell line (PDGCL) S24 was established in CCU Neurooncology, German Cancer Research Center, Heidelberg. J. Neurochem. 131, 251–264 (2014).
RKO cell from ATCC (CRL-2577)

Authentication

S24 (human, female) was regularly checked for authenticity and absence of infections, such as mycoplasma and non-human cell contamination, as part of the multiplex cell contamination test (Multiplexion GmbH). S24 was further authenticated as GB by 850k methylation EPIC array (#WG-317-1003, Illumina) as described in Cell 185, 2899–2917.e31 (2022); Nature 613, 179–186 (2023).
The other cell lines were not further authenticated.

Mycoplasma contamination

Cell lines have been tested by PCR regularly and were negative.

Commonly misidentified lines
(See ICLAC register)

No commonly misidentified cell lines were used.

Animals and other research organisms

Policy information about studies involving animals; ARRIVE guidelines recommended for reporting animal research, and Sex and Gender in Research

Laboratory animals

Postnatal P0–P1 Wistar rats of both sexes were used for neuron preparation. Male C57BL/6N mice (6–8 weeks of age) were used for acute brain slice preparation and in vivo labeling. Mice were group- or pair-housed in a temperature-controlled (18–23°C) and humidity-controlled (40–60%) room with a 12-h light/dark cycle. Food and water were available ad libitum.

Wild animals

The study did not involve wild animals.

Reporting on sex	Animal sex was not considered a biological variable in the study design, as the primary goal was to demonstrate a new method rather than report biological findings. For the available and consistent supply, rats of both sexes were used for neuron preparation, and male mice were used during the acute brain slice and in vivo labeling experimental periods.
Field-collected samples	The study did not involve samples collected from the field.
Ethics oversight	For primary rat hippocampal neurons preparation, all procedures were conducted in strict accordance with the Animal Welfare Act of the Federal Republic of Germany (Tierschutzgesetz der Bundesrepublik Deutschland, TierSchG) and the Animal Welfare Laboratory Animal Regulations (Tierschutzversuchsverordnung). According to these regulations, no ethical approval from an ethics committee is required for euthanizing rodents when the organs or tissues are used for scientific purposes. The euthanasia procedure for rats in this study was supervised by animal welfare officers of the Max Planck Institute for Medical Research and was carried out and documented in compliance with the TierSchG (permit number assigned by the Max Planck Institute for Medical Research: MPI/T-35/18).<br>For acute brain slice and in vivo labeling, all procedures for animal surgery and experimentation were performed using protocols approved by the Institutional Animal Care and Use Committee at Peking University.

Note that full information on the approval of the study protocol must also be provided in the manuscript.

Flow Cytometry

Plots

Confirm that:

☒ The axis labels state the marker and fluorochrome used (e.g. CD4-FITC).

☒ The axis scales are clearly visible. Include numbers along axes only for bottom left plot of group (a 'group' is an analysis of identical markers).

☒ All plots are contour plots with outliers or pseudocolor plots.

☒ A numerical value for number of cells or percentage (with statistics) is provided.

Methodology

Sample preparation	Samples for flow cytometry and FACS were prepared as stated in the corresponding method sections.
Instrument	BD Fortessa X-20 Cell Analyzer, BD FACSMelody Cell Sorter, BD FACSAria Fusion Special Order System
Software	FlowJo (version 10.10.0)
Cell population abundance	Cell population abundance for RNA-Seq and CRISPR screen is described in the method section.
Gating strategy	Hierarchical gating of live cells (SSC-A/FSC-A), single cells (SSC-H/SSC-A) and labeling (label channel/expression channel). Gating strategy is described in the method section and exemplified in Extended Figure 8b, Supplementary Figures 3 and 5.

☒ Tick this box to confirm that a figure exemplifying the gating strategy is provided in the Supplementary Information.
